# Towards nature-related risk disclosures in China

**DOI:** 10.1093/biosci/biag071

**Published:** 2026-06-18

**Authors:** Carla L Archibald, Lei Gao, Michalis Hadjikakou, Cai Li, Jianguo Liu, Emily Nicholson, Weixin Ou, Qin Tao, Zhen Wang, Xiaoling Zhang, Chen Zeng, Brett A Bryan

**Affiliations:** School of Life and Environmental Sciences, Deakin University, Burwood, Victoria 3125, Australia; Commonwealth Scientific and Industrial Research Organisation (CSIRO), Waite Campus, Urrbrae SA 5064, Australia; School of Life and Environmental Sciences, Deakin University, Burwood, Victoria 3125, Australia; School of Life and Environmental Sciences, Deakin University, Burwood, Victoria 3125, Australia; Interdisciplinary Research Center for Territorial Spatial Governance and Green Development, Huazhong Agricultural University, Wuhan 430070, China; Center for Systems Integration and Sustainability, Department of Fisheries and Wildlife, Michigan State University, East Lansing, MI 48825, United States; School of Agriculture, Food and Ecosystem Sciences, The University of Melbourne, Parkville, Victoria 3052, Australia; School of Land Management, Nanjing Agricultural University, WeiGang 1, Nanjing 210095 Jiangsu, China; School of Land Management, Nanjing Agricultural University, WeiGang 1, Nanjing 210095 Jiangsu, China; Interdisciplinary Research Center for Territorial Spatial Governance and Green Development, Huazhong Agricultural University, Wuhan 430070, China; Department of Real Estate and Construction, Faculty of Architecture, The University of Hong Kong, Hong Kong, China; Sustainability X-Lab, Faculty of Architecture, The University of Hong Kong, Hong Kong, China; Interdisciplinary Research Center for Territorial Spatial Governance and Green Development, Huazhong Agricultural University, Wuhan 430070, China; School of Life and Environmental Sciences, Deakin University, Burwood, Victoria 3125, Australia

**Keywords:** corporate sustainability, environmental disclosure, nature risk, sustainable business, sustainability reporting

## Abstract

Nature plays a foundational role in most economic sectors, making the loss of nature a significant threat to sustainable business activities, including within China. Methods to assess and disclose dependencies and impacts of businesses on nature have been developed, although they face some inherent challenges, including data availability and quality, supply chain traceability, and navigating complex governance arrangements. Given the importance of China’s corporate sector in shaping global sustainability, and as its largest listed companies increasingly adopt nature-related risk disclosures and sustainability reporting, this paper presents strategies to overcome key challenges such as data gaps, supply chain complexity, and governance issues to support credible, effective action towards nature-related risk management in China.

Many businesses rely on nature in some way to function (Costanza et al. 2014). In 2023, the Taskforce on Nature-related Financial Disclosures (TNFD) published recommendations on how businesses can identify, disclose, and respond to “nature-related risks” (TNFD 2023a). TNFD guidance has generated considerable enthusiasm across many economic sectors globally, including within China, with its increasing national interest in environmental, social, and corporate governance (ESG) and green finance (Shen et al. 2023, Gao 2024, Zhang et al. 2024). Nature-related risk disclosures are particularly crucial considering the significant role of domestic and international trade to China’s economy and the long reach of its supply chain on global sustainability. However, overcoming critical bottlenecks related to locating, assessing, evaluating, and preparing nature-related risk assessments is central to realising the full benefits of nature-related risk disclosures and sustainability reporting in China (Chen et al. 2021, Lin et al. 2023).

Natural capital and ecosystem services are highly valuable to the economy and society (Chen and Zhang 2000, Costanza et al. 2014). Biological processes such as water purification, pollination by invertebrates, and pest control by insectivorous birds directly support agricultural productivity, water availability supports the construction industry, and intact ecosystems and species richness underpin the tourism sector (Kumar 2011). However, the natural environment has undergone significant challenges, including habitat loss, desertification, soil degradation, water scarcity, air pollution, and extreme weather events like droughts and floods, which have threatened these biological processes in many locations (Liu and Diamond 2005, Liu and Raven 2010, Tan et al. 2017). Understanding and protecting these biological processes is increasingly critical for businesses (Medrano et al. 2025), as declines in species and ecosystem functioning can directly affect supply chain operational resilience (Jin 2025).

Within China, environmental degradation has profound negative social and economic consequences. For example, expanding and poorly managed irrigation in already water-scarce regions drives agricultural water shortages (Liu and Diamond 2005, Liu and Raven 2010, Wang et al. 2023), and many energy projects overlap with critical habitats and Indigenous lands, potentially impacting up to 24% of the world’s threatened species, generating transition and reputational risks (Yang et al. 2021). These environmental stressors and impacts have the potential to jeopardise companies by compromising the resilience of their supply chains and could also compromise total output, particularly if these pressures were further exacerbated (Chen et al. 2021). The risks associated with nature loss on the financial outlook of companies are currently being overlooked within annual reporting procedures (e.g., sustainability reporting) in China. Despite this growing risk landscape, current sustainability reporting in China does not adequately capture companies’ exposure to nature loss.

Nature-related risk is the interplay between companies relying on natural capital and services for profitability while simultaneously negatively impacting biological processes and the environment, thereby creating a reciprocal relationship that can affect their dependencies (TNFD 2023a). Degradation of ecosystems and natural processes through factors such as climate change, deforestation, or pollution can expose companies to physical, transition, and reputational risks, for example, through supply chain disruptions (Zhao et al. 2020, Jin 2025), or public perception of environmental performance (Du 2015, Chen and Duan 2023). At the same time, companies’ activities may have broader environmental and societal impacts, even if no direct financial risk to the business arises (Chaudhary and Kastner 2016, Yang et al. 2021). Assessing both how sustainability issues affect the company and how the company’s activities impact the environment and society more broadly is referred to as “double materiality” (European Union 2023), ensuring that both internal (financial) and external (environmental and social) consequences are considered. Governments have introduced policies to help mitigate environmental degradation driven by companies at a societal level (Bryan et al. 2018), yet major challenges persist, highlighting the need for stronger industry action through initiatives like green financing, sustainability reporting and disclosure (Shen et al. 2023, Zhang et al. 2024).

In this perspective, we examine the concept and emergence of nature-related risk and subsequent disclosure in the context of the corporate sector in China. We review potential barriers and opportunities of nature-related risk assessment frameworks like the “LEAP” framework, as recommended by the TNFD. We then recommend ways in which nature-related risk assessment and disclosures can be streamlined within the corporate sector in China.

## Potential nature-related risk exposure of companies in China

Sustainability reporting and environmental disclosures within the corporate sector in China show promising signs (Shen et al. 2023, Zhang et al. 2024). In 2021, the Ministry of Ecology and Environment in China (Ministry of Ecology and Environment 2021a) strengthened environmental reporting requirements for major polluters. Additionally, in 2022 the China Enterprise Reform and Development Society (CERDS) released its “*Guidance for Enterprise ESG Disclosures*” (China Enterprise Reform and Development Research Association 2022), which is a voluntary framework that draws selectively on international Environmental, Social, and Governance (ESG) standards while retaining a strong domestic policy orientation. Although not fully aligned with TNFD, these guidelines incorporate core elements such as materiality assessment, value-chain considerations, and disclosure of environmental impacts, providing an early foundation for integrating the TNFD LEAP framework into China’s reporting landscape. These developments signal increasing recognition of nature-related risks, yet operational adoption remains limited.

Despite broad acknowledgement of the importance of nature-related risk, initial uptake of the TNFD framework was modest in China. Of the 5346 listed companies on the Chinese Stock Exchanges as of the end of 2023, 15 companies in total are participating as TNFD as “adopters” with 2 of these being “early adopters” between 2025 and 2026 (TNFD 2023b). In contrast, Japan has the highest number of early adopter companies with 80 and 210 total adopters, followed by the United Kingdom with 46 early adopters and 92 adopters in total (TNFD 2023b). Nature-related risk disclosures present a tool for the corporate sector to ensure the future economic and environmental sustainability of Chinese companies and provide a step towards safeguarding natural capital and ecosystem services. However, because disclosure and monitoring are largely voluntary at this stage, the quality of nature-related risk assessments may vary considerably and currently depends on factors such as regulatory reviews, investor and shareholder pressure, and voluntary third-party assurances.

In 2024, the Chinese Stock Exchanges announced the adoption of nature-related risk disclosures and sustainability reporting for the largest listed companies in China. Mandatory requirements apply only to companies in the SSE 180, Shenzhen 100, STAR 50 and ChiNext indices, and those listed both domestically and abroad, rather than to all large companies in China. These indices represent the top companies within the respective stock exchange based on market capitalisation, or are thematic funds (i.e., ChiNext Index), but they exclude many listed companies and almost all small- and medium-sized enterprises, as well as many high-impact sectors such as agriculture, construction subcontractors, and upstream manufacturing (table 1).

**Table 1.** Overview of major Chinese stock indices: key characteristics and focus areas of SSE 180, Shenzhen 100, STAR 50, and ChiNext.

Full name	Stock exchange	Number	Theme	Focus
Shanghai Stock Exchange 180 Index (SSE 180 Index)	Shanghai Stock Exchange	180	Tracks the performance of the top 180 large-cap companies listed on the Shanghai Stock Exchange	Provides a benchmark for large-cap stocks and reflects the performance of leading companies in Shanghai’s A-share market
Shenzhen Stock Exchange 100 Index (Shenzhen 100)	Shenzhen Stock Exchange	100	Tracks the top 100 companies listed on the Shenzhen Stock Exchange, covering a mix of sectors	Provides a benchmark for the Shenzhen market’s performance, highlighting innovative and fast-growing companies
STAR Market 50 Index (STAR 50)	Shanghai Stock Exchange	50	Focuses on the top 50 companies in the STAR Market, which is China’s technology innovation board similar to the NASDAQ	Represents the performance of high-tech and strategically emerging industries in China, particularly in technology, biotech, and high-growth sectors
ChiNext Price Index (ChiNext Index)	Shenzhen Stock Exchange	Varies but typically includes 100+	Tracks companies listed on the ChiNext Board, which is focused on innovation, particularly in high-tech and start-up companies	Reflects the performance of small- and medium-sized innovative companies in sectors like technology, healthcare, and consumer goods

Recent developments indicate that nature-related disclosure is rapidly progressing from concept to practice. In January 2025, the Bank of China became the first Chinese financial institution to join the TNFD, marking a significant step toward mainstreaming nature-related disclosure (Taskforce on Nature-related Financial Disclosures 2025a). TNFD’s 2025 Status Report further shows that Chinese companies are among the most active globally, contributing 9.2% of all nature-related disclosures (Taskforce on Nature-related Financial Disclosures 2025b). Together, these shifts suggest a transition from exploratory early engagement toward more established and routine disclosure practices in China. Despite these advances, operational adoption of nature-related risk assessment frameworks, such as TNFD’s LEAP, remains in its early stages.

**Figure 1.**
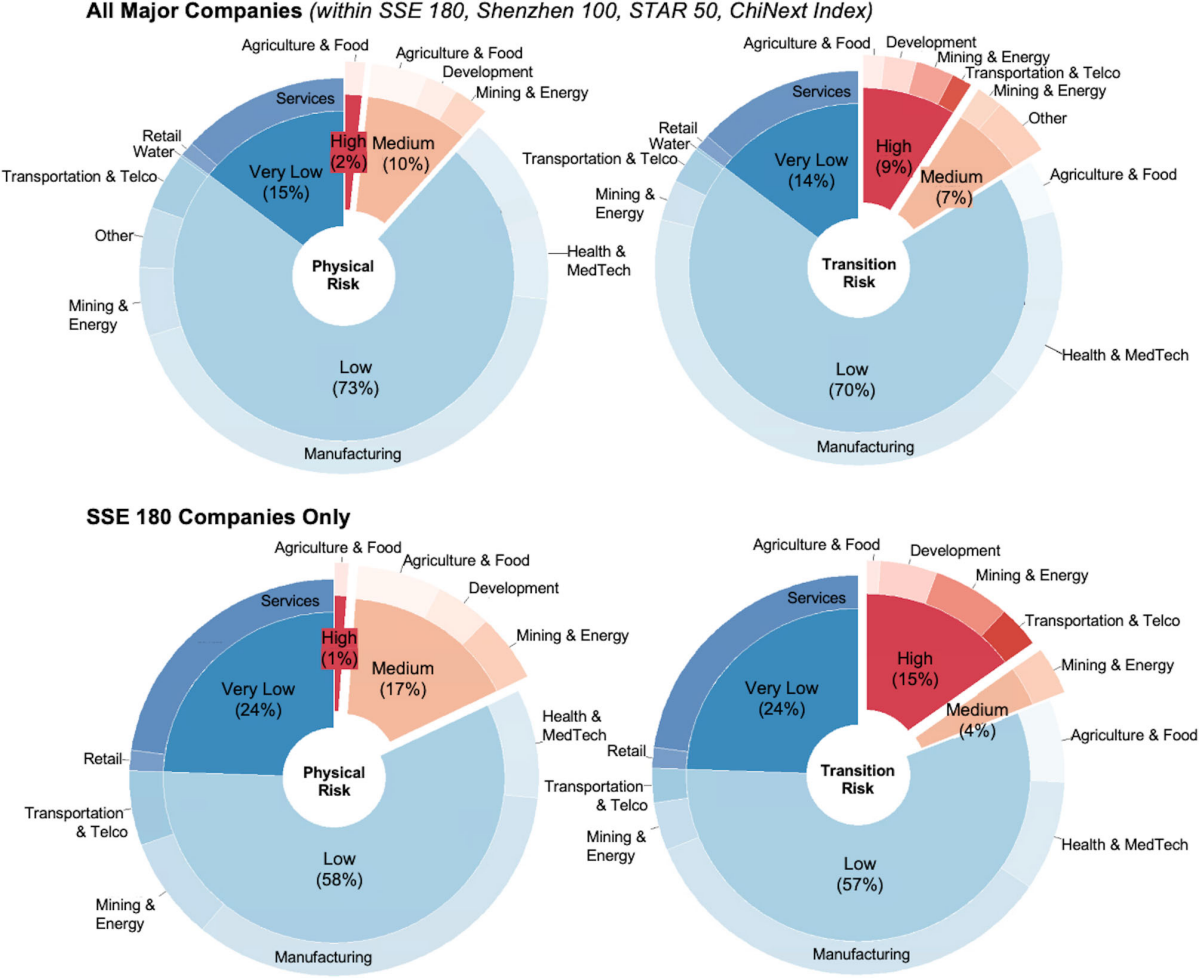
Dependency and impact summary of major listed companies in China based on WWF Biodiversity Risk Filter Suite (WWF 2023) industry sectors and direct dependencies and impacts. Physical risk weightings are derived from ENCORE’s natural capital risk ratings (https://encore.naturalcapital.finance/en). Transition risk weighting is primarily based on Science-Based Targets for Nature’s draft materiality matrix. The Biodiversity Risk Filter sector weightings only capture direct potential dependencies and impacts, not up or downstream dependencies and impacts.

We conducted a macro-level analysis to benchmark the nature-related risk exposure of each of these index funds. We accessed company holdings data within the SSE 180, Shenzhen 100, STAR 50, and ChiNext Index and nature-related risk exposure data from the World Wildlife Fund’s (WWF) Biodiversity Risk Filter Suite (WWF 2023). The WWF Biodiversity Risk Filter compiles data on the potential direct dependencies and impacts of business activities, and also summarises this for each industry sector. Companies were related to a sector based on how they have been coded in their respective index and how this aligns with the WWF Biodiversity Risk Filter Suite industries. We then summarised this information to assess the potential exposure of each index to nature-related risk based on their sector (figure 1).

We found that across all large companies within these indexes, 12% of companies are directly exposed to high or medium physical risk, and 16% are directly exposed to high or medium transition risk based on the potential dependencies and impacts of the sector. Companies within the SSE 180 are more diverse in sectors than the indexes, which are more technology-focused (i.e., ChiNext Index), thus a higher percentage of SSE 180 companies are exposed to direct nature-related physical risks based on their sector. The sectors with higher direct risks primarily fall within the agriculture and food sectors, development and construction sectors, and mining sectors. Noting that the nature-related risk within sectors can vary, and individual assessments are required to understand nature-related risk at the company level (figure 1).

Although only a subset of companies is directly affected by nature-related risks based on this assessment, this highlights the importance of indirect risks to these indices, which may stem from complex supply chains and supply chain positioning. These supply chain-related risks can be challenging to trace and manage due to the opacity of supply networks, especially when companies engage in domestic and international trade. Locating and assessing these nature-related risks may require careful implementation of recommended assessment frameworks and robust tracking of supply chain dependencies to effectively conduct nature-related risk assessments. All data analysed are included in the supplementary information files (see Supplementary Material).

## Applying the LEAP framework in China

**Figure 2.**
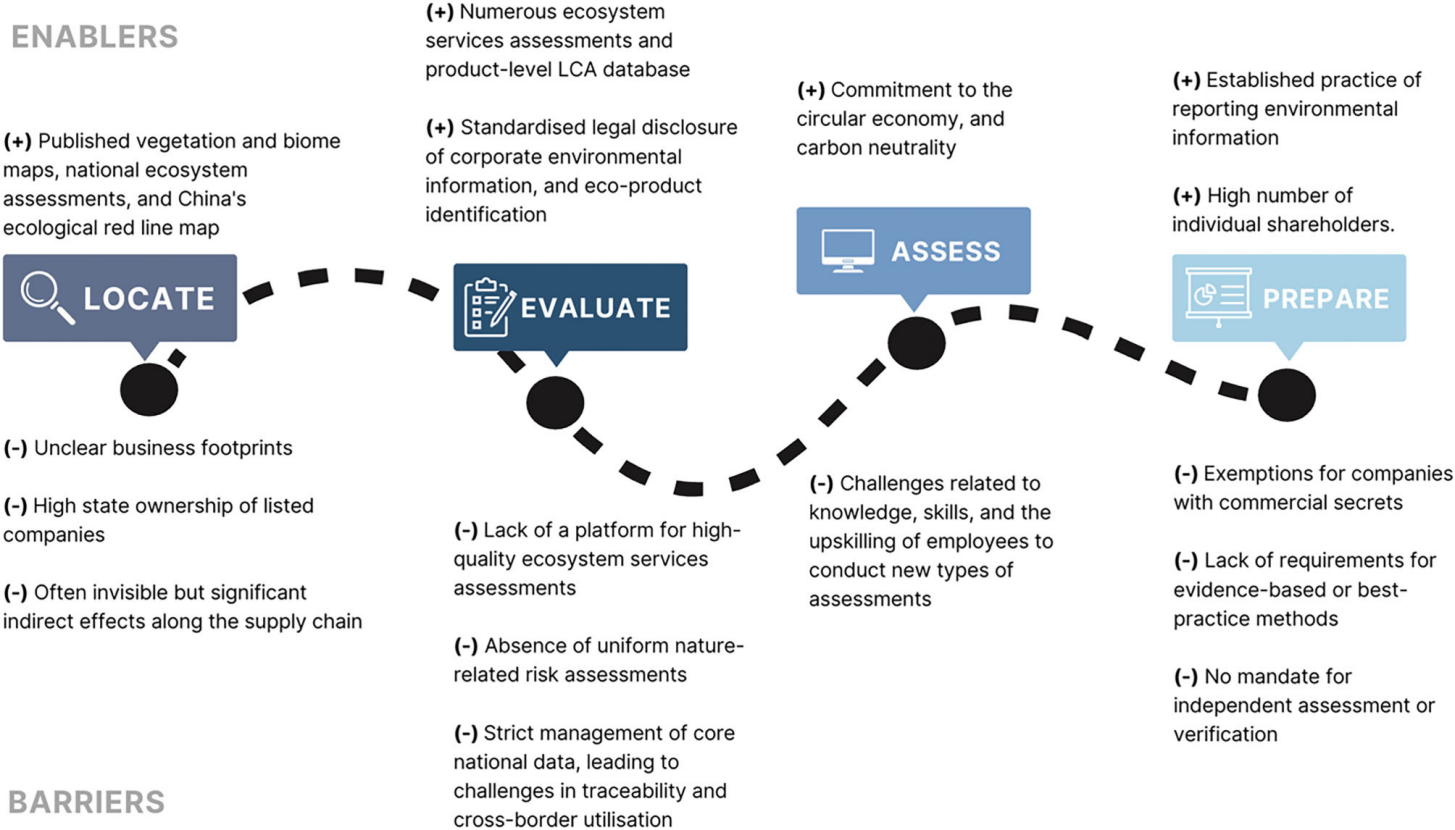
Enablers and barriers of the TNFD’s LEAP framework in China. Icons sourced from Canva under a CC0 License.

The TNFD’s “Locate, Evaluate, Assess and Prepare” (LEAP) framework was developed and is now recommended by the TNFD to use when conducting nature-related risk assessments (TNFD 2023a). This assessment provides general guidance on how to “locate” a company’s interface with nature, “evaluate” nature-related dependencies and impacts, “assess” nature-related risks and “prepare” and report on nature-related risks. However, since nature-related risk assessments and disclosures are still being adopted, businesses may face challenges when applying the LEAP guidelines due to issues like data availability, data quality, and supply chain traceability. Although similar obstacles are also prevalent in China, there are several additional enablers and barriers of LEAP assessments in the Chinese context (figure 2).

### Locate

The Locate phase determines where a business overlaps and interacts with nature. China benefits from several published datasets on vegetation and ecosystems that are already available and can be used to locate a company’s interface with nature (Tan et al. 2017, Chen et al. 2020, Meng et al. 2020, Su et al. 2020). Additionally, China’s ecological red line map (People’s Republic of China Ministry of Environmental Protection (MEP) 2015, Bai et al. 2016) and information on sectors consuming significant environmental resources enhance understanding of environmental interactions (Jin et al. 2020). However, important barriers remain, including the limited public access to spatial data on business footprints, especially related to supply chains, and the high percentage of state ownership in listed companies complicates efforts to map areas of impact.

### Evaluate

The Evaluate phase focuses on a company’s dependencies and impacts on nature. Enablers of the Evaluate phase include numerous ecosystem services assessments across China (Jiang et al. 2021), China’s product-level Life Cycle Assessment (LCA) database (Sichuan University China and IKE Environmental Technology CO Ltd China 2011), along with established eco-product identification (e.g., China Environmental Labelling (Zhao and Xia 1999)), which provide valuable information on business inputs and outputs, which can help to identify nature-related dependencies and impacts. Additionally, emerging artificial intelligence-driven initiatives like the open-source LCA database developed by the TianGong Initiative show an appetite to improve environmental and ecological data availability and quality in the region (TianGong Initiative 2025). In contrast, the absence of a unified platform for high-quality biodiversity and ecosystem services assessments (e.g., Conservation International Colombia 2015, Republic of South Africa: Department of Forestry, Fisheries and the Environment 2021) and the lack of uniform assessment hinder comparison and cross-border use, alongside strict data management laws, supply chain traceability issues, indirect dependencies, and the complex mix of socio-economic and environmental impacts. Additionally, private corporations operating on state-owned land must obtain land use rights from the government for natural capital or ecosystem services, adding another layer of complexity to managing nature-related risk.

### Assess

The Assess phase is related to the risks and opportunities that arise from the dependencies and impacts identified in the Evaluate phase. The Assess phase is supported by various national commitments to ecological civilization, which is China’s overarching policy vision that integrates social, economic, and environmental goals to achieve sustainable development, as well as circular economy principles (e.g., China’s 13th Five-Year Plan) and carbon neutrality goals (e.g., carbon neutrality by 2060 and peak carbon emissions by 2030) (Central Committee of the Communist Party of China (CPC) 2020, Liu et al. 2021, Bleischwitz et al. 2022). For example, China’s Ecological Redline Policy also shapes risk assessment by restricting development in ecologically sensitive zones, creating potential regulatory transition risks for sectors with land-intensive footprints. However, effective risk management can be resource-intensive and challenging, especially when motivated by the need for evidence-based and independently verified approaches to avoid potential greenwashing (Du 2015), along with growing demand for specialists in nature-related risk analysis, which may exceed the existing capacity.

### Prepare

The Prepare phase is related to how a company will respond to the nature-related risks identified. The Prepare phase is enabled by the established practice of reporting and disclosing environmental information through the “*Legal Disclosure Report of Environmental Information*” (Ministry of Ecology and Environment 2021b) and the “*Enterprise Environmental Information Legal Disclosure System*” for major market enterprises. However, exemptions for companies claiming commercial confidentiality can restrict full transparency and comprehensive risk disclosure. Distinct challenges also exist for different enterprise types in China. For example, state-owned enterprises face additional administrative and reporting burdens and often operate within more complex governance structures, which can hinder comprehensive nature-related risk disclosure (He et al. 2019, Zhang 2024).

## Navigating nature-related risks across China’s global trade networks

China consistently ranks as one of the top global exporters and importers across multiple sectors (International Trade Centre 2023, World Bank 2023), making it a key player in global trade. Yet, a significant challenge with nature-related risk assessments is assessing upstream and downstream dependencies and impacts related to a business’s value chain. These indirect dependencies can be divided into two aspects. The first aspect involves the supply chain, focusing on the upstream and downstream relationships among firms. This is especially crucial for China’s largest listed companies, given their substantial role in manufacturing and services within the global economy (figure 1). For example, carbon footprint modelling across multinational supply chains shows that although emissions intensity per unit of output has decreased in China, reflecting the role of improved supply chain efficiency, the total carbon footprint continues to rise due to growing final demand for goods and services (Acquaye et al. 2018). This divergence illustrates how difficult it is to evaluate upstream and downstream impacts within value chains (Zhao 2024), reinforcing the need for nature-related risk assessments in China to account for the full breadth of consumption-driven dependencies.

Chinese companies are increasingly subject to dual scrutiny, both domestically, through national environmental regulations, and internationally, through sustainability expectations in global markets. This dual scrutiny can arise when primary products are sourced internationally from regions with high conservation or ecological importance, while final products are sold abroad. These trade-related dependencies mean that firms may be exposed to additional nature-related risks, such as deforestation, soil erosion, water pollution from upstream suppliers, or regulatory action in export markets (Wang et al. 2021). For companies to disclose their nature-related risks transparently, it is essential to consider these trade-related dependencies and impacts comprehensively. New datasets and improved footprinting and risk assessment methods now make it more feasible to quantify these indirect supply chain impacts with greater accuracy and spatial resolution (Cabernard and Pfister 2021, Schrapffer et al. 2024).

China’s environmental regulations, while strengthening, remain still less stringent and less consistently enforced than those of many developed countries. This regulatory gap has led to a great deal of environmental problems arising from China’s exports and the scrutiny of its exports by foreign consumers. For example, despite China’s ban on the export of mercury-containing products due to the enforcement of the Minamata Convention on Mercury (Minamata Convention on Mercury 2023), mercury is still used in large quantities in the upstream supply chain for several industrial products, and there is a high uncertainty and variance in mercury footprints across China (Zhang et al. 2019), posing potentially hidden nature-related risks.

Government-mandated disclosure of natural risks may be scrutinised by buyers and may influence product desirability in a similar way to climate-related disclosures (Liesen et al. 2017) or credibility and brand image (García‐Sánchez et al. 2022) of products and services to investors or customers. To satisfy these potential expectations, large listed companies should conduct thorough assessments guided by the TNFD to assess and disclose their practices, perhaps in a similar way as industry-related air pollution (Zhang et al. 2017). With increasing global scrutiny of corporate environmental practices, Chinese companies must stay abreast of evolving international regulations to avoid penalties or market access restrictions due to non-compliance. However, there could be discrepancies in what companies share with domestic and foreign investors (Du 2018).

In transnational supply chains, the completeness and accuracy of nature-related risk assessments can be hindered by insufficient or inconsistent disclosure of information from suppliers. Chinese companies often rely on complex, multi-tiered supply chains, making it challenging to track environmental information accurately (Zhao 2024). This opacity makes it difficult to accurately assess a company’s true environmental impact, as undisclosed information from upstream suppliers can mask significant risks (Chen and Duan 2023) (figure 2). One way to overcome this challenge is for companies to invest in advanced data collection and analysis tools to enhance supply chain transparency for their risk assessments, enabling effective risk management and decision-making. However, even with improved data, mandatory disclosure only covers companies in major indices, leaving many biodiversity-relevant activities under-reported. Voluntary disclosure by non-listed companies, especially those in land-use-intensive sectors or upstream suppliers, remains critical to capture risks where ecological impacts are most concentrated.

China also plays a significant role as an importer, particularly of certain food commodities (e.g., soybeans, maize, beef, and dairy) with high impact on biodiversity (Chaudhary and Kastner 2016), but also other materials such as minerals, cotton, wool, and timber originating from areas where the potential risk to nature is significant. For example, China imports a substantial amount of soybeans from Brazil, which can lead to significant impacts due to land use change and degradation in highly biodiverse areas, but is also impacted by factors like climate change, which contributes towards nature-related risks (Ali et al. 2022). This will mean that many companies that rely on imports may be required to report on potential indirect impacts such as deforestation or pollution, which can impact transition and biodiversity risks (Schrapffer et al. 2024). The European Union’s recent Regulation on Deforestation Free Products (EUDR) could be used as a template for China and could be used to also inform bilateral trade relationships (Kehoe et al. 2020).

China’s imports and exports affect not only the environment of China and its trade partners but can also have spillover effects for non-trade partners (e.g., CO_2_ emissions during manufacturing and transporting goods for trade partners that affect the global climate) (Liu et al. 2018, Wang et al. 2024). On the other hand, non-trade partners also have spillover effects on China. For example, soybean production in Brazil for China depends on fertilisers from a number of countries such as Canada, Germany, and Russia that export no or little soybeans to China. Reduction in exports of fertilizers in those countries to Brazil affects Brazil’s soybean production and exports to China (Liu et al. 2018). International conflicts such as the Russia-Ukraine war may have enormous impacts on global trade networks, cropland expansion, and biodiversity, with cascading effects on China’s businesses (Chai et al. 2024). Understanding these shifted or telecoupled impacts will be particularly important under various policies such as the Conference of the Parties (COP) “*Glasgow Leaders' Declaration on Forests and Land Use*” in which China is a signatory, or company commitments that strive for zero deforestation supply chains (UKCOP26 2021).

## Turning nature-related risks into opportunities for sustainable business in China

Despite the challenges in nature-related risk disclosure in China, there are significant opportunities to address these barriers at each stage of the TNFD’s LEAP framework for nature-related risk assessment (figure 2). To improve how businesses address their relationship with nature in China, several actions are needed across different areas.

Firstly, enhancing the transparency and traceability of supply chains is essential to better understand their complex relationships with the environment. By quantifying these relationships and identifying vulnerable points (Zhao et al. 2020), businesses can manage ecological tipping points, minimising nature-related risks (Rockström et al. 2009, 2023, Richardson et al. 2023). Recent initiatives, such as traceable procurement and technological innovations, can further support risk assessments by improving supply chain transparency (Steinhäuser et al. 2022), thereby mitigating risks like resource depletion and biodiversity loss while building consumer trust. This is especially relevant given that mandatory disclosure only covers companies in major indices, leaving many large but non-listed companies unreported. Encouraging voluntary disclosure can enhance traceability and capture risks in regions of high ecological impact.

Secondly, structured reporting requirements are often used as a lever for environmental change, yet overly rigid corporate social responsibility reporting can create unintended consequences, shaping company behaviour in ways that may be misaligned with investor expectations or broader societal goals. Improving information availability and applying a consistent process, such as the LEAP framework, while allowing flexible implementation and verification, can help businesses more effectively evaluate their dependencies and impacts on nature (Christensen et al. 2021). Improving the underlying data for these assessments by developing a comprehensive ecosystem-services platform would further support companies by consolidating existing datasets and allowing for context-specific risk assessments. Expanding access to spatial information, including for state-owned enterprises, is also critical, as high levels of state ownership in China can obscure operational assets and land-use impacts, reducing transparency and limiting effective risk mapping.

Finally, considering trade relationships and realigning employee skill sets are vital for assessing nature-related risks. Metacoupling analysis could help evaluate the impacts of these risks within China and globally (Liu 2023). Aligning skills in ecosystem services assessments to nature-related risk assessments could also bolster the sustainability sector. Finally, to prepare for reporting and disclosures, businesses should adopt stricter evidence-based assessment methods and require independent verifications to ensure high standards if a less standardised approach to assessment is adopted.

Given the increasing importance of China’s corporate sector in shaping global sustainability (Gao 2024), China’s government and financial regulators hold significant influence in fostering sustainability. Yet, mandatory nature-related risk disclosures alone do not capture all ecological risks as many large, non-listed companies are excluded from indices. Encouraging voluntary reporting for these companies can help fill critical information gaps. Publicly disclosing nature-related risks allows companies to demonstrate their commitment to environmental protection and long-term sustainability (Clarkson et al. 2008). This transparency enhances their image and reputation among both domestic and international stakeholders, including investors. However, mandatory reporting can sometimes lead to unintended behavioural consequences (Christensen et al. 2021). There is also mixed evidence around whether sustainability commitments lead to positive outcomes for business in China (Du 2015), there may also be differences in how sustainability is communicated between domestic and foreign markets (Du 2015, 2018).

Implementing smart governance that balances nature and financial capital, while fostering partnerships, cost-sharing, and knowledge-sharing, can lead to innovative solutions benefiting both nature and business (Giuliodori et al. 2023), thus turning potential risks into shared opportunities for growth and innovation. Clearly defining responsibility for assessing nature-related impacts, including for state-owned or non-listed enterprises, can drive continuous improvement in reporting and risk management (Du 2018, He et al. 2019, Ren and Ren 2024). Particularly as eco-civilisation is a cornerstone of China’s national strategy, this presents an ideal opportunity for all stakeholders to engage in this “last mile” action (Jiang et al. 2019).

Beyond responding to nature-related risks through disclosures, several actions can enhance the likelihood that such disclosures will lead to positive impacts for nature. One key strategy is to enhance supply chain resilience by integrating cost-effective nature-based solutions, such as green spaces, wetlands, and sustainable agriculture, into project designs (Chausson et al. 2020, Seddon et al. 2020). Promoting technological innovations and solutions that align with nature-positive pathways is another recommendation. For example, investing in green building materials and fostering cooperative innovation within integrated supply chains can facilitate this transition (Xia et al. 2020). Such innovations can help businesses reduce environmental impacts, explore emerging markets driven by the transition to a sustainable economy, and improve long-term profitability. This might include expanding into industries like renewable energy, sustainable agriculture, eco-friendly products, or green finance, where nature-related risks become opportunities for innovation and growth.

Finally, increasing shareholder and public awareness of nature-related risks and encouraging their participation in monitoring and reporting environmentally harmful behaviours is crucial. Both news and social media can play a significant role in quickly disseminating relevant information, increasing transparency, and reducing risks (Cho et al. 2025). Furthermore, businesses should proactively educate employees, customers, and stakeholders on the importance of sustainability and their roles in managing nature-related risks. By fostering a culture of sustainability within the organisation and among partners, businesses can drive collective action towards nature-positive outcomes and gain a competitive advantage.

## Conclusion

Growing global attention to sustainability issues enables companies to prioritise social responsibility and adopt sustainable practices (Gao 2024). Although many large-listed companies in China are not directly affected by nature-related risks according to this assessment, their supply chain positions and complex trade relationships make it challenging to assess indirect risks. Challenges such as limited supply chain transparency, dependencies, and environmental impacts all contribute to this complexity.

To further enable nature-related risk assessment and disclosures in China, it is important to address key barriers such as the absence of assessment platforms and uniform methods, publicly governed natural capital rights, strict data management rules, insufficient supply chain traceability, and complex dependencies and impacts influenced by supply chain positioning and influence in China and beyond (Chen et al. 2021, Lin et al. 2023).

By addressing these challenges, China’s corporate sector is well-positioned not only to advance national sustainability objectives but also to enhance its global competitiveness. Strengthened and systematised nature-related risk disclosures can help Chinese companies demonstrate leadership, meet emerging international expectations, and set a new standard for sustainable business practices in a rapidly evolving global market.

## Supplementary Material

biag071_Supplemental_Files
