# Supplementary material for: Towards nature-related risk disclosures in China
Source: Bioscience. 2026 Jun 18;76(7):591–600. doi: 10.1093/biosci/biag071 (PMC13377638; doi:10.1093/biosci/biag071)
Supplement: biag071_Supplemental_Files [file biag071_supplemental_files.zip › Supp_Data.pdf]

| | Company Name | Code |
|---|---|---|
| **Index** | | |
| ChiNex Index (CNXT) | Wens Foodstuffs Group Co Ltd | BBG00F13F476 |
| Shenzhen 100 | Muyuan | 2714 |
| Shenzhen 100 | Wens | 300498 |
| Shenzhen 100 | Shuanghui | 895 |
| SSE 180 | INNER MONGOLIA YILI INDUSTRIAL GRO | 600887 |
| Shenzhen 100 | New Hope | 876 |
| SSE 180 | TONGWEI CO.,LTD | 600438 |
| ChiNex Index (CNXT) | Centre Testing International Group Co L | BBG00F138RK8 |
| ChiNex Index (CNXT) | Shandong Sinocera Functional Material ( | BBG00FNDHL98 |
| Shenzhen 100 | Midea Group | 333 |
| Shenzhen 100 | Gree | 651 |
| Shenzhen 100 | Sanhua | 2050 |
| STAR 50 | Beijing Roborock Technology Co ., Ltd. | 688169 |
| SSE 180 | Ecovacs Robotics Co., Ltd. | 603486 |
| SSE 180 | Haier Smart Home Co., Ltd. | 600690 |
| SSE 180 | Ningbo Deye Technology Co., Ltd. | 605117 |
| ChiNex Index (CNXT) | Dongguan Yiheda Automation Co Ltd | BBG017ZPHSQ1 |
| ChiNex Index (CNXT) | Eve Energy Co Ltd | BBG00F138RN5 |
| ChiNex Index (CNXT) | Gaona Aero Material Co Ltd | BBG00F138V38 |
| ChiNex Index (CNXT) | Leyard Optoelectronic Co Ltd | BBG00F13D7Y1 |
| ChiNex Index (CNXT) | Qingdao Tgood Electric Co Ltd | BBG00F138PD0 |
| ChiNex Index (CNXT) | Sg Micro Corp | BBG00MYKWCC |
| ChiNex Index (CNXT) | Wuhan Dr Laser Technology Corp Ltd | BBG00VC80K69 |
| ChiNex Index (CNXT) | Xi'An Triangle Defense Co Ltd | BBG00VC80JX2 |
| ChiNex Index (CNXT) | Yangling Metron New Material Inc | BBG011C97GP3 |
| Shenzhen 100 | BYD | 2594 |
| Shenzhen 100 | Changan Automobile | 625 |
| Shenzhen 100 | Weichai Power | 338 |
| Shenzhen 100 | AVIC XAC | 768 |
| Shenzhen 100 | EVE | 300014 |
| STAR 50 | AVIC (CHENGDU) UAS CO., LTD. | 688297 |
| STAR 50 | Shanghai Friendess Electronic Technolog | 688188 |
| STAR 50 | Zhuzhou CRRC Times Electric Co., Ltd. | 688187 |
| SSE 180 | AECC AVIATION POWER CO,LTD | 600893 |
| SSE 180 | Anhui Jianghuai Automobile Group Corp. | 600418 |
| SSE 180 | AVIC HEAVY MACHINERY CO.,LTD. | 600765 |
| SSE 180 | AVIC SHENYANG AIRCRAFT COMPANY LI | 600760 |
| SSE 180 | Bethel Auto Safety Systems Co.,Ltd | 603596 |
| SSE 180 | Great Wall Motor Company Limited | 601633 |
| SSE 180 | GUANGZHOU AUTOMOBILE GROUP CO., | 601238 |
| SSE 180 | SAIC Motor Corporation Limited | 600104 |
| SSE 180 | China Shipbuilding Industry Company Lin | 601989 |
| SSE 180 | China Spacesat Co.,Ltd. | 600118 |
| SSE 180 | CRRC Corporation Limited | 601766 |
| SSE 180 | DONGFANG ELECTRIC CORPORATION LI | 600875 |
| SSE 180 | FUYAO GLASS INDUSTRY GROUP CO., LT | 600660 |
| SSE 180 | HOYUAN Green Energy Co.,Ltd | 603185 |
| SSE 180 | jiangsu hengli hydraulic co.Ltd | 601100 |
| SSE 180 | Ming Yang Smart Energy Group Limited | 601615 |
| SSE 180 | Ningbo Tuopu Group Co.,Ltd. | 601689 |
| SSE 180 | SANY HEAVY INDUSTRY CO.,LTD | 600031 |
| SSE 180 | TBEA CO.,LTD. | 600089 |
| SSE 180 | Wingtech Technology Co.,Ltd. | 600745 |
| SSE 180 | ZHEJIANG CHINT ELECTRICS CO.,LTD. | 601877 |

| | | |
|---|---|---|
| SSE 180 | ZHEJIANG HUAYOU COBALT CO., LTD. | 603799 |
| ChiNex Index (CNXT) | Hubei Feilihua Quartz Glass Co Ltd | BBG00VC817P6 |
| ChiNex Index (CNXT) | Jl Mag Rare-Earth Co Ltd | BBG00PF5F1K5 |
| ChiNex Index (CNXT) | Konfoong Materials International Co Ltd | BBG00JN03482 |
| ChiNex Index (CNXT) | Shandong Weifang Rainbow Chemical Co | BBG017ZPJGH5 |
| ChiNex Index (CNXT) | Shenzhen Capchem Technology Co Ltd | BBG00F138V83 |
| ChiNex Index (CNXT) | Sunresin New Materials Co Ltd | BBG00MYKW7J8 |
| ChiNex Index (CNXT) | Weihai Guangwei Composites Co Ltd | BBG00LBJGFK6 |
| Shenzhen 100 | Tinci | 2709 |
| Shenzhen 100 | RSPC | 2493 |
| STAR 50 | Xinjiang Daqo New Energy Co.,Ltd | 688303 |
| STAR 50 | Zhongfu Shenying Carbon Fiber Co., Ltd | 688295 |
| SSE 180 | Xinjiang Daqo New Energy Co.,Ltd. | 688303 |
| SSE 180 | JIANGSU PACIFIC QUARTZ CO.,LTD. | 603688 |
| SSE 180 | NINGBO ORIENT WIRES & CABLES CO., LT | 603606 |
| SSE 180 | SHANDONG HUALU-HENGSHENG CHEMI | 600426 |
| SSE 180 | Wanhua Chemical Group Co.,Ltd. | 600309 |
| SSE 180 | China Jushi Co.,Ltd. | 600176 |
| SSE 180 | China National Chemical Engineering CO. | 601117 |
| SSE 180 | China Northern Rare Earth (Group) High- | 600111 |
| SSE 180 | Hoshine Silicon Industry Co., Ltd. | 603260 |
| SSE 180 | Jinduicheng Molybdenum Co.,Ltd | 601958 |
| SSE 180 | YUNNAN YUNTIANHUA CO.,LTD | 600096 |
| SSE 180 | ZHEJIANG JUHUA CO.,LTD. | 600160 |
| Shenzhen 100 | Zoomlion | 157 |
| Shenzhen 100 | XCMG | 425 |
| Shenzhen 100 | Oriental Yuhong Waterproof Technology | 2271 |
| Shenzhen 100 | CITIC Steel | 708 |
| SSE 180 | Anhui Conch Cement Company Limited | 600585 |
| SSE 180 | Baoshan Iron & Steel Co., Ltd. | 600019 |
| SSE 180 | Inner Mongolia BaoTou Steel Union Co.,I | 600010 |
| SSE 180 | Oppein Home Group Inc. | 603833 |
| Shenzhen 100 | ENERGY TECHNOLOGY | 2812 |
| Shenzhen 100 | CGN | 3816 |
| SSE 180 | China National Nuclear Power Co., Ltd. | 601985 |
| SSE 180 | China Shenhua Energy Company Limited | 601088 |
| SSE 180 | CNOOC Limited | 600938 |
| SSE 180 | Huadian Power International Corporatio | 600027 |
| SSE 180 | Huaneng Power International, INC. | 600011 |
| SSE 180 | SHAANXI COAL INDUSTRY COMPANY LIM | 601225 |
| SSE 180 | Yankuang Energy Group Company Limite | 600188 |
| ChiNex Index (CNXT) | Risen Energy Co Ltd | BBG00F139TS5 |
| ChiNex Index (CNXT) | Shenzhen Sc New Energy Technology Co | BBG00PF5F036 |
| ChiNex Index (CNXT) | Sungrow Power Supply Co Ltd | BBG00F13CY28 |
| Shenzhen 100 | Sungrow Power Supply | 300274 |
| Shenzhen 100 | JA Solar | 2459 |
| Shenzhen 100 | Goldwind | 2202 |
| STAR 50 | Sany Heavy Energy Co., Ltd. | 688349 |
| SSE 180 | China Energy Engineering Corporation Li | 601868 |
| SSE 180 | China Three Gorges Renewables (Group) | 600905 |
| SSE 180 | LONGi Green Energy Technology Co., Ltd | 601012 |
| SSE 180 | Shanghai Aiko Solar Energy Co., Ltd. | 600732 |
| SSE 180 | GD POWER DEVELOPMENT CO., LTD | 600795 |
| SSE 180 | Power Construction Corporation of China | 601669 |
| SSE 180 | SHANGHAI ELECTRIC POWER COMPANY | 600021 |
| SSE 180 | China Yangtze Power Co.,ltd. | 600900 |
| ChiNex Index (CNXT) | Anker Innovations Technology Co Ltd | BBG011C97H08 |
| ChiNex Index (CNXT) | Beijing Easpring Material Technology Co | BBG00FNDHC25 |
| ChiNex Index (CNXT) | Canmax Technologies Co Ltd | BBG00YG5D7D2 |

| | | |
|---|---|---|
| ChiNex Index (CNXT) | Chaozhou Three-Circle Group Co Ltd | BBG00F13DSS2 |
| ChiNex Index (CNXT) | Cngr Advanced Material Co Ltd | BBG013Y510B9 |
| ChiNex Index (CNXT) | Contemporary Amperex Technology Co L | BBG00MYKWDB3 |
| ChiNex Index (CNXT) | East Group Co Ltd | BBG00F13DS48 |
| ChiNex Index (CNXT) | Empyrean Technology Co Ltd | BBG01FRBBM91 |
| ChiNex Index (CNXT) | Ginlong Technologies Co Ltd | BBG00YG5D9P5 |
| ChiNex Index (CNXT) | Guangzhou Great Power Energy & Techn | BBG00FNDHRG7 |
| ChiNex Index (CNXT) | Hangzhou Chang Chuan Technology Co L | BBG00VC81FX9 |
| ChiNex Index (CNXT) | Hubei Dinglong Co Ltd | BBG00F138W72 |
| ChiNex Index (CNXT) | Hunan Yuneng New Energy Battery Mate | BBG01G92FS65 |
| ChiNex Index (CNXT) | Ingenic Semiconductor Co Ltd | BBG00FNDHJJ2 |
| ChiNex Index (CNXT) | Lens Technology Co Ltd | BBG00F13DT73 |
| ChiNex Index (CNXT) | Maxscend Microelectronics Co Ltd | BBG00VC80L30 |
| ChiNex Index (CNXT) | Semitronix Corp | BBG01FR9T987 |
| ChiNex Index (CNXT) | Shenzhen Dynanonic Co Ltd | BBG013Y4ZS51 |
| ChiNex Index (CNXT) | Shenzhen Inovance Technology Co Ltd | BBG00F139TZ7 |
| ChiNex Index (CNXT) | Shenzhen Longsys Electronics Co Ltd | BBG01FRBBRP2 |
| ChiNex Index (CNXT) | Shenzhen Senior Technology Material Co | BBG00MYKW8B4 |
| ChiNex Index (CNXT) | Shenzhen Sunway Communication Co Lt | BBG00F139VD6 |
| ChiNex Index (CNXT) | Sunwoda Electronic Co Ltd | BBG00F13BZ08 |
| ChiNex Index (CNXT) | Suzhou Maxwell Technologies Co Ltd | BBG00R2FQVG0 |
| ChiNex Index (CNXT) | Wuhan Jingce Electronic Group Co Ltd | BBG00LBJC7L7 |
| ChiNex Index (CNXT) | Wuxi Lead Intelligent Equipment Co Ltd | BBG00H1LL778 |
| ChiNex Index (CNXT) | Yangzhou Yangjie Electronic Technology | BBG00F13DS20 |
| ChiNex Index (CNXT) | Zhejiang Jingsheng Mechanical & Electri | BBG00F13DKX3 |
| ChiNex Index (CNXT) | Zhuzhou Hongda Electronics Corp Ltd | BBG00LBJGMF7 |
| Shenzhen 100 | CATL | 300750 |
| Shenzhen 100 | Inovance | 300124 |
| Shenzhen 100 | Lead Intelligent | 300450 |
| Shenzhen 100 | Luxshare-ICT | 2475 |
| Shenzhen 100 | Hikvision | 2415 |
| Shenzhen 100 | BOE | 725 |
| Shenzhen 100 | Goertek | 2241 |
| Shenzhen 100 | Guoxin Micro | 2049 |
| Shenzhen 100 | TJSEMI | 2129 |
| Shenzhen 100 | NAURA | 2371 |
| Shenzhen 100 | TCL Tech. | 100 |
| Shenzhen 100 | Maxscend | 300782 |
| Shenzhen 100 | JONHON | 2179 |
| Shenzhen 100 | CCTC | 300408 |
| Shenzhen 100 | Han's Laser | 2008 |
| Shenzhen 100 | Lens | 300433 |
| Shenzhen 100 | Dahua Inc | 2236 |
| Shenzhen 100 | LCXX | 977 |
| Shenzhen 100 | CVTE | 2841 |
| Shenzhen 100 | Avary Holding | 2938 |
| Shenzhen 100 | Guide Infrared | 2414 |
| Shenzhen 100 | Shennan Circuits | 2916 |
| STAR 50 | 3PEAK INCORPORATED | 688536 |
| STAR 50 | ACM Research (Shanghai), Inc. | 688082 |
| STAR 50 | Advanced Micro-Fabrication Equipment | 688012 |
| STAR 50 | Amlogic (Shanghai) Co., Ltd. | 688099 |
| STAR 50 | Cambricon Technologies Corporation Lin | 688256 |
| STAR 50 | China Resources Microelectronics Limite | 688396 |
| STAR 50 | Everdisplay Optronics (Shanghai) Co., Ltc | 688538 |
| STAR 50 | GalaxyCore Inc. | 688728 |
| STAR 50 | GoodWe Technologies Co.,Ltd. | 688390 |
| STAR 50 | Hoymiles Power Electronics Inc. | 688032 |
| STAR 50 | HUNAN CHANGYUAN LICO CO.,LTD. | 688779 |

| | | |
|---|---|---|
| STAR 50 | Hwatsing Technology Co.,Ltd. | 688120 |
| STAR 50 | Hygon Information Technology Co., Ltd. | 688041 |
| STAR 50 | Jinko Solar Co., Ltd. | 688223 |
| STAR 50 | Loongson Technology Corporation Limite | 688047 |
| STAR 50 | Montage Technology Co., Ltd. | 688008 |
| STAR 50 | National Silicon Industry Group Co., Ltd. | 688126 |
| STAR 50 | Ningbo Ronbay New Energy Technology | 688005 |
| STAR 50 | Piotech Inc. | 688072 |
| STAR 50 | Pylon Technologies Co., Ltd. | 688063 |
| STAR 50 | Semiconductor Manufacturing Internatio | 688981 |
| STAR 50 | Shanghai Fudan Microelectronics Group | 688385 |
| STAR 50 | SICC Co., Ltd. | 688234 |
| STAR 50 | SUPCON Technology Co., Ltd. | 688777 |
| STAR 50 | Suzhou Novosense Microelectronics Co., | 688052 |
| STAR 50 | Tianneng Battery Group Co., Ltd. | 688819 |
| STAR 50 | Transsion Holdings LTD.CO | 688036 |
| STAR 50 | Trina Solar Co., Ltd. | 688599 |
| STAR 50 | VeriSilicon Microelectronics (Shanghai) C | 688521 |
| STAR 50 | Western Superconducting Technologies ( | 688122 |
| STAR 50 | Yuneng Technology Co., Ltd. | 688348 |
| SSE 180 | Advanced Micro-Fabrication Equipment | 688012 |
| SSE 180 | Cambricon Technologies Corporation Lim | 688256 |
| SSE 180 | GigaDevice Semiconductor Inc. | 603986 |
| SSE 180 | GoodWe Technologies Co.,Ltd. | 688390 |
| SSE 180 | Hygon Information Technology Co., Ltd. | 688041 |
| SSE 180 | Jinko Solar Co., Ltd. | 688223 |
| SSE 180 | Montage Technology Co., Ltd. | 688008 |
| SSE 180 | Pylon Technologies Co., Ltd. | 688063 |
| SSE 180 | Semiconductor Manufacturing Internatio | 688981 |
| SSE 180 | STARPOWER SEMICONDUCTOR LTD. | 603290 |
| SSE 180 | Transsion Holdings LTD.CO | 688036 |
| SSE 180 | Trina Solar Co., Ltd. | 688599 |
| SSE 180 | Western Superconducting Technologies ( | 688122 |
| SSE 180 | Will Semiconductor CO., Ltd. Shanghai | 603501 |
| SSE 180 | Dawning Information Industry Co., Ltd. | 603019 |
| SSE 180 | HANGZHOU FIRST APPLIED MATERIAL CC | 603806 |
| SSE 180 | Hangzhou Lion Microelectronics Co.,Ltd. | 605358 |
| SSE 180 | Hangzhou Silan Microelectronics Co.,Ltd. | 600460 |
| SSE 180 | JCET Group Co., Ltd. | 600584 |
| SSE 180 | Shanghai Putailai New Energy Technolog | 603659 |
| ChiNex Index (CNXT) | Yihai Kerry Arawana Holdings Co Ltd | BBG00YG5DCM1 |
| Shenzhen 100 | Wuliangye | 858 |
| Shenzhen 100 | Luzhou Lao Jiao | 568 |
| Shenzhen 100 | Yanghe | 2304 |
| Shenzhen 100 | Haid Group | 2311 |
| Shenzhen 100 | Arawana | 300999 |
| Shenzhen 100 | Gujing Distillery | 596 |
| SSE 180 | JUEWEI FOOD CO., LTD. | 603517 |
| SSE 180 | ANGEL YEAST CO.,LTD | 600298 |
| SSE 180 | Anhui Kouzi Distillery Co., Ltd. | 603589 |
| SSE 180 | ANJOY FOODS GROUP CO.,LTD. | 603345 |
| SSE 180 | Chongqing Brewery Company Limited | 600132 |
| SSE 180 | Foshan Haitian Flavouring and Food Com | 603288 |
| SSE 180 | SHANXI XINGHUACUN FEN WINE FACTOI | 600809 |
| SSE 180 | TSINGTAO BREWERY COMPANY LIMITED | 600600 |
| SSE 180 | Jiangsu King's Luck Brewery Joint – Stock | 603369 |
| SSE 180 | JONJEE HI-TECH INDUSTRIAL & COMMEF | 600872 |
| SSE 180 | KWEICHOW MOUTAI CO.,LTD. | 600519 |
| SSE 180 | SICHUAN SWELLFUN CO.,LTD | 600779 |

| | | |
|---|---|---|
| Shenzhen 100 | SF Holding | 2352 |
| Shenzhen 100 | Supor | 2032 |
| SSE 180 | China Tourism Group Duty Free Corporat | 601888 |
| SSE 180 | Shanghai Jin Jiang International Hotels C | 600754 |
| SSE 180 | WANGFUJING GROUP CO., LTD. | 600859 |
| ChiNex Index (CNXT) | Aier Eye Hospital Group Co Ltd | BBG00F138RQ2 |
| ChiNex Index (CNXT) | Anhui Anke Biotechnology Group Co Ltd | BBG00F138QW7 |
| ChiNex Index (CNXT) | Betta Pharmaceuticals Co Ltd | BBG00H1LL7F9 |
| ChiNex Index (CNXT) | Bgi Genomics Co Ltd | BBG00LBJFCZ8 |
| ChiNex Index (CNXT) | By-Health Co Ltd | BBG00F13B078 |
| ChiNex Index (CNXT) | China Resources Boya Bio-Pharmaceutica | BBG00F13D708 |
| ChiNex Index (CNXT) | Chongqing Zhifei Biological Products Co | BBG00F139TV1 |
| ChiNex Index (CNXT) | Dian Diagnostics Group Co Ltd | BBG00F13CFR2 |
| ChiNex Index (CNXT) | Hangzhou Tigermed Consulting Co Ltd | BBG00F13DR59 |
| ChiNex Index (CNXT) | Hualan Biological Vaccine Inc | BBG01BWNBNP8 |
| ChiNex Index (CNXT) | Huaxia Eye Hospital Group Co Ltd | BBG01FRBBLP5 |
| ChiNex Index (CNXT) | Imeik Technology Development Co Ltd | BBG011C97SS4 |
| ChiNex Index (CNXT) | Jafron Biomedical Co Ltd | BBG00LBJC657 |
| ChiNex Index (CNXT) | Lepu Medical Technology Beijing Co Ltd | BBG00F138PZ6 |
| ChiNex Index (CNXT) | Ovctek China Inc | BBG00MYKW8V2 |
| ChiNex Index (CNXT) | Pharmaron Beijing Co Ltd | BBG00R08YHZ9 |
| ChiNex Index (CNXT) | Porton Pharma Solutions Ltd | BBG00F13DRR5 |
| ChiNex Index (CNXT) | Shenzhen Kangtai Biological Products Co | BBG00LBJC7X4 |
| ChiNex Index (CNXT) | Shenzhen Mindray Bio-Medical Electroni | BBG00MYKWDD |
| ChiNex Index (CNXT) | Shenzhen New Industries Biomedical Eng | BBG011C97D07 |
| ChiNex Index (CNXT) | Sonoscape Medical Corp | BBG00MYKWC25 |
| ChiNex Index (CNXT) | Tofflon Science & Technology Group Co L | BBG00F13BGY3 |
| ChiNex Index (CNXT) | Walvax Biotechnology Co Ltd | BBG00F13B014 |
| ChiNex Index (CNXT) | Winner Medical Co Ltd | BBG011C97MD3 |
| ChiNex Index (CNXT) | Yunnan Botanee Bio-Technology Group C | BBG013Y512B5 |
| ChiNex Index (CNXT) | Zhejiang Wolwo Bio-Pharmaceutical Co L | BBG00LBJBZ45 |
| Shenzhen 100 | Botanee | 300957 |
| Shenzhen 100 | Mindray | 300760 |
| Shenzhen 100 | Aier Eye Hospital | 300015 |
| Shenzhen 100 | Zhifei-Biol | 300122 |
| Shenzhen 100 | Walvax | 300142 |
| Shenzhen 100 | CCHN | 661 |
| Shenzhen 100 | Tigermed | 300347 |
| Shenzhen 100 | Asymchem | 2821 |
| Shenzhen 100 | Yunnan Baiyao | 538 |
| Shenzhen 100 | Imeik | 300896 |
| Shenzhen 100 | NHU | 2001 |
| Shenzhen 100 | Kangtai Biological | 300601 |
| Shenzhen 100 | Pharmaron | 300759 |
| Shenzhen 100 | HD Medicine | 963 |
| Shenzhen 100 | Hualan Biological | 2007 |
| Shenzhen 100 | Shanghai RAAS | 2252 |
| Shenzhen 100 | Lepu Medical | 300003 |
| STAR 50 | Bloomage Biotechnology Corporation Lir | 688363 |
| STAR 50 | Cathay Biotech Inc. | 688065 |
| STAR 50 | iRay Technology Company Limited | 688301 |
| STAR 50 | MGI Tech Co., Ltd. | 688114 |
| STAR 50 | Shanghai Junshi Biosciences Co., Ltd. | 688180 |
| STAR 50 | Shanghai United Imaging Healthcare Co., | 688271 |
| STAR 50 | Sichuan Biokin Pharmaceutical Co.,Ltd. | 688506 |
| SSE 180 | BEIJING TONG REN TANG CO., LTD | 600085 |
| SSE 180 | Beijing Wantai Biological Pharmacy Ente | 603392 |
| SSE 180 | Bloomage Biotechnology Corporation Lir | 688363 |
| SSE 180 | Jiangsu Hengrui Pharmaceuticals Co.,Ltd. | 600276 |

| | | |
|---|---|---|
| SSE 180 | Shanghai Fosun Pharmaceutical (Group) | 600196 |
| SSE 180 | Shanghai Junshi Biosciences Co., Ltd. | 688180 |
| SSE 180 | Shanghai Pharmaceuticals Holding Co., L | 601607 |
| SSE 180 | Shanghai United Imaging Healthcare Co., | 688271 |
| SSE 180 | TOPCHOICE MEDICAL CO.INC | 600763 |
| SSE 180 | Yifeng Pharmacy Chain Co., Ltd. | 603939 |
| SSE 180 | Zhejiang Huahai Pharmaceutical Co.,LTD. | 600521 |
| SSE 180 | CHINA MEHECO GROUP CO., LTD. | 600056 |
| SSE 180 | Guangzhou Baiyunshan Pharmaceutical I | 600332 |
| SSE 180 | Proya Cosmetics Co.,LTD | 603605 |
| SSE 180 | WuXi AppTec Co., Ltd. | 603259 |
| SSE 180 | ZHANGZHOU PIENTZEHUANG PHARMAC | 600436 |
| Shenzhen 100 | OCT Holding | 69 |
| Shenzhen 100 | Vanke-A | 2 |
| Shenzhen 100 | CMSK | 1979 |
| SSE 180 | China Communications Construction Co., | 601800 |
| SSE 180 | China Railway Construction Corporation | 601186 |
| SSE 180 | China Railway Group Limited | 601390 |
| SSE 180 | China State Construction Engineering Co | 601668 |
| SSE 180 | Metallurgical Corporation of China Ltd. | 601618 |
| SSE 180 | Poly Developments and Holdings Group ( | 600048 |
| SSE 180 | Seazen Holdings Co.,Ltd. | 601155 |
| SSE 180 | SHANGHAI ZHANGJIANG HI-TECH PARK D | 600895 |
| Shenzhen 100 | Ganfeng Lithium | 2460 |
| Shenzhen 100 | CNGR | 300919 |
| SSE 180 | Ningxia Baofeng Energy Group Co.,Ltd. | 600989 |
| SSE 180 | ALUMINUM CORPORATION OF CHINA LII | 601600 |
| SSE 180 | Chifeng Jilong Gold Mining Co.,Ltd. | 600988 |
| SSE 180 | SHANDONG GOLD MINING CO.,LTD. | 600547 |
| SSE 180 | ZHONGJIN GOLD CORPORATION LIMITED | 600489 |
| SSE 180 | Zijin Mining Group Company Limited | 601899 |
| SSE 180 | CMOC Group Limited | 603993 |
| SSE 180 | Beijing Kingsoft Office Software, Inc. | 688111 |
| SSE 180 | NEW CHINA LIFE INSURANCE COMPANY | 601336 |
| SSE 180 | ORIENT SECURITIES COMPANY LIMITED | 600958 |
| SSE 180 | SHANGHAI PUDONG DEVELOPMENT BAN | 600000 |
| SSE 180 | SOOCHOW SECURITIES CO.,LTD. | 601555 |
| SSE 180 | Yonyou Network Technology Co., Ltd. | 600588 |
| SSE 180 | ZheJiang China Commodities City Group | 600415 |
| SSE 180 | Zheshang Securities Co.,Ltd. | 601878 |
| SSE 180 | 360 Security Technology Inc. | 601360 |
| SSE 180 | AGRICULTURAL BANK OF CHINA LIMITED | 601288 |
| SSE 180 | BANK OF BEIJING CO.,LTD. | 601169 |
| SSE 180 | BANK OF CHINA LIMITED | 601988 |
| SSE 180 | BANK OF COMMUNICATIONS CO.,LTD. | 601328 |
| SSE 180 | BANK OF JIANGSU CO., LTD. | 600919 |
| SSE 180 | BOC International (China) Co., Ltd. | 601696 |
| SSE 180 | CAPITAL SECURITIES CORPORATION LIMI | 601136 |
| SSE 180 | CHINA CITIC BANK CORPORATION LIMITE | 601998 |
| SSE 180 | CHINA CONSTRUCTION BANK CORPORAT | 601939 |
| SSE 180 | CHINA EVERBRIGHT BANK COMPANY LIM | 601818 |
| SSE 180 | INDUSTRIAL AND COMMERCIAL BANK OI | 601398 |
| SSE 180 | INDUSTRIAL BANK CO.,LTD. | 601166 |
| SSE 180 | PING AN INSURANCE（GROUP） COMP/ | 601318 |
| SSE 180 | POSTAL SAVINGS BANK OF CHINA, LTD. | 601658 |
| SSE 180 | The People's Insurance Company (Group | 601319 |
| SSE 180 | China Galaxy Securities Co., Ltd. | 601881 |
| SSE 180 | China Industrial Securities Co.,Ltd | 601377 |
| SSE 180 | China International Capital Corporation I | 601995 |

| | | |
|---|---|---|
| SSE 180 | CHINA LIFE INSURANCE COMPANY LIMIT | 601628 |
| SSE 180 | China Merchants Bank Co., Ltd. | 600036 |
| SSE 180 | China Merchants Securities Co.,Ltd. | 600999 |
| SSE 180 | CHINA MINSHENG BANK | 600016 |
| SSE 180 | China Pacific Insurance (Group) Co., Ltd. | 601601 |
| SSE 180 | CHINA SECURITIES CO., LTD. | 601066 |
| SSE 180 | CINDA SECURITIES CO.,LTD | 601059 |
| SSE 180 | CITIC Securities Company Limited | 600030 |
| SSE 180 | Everbright Securities Company Limited | 601788 |
| SSE 180 | Guotai Junan Securities Co. Ltd. | 601211 |
| SSE 180 | HAITONG Securities Company Limited | 600837 |
| SSE 180 | HUATAI SECURITIES CO.,LTD | 601688 |
| SSE 180 | HUNDSUN TECHNOLOGIES INC. | 600570 |
| SSE 180 | NARI Technology Co., Ltd. | 600406 |
| ChiNex Index (CNXT) | Beijing Enlight Media Co Ltd | BBG00F13CG90 |
| ChiNex Index (CNXT) | East Money Information Co Ltd | BBG00F138WK7 |
| ChiNex Index (CNXT) | Hithink Royalflush Information Network | BBG00F138V10 |
| ChiNex Index (CNXT) | Isoftstone Information Technology Grou | BBG01BWNBPZ2 |
| ChiNex Index (CNXT) | Mango Excellent Media Co Ltd | BBG00F13DSW7 |
| ChiNex Index (CNXT) | Songcheng Performance Development C | BBG00F13B032 |
| ChiNex Index (CNXT) | Thunder Software Technology Co Ltd | BBG00F13F1V5 |
| ChiNex Index (CNXT) | Winning Health Technology Group Co Lt | BBG00F13CGF3 |
| Shenzhen 100 | OFFCN EDU | 2607 |
| Shenzhen 100 | Focus Media | 2027 |
| Shenzhen 100 | Mango | 300413 |
| Shenzhen 100 | EastMoney | 300059 |
| Shenzhen 100 | PAB | 1 |
| Shenzhen 100 | Bank of Ningbo | 2142 |
| Shenzhen 100 | RoyalFlush Info | 300033 |
| STAR 50 | Beijing Kingsoft Office Software, Inc. | 688111 |
| SSE 180 | China Petroleum & Chemical Corporatio | 600028 |
| SSE 180 | COSCO SHIPPING Energy Transportation | 600026 |
| SSE 180 | PetroChina Company Limited | 601857 |
| ChiNex Index (CNXT) | Beijing Compass Technology Developme | BBG00YG5DBJ7 |
| ChiNex Index (CNXT) | Beijing Sinnet Technology Co Ltd | BBG00F13DSG5 |
| ChiNex Index (CNXT) | Eoptolink Technology Inc Ltd | BBG00FNDHXJ1 |
| ChiNex Index (CNXT) | Kunlun Tech Co Ltd | BBG00F13DSY5 |
| ChiNex Index (CNXT) | Longshine Technology Group Co Ltd | BBG00PF5DZX8 |
| ChiNex Index (CNXT) | Sangfor Technologies Inc | BBG00MYKW73 |
| Shenzhen 100 | GF Securities | 776 |
| Shenzhen 100 | Shenwan Hongyuan | 166 |
| Shenzhen 100 | Guosen Securities | 2736 |
| Shenzhen 100 | CNPCCCL | 617 |
| Shenzhen 100 | Iflytek | 2230 |
| Shenzhen 100 | Glodon | 2410 |
| Shenzhen 100 | Sangfor | 300454 |
| Shenzhen 100 | Sanqi Huyu | 2555 |
| Shenzhen 100 | Century Huatong | 2602 |
| Shenzhen 100 | UNIS | 938 |
| Shenzhen 100 | LY iTECH | 2600 |
| Shenzhen 100 | QHSLI | 792 |
| Shenzhen 100 | LBG | 2601 |
| Shenzhen 100 | BNBMPLC | 786 |
| STAR 50 | Qi An Xin Technology Group Inc. | 688561 |
| SSE 180 | CHINA MOBILE LIMITED | 600941 |
| SSE 180 | China Telecom Corporation Limited | 601728 |
| SSE 180 | China Satellite Communications Co., Ltd. | 601698 |
| SSE 180 | CHINA UNITED NETWORK COMMUNICA | 600050 |
| SSE 180 | Foxconn Industrial Internet Co., Ltd. | 601138 |

| | | |
|---|---|---|
| ChiNex Index (CNXT) | Sinofibers Technology Co Ltd | BBG00VC80KM1 |
| ChiNex Index (CNXT) | Suzhou Tfc Optical Communication Co Lt | BBG00R2FQSB2 |
| ChiNex Index (CNXT) | Yealink Network Technology Corp Ltd | BBG00JN03348 |
| ChiNex Index (CNXT) | Zhongji Innolight Co Ltd | BBG00JMZYLD8 |
| Shenzhen 100 | ZTE | 63 |
| Shenzhen 100 | Yealink Network | 300628 |
| STAR 50 | ASR Microelectronics Co., Ltd | 688220 |
| STAR 50 | GUOBO ELECTRONICS CO., LTD. | 688375 |
| SSE 180 | HENGLI PETROCHEMICAL CO.,LTD. | 600346 |
| ChiNex Index (CNXT) | Huali Industrial Group Co Ltd | BBG013Y513M1 |
| Shenzhen 100 | Huali Group | 300979 |
| Shenzhen 100 | Eastern Shenghong | 301 |
| SSE 180 | Beijing-Shanghai High Speed Railway Co. | 601816 |
| SSE 180 | Air China Limited | 601111 |
| SSE 180 | China CSSC Holdings Limited | 600150 |
| SSE 180 | Shanghai International Airport Co., Ltd. | 600009 |
| SSE 180 | CHINA MERCHANTS ENERGY SHIPPING C | 601872 |
| SSE 180 | COSCO SHIPPING Holdings Co., Ltd. | 601919 |
| STAR 50 | China Railway Signal & Communication C | 688009 |
| ChiNex Index (CNXT) | Beijing Originwater Technology Co Ltd | BBG00F1391G9 |

| Sector | D_PES_Water-Scarcity |
| --- | --- |
| Agriculture (animal products) | 5 |
| Agriculture (animal products) | 5 |
| Agriculture (animal products) | 5 |
| Agriculture (animal products) | 5 |
| Agriculture (animal products) | 5 |
| Agriculture (plant products) | 5 |
| Agriculture (plant products) | 5 |
| Appliances & General Goods Manufacturing | 4 |
| Appliances & General Goods Manufacturing | 4 |
| Appliances & General Goods Manufacturing | 4 |
| Appliances & General Goods Manufacturing | 4 |
| Appliances & General Goods Manufacturing | 4 |
| Appliances & General Goods Manufacturing | 4 |
| Appliances & General Goods Manufacturing | 4 |
| Appliances & General Goods Manufacturing | 4 |
| Appliances & General Goods Manufacturing | 4 |
| Automotive, Electrical Equipment & Machinery Production | 4 |
| Automotive, Electrical Equipment & Machinery Production | 4 |
| Automotive, Electrical Equipment & Machinery Production | 4 |
| Automotive, Electrical Equipment & Machinery Production | 4 |
| Automotive, Electrical Equipment & Machinery Production | 4 |
| Automotive, Electrical Equipment & Machinery Production | 4 |
| Automotive, Electrical Equipment & Machinery Production | 4 |
| Automotive, Electrical Equipment & Machinery Production | 4 |
| Automotive, Electrical Equipment & Machinery Production | 4 |
| Automotive, Electrical Equipment & Machinery Production | 4 |
| Automotive, Electrical Equipment & Machinery Production | 4 |
| Automotive, Electrical Equipment & Machinery Production | 4 |
| Automotive, Electrical Equipment & Machinery Production | 4 |
| Automotive, Electrical Equipment & Machinery Production | 4 |
| Automotive, Electrical Equipment & Machinery Production | 4 |
| Automotive, Electrical Equipment & Machinery Production | 4 |
| Automotive, Electrical Equipment & Machinery Production | 4 |
| Automotive, Electrical Equipment & Machinery Production | 4 |
| Automotive, Electrical Equipment & Machinery Production | 4 |
| Automotive, Electrical Equipment & Machinery Production | 4 |
| Automotive, Electrical Equipment & Machinery Production | 4 |
| Automotive, Electrical Equipment & Machinery Production | 4 |
| Automotive, Electrical Equipment & Machinery Production | 4 |
| Automotive, Electrical Equipment & Machinery Production | 4 |
| Automotive, Electrical Equipment & Machinery Production | 4 |
| Automotive, Electrical Equipment & Machinery Production | 4 |
| Automotive, Electrical Equipment & Machinery Production | 4 |
| Automotive, Electrical Equipment & Machinery Production | 4 |
| Automotive, Electrical Equipment & Machinery Production | 4 |
| Automotive, Electrical Equipment & Machinery Production | 4 |
| Automotive, Electrical Equipment & Machinery Production | 4 |
| Automotive, Electrical Equipment & Machinery Production | 4 |
| Automotive, Electrical Equipment & Machinery Production | 4 |
| Automotive, Electrical Equipment & Machinery Production | 4 |
| Automotive, Electrical Equipment & Machinery Production | 4 |
| Automotive, Electrical Equipment & Machinery Production | 4 |
| Automotive, Electrical Equipment & Machinery Production | 4 |
| Automotive, Electrical Equipment & Machinery Production | 4 |

| | |
|---|---|
| Automotive, Electrical Equipment & Machinery Production | 4 |
| Chemicals & Other Materials Production | 4 |
| Chemicals & Other Materials Production | 4 |
| Chemicals & Other Materials Production | 4 |
| Chemicals & Other Materials Production | 4 |
| Chemicals & Other Materials Production | 4 |
| Chemicals & Other Materials Production | 4 |
| Chemicals & Other Materials Production | 4 |
| Chemicals & Other Materials Production | 4 |
| Chemicals & Other Materials Production | 4 |
| Chemicals & Other Materials Production | 4 |
| Chemicals & Other Materials Production | 4 |
| Chemicals & Other Materials Production | 4 |
| Chemicals & Other Materials Production | 4 |
| Chemicals & Other Materials Production | 4 |
| Chemicals & Other Materials Production | 4 |
| Chemicals & Other Materials Production | 4 |
| Chemicals & Other Materials Production | 4 |
| Chemicals & Other Materials Production | 4 |
| Chemicals & Other Materials Production | 4 |
| Chemicals & Other Materials Production | 4 |
| Chemicals & Other Materials Production | 4 |
| Chemicals & Other Materials Production | 4 |
| Chemicals & Other Materials Production | 4 |
| Construction Materials | 5 |
| Construction Materials | 5 |
| Construction Materials | 5 |
| Construction Materials | 5 |
| Construction Materials | 5 |
| Construction Materials | 5 |
| Construction Materials | 5 |
| Construction Materials | 5 |
| Electric Energy Production - Combustion (Biomass, Coal, Gas, Nuclear, Oil), Geothermal Energ | 5 |
| Electric Energy Production - Combustion (Biomass, Coal, Gas, Nuclear, Oil), Geothermal Energ | 5 |
| Electric Energy Production - Combustion (Biomass, Coal, Gas, Nuclear, Oil), Geothermal Energ | 5 |
| Electric Energy Production - Combustion (Biomass, Coal, Gas, Nuclear, Oil), Geothermal Energ | 5 |
| Electric Energy Production - Combustion (Biomass, Coal, Gas, Nuclear, Oil), Geothermal Energ | 5 |
| Electric Energy Production - Combustion (Biomass, Coal, Gas, Nuclear, Oil), Geothermal Energ | 5 |
| Electric Energy Production - Combustion (Biomass, Coal, Gas, Nuclear, Oil), Geothermal Energ | 5 |
| Electric Energy Production - Combustion (Biomass, Coal, Gas, Nuclear, Oil), Geothermal Energ | 5 |
| Electric Energy Production - Combustion (Biomass, Coal, Gas, Nuclear, Oil), Geothermal Energ | 5 |
| Electric Energy Production - Solar, Wind | 3 |
| Electric Energy Production - Solar, Wind | 3 |
| Electric Energy Production - Solar, Wind | 3 |
| Electric Energy Production - Solar, Wind | 3 |
| Electric Energy Production - Solar, Wind | 3 |
| Electric Energy Production - Solar, Wind | 3 |
| Electric Energy Production - Solar, Wind | 3 |
| Electric Energy Production - Solar, Wind | 3 |
| Electric Energy Production - Solar, Wind | 3 |
| Electric Energy Production - Solar, Wind | 3 |
| Electric Energy Production - Solar, Wind | 3 |
| Electric Energy Production - Solar, Wind | 3 |
| Electric Energy Production - Solar, Wind | 3 |
| Electric Energy Production - Solar, Wind | 3 |
| Electric Energy Production – Hydropower | 5 |
| Electronics & Semiconductor Manufacturing | 4 |
| Electronics & Semiconductor Manufacturing | 4 |
| Electronics & Semiconductor Manufacturing | 4 |

| | |
|---|---|
| Electronics & Semiconductor Manufacturing | 4 |
| Electronics & Semiconductor Manufacturing | 4 |
| Electronics & Semiconductor Manufacturing | 4 |
| Electronics & Semiconductor Manufacturing | 4 |
| Electronics & Semiconductor Manufacturing | 4 |
| Electronics & Semiconductor Manufacturing | 4 |
| Electronics & Semiconductor Manufacturing | 4 |
| Electronics & Semiconductor Manufacturing | 4 |
| Electronics & Semiconductor Manufacturing | 4 |
| Electronics & Semiconductor Manufacturing | 4 |
| Electronics & Semiconductor Manufacturing | 4 |
| Electronics & Semiconductor Manufacturing | 4 |
| Electronics & Semiconductor Manufacturing | 4 |
| Electronics & Semiconductor Manufacturing | 4 |
| Electronics & Semiconductor Manufacturing | 4 |
| Electronics & Semiconductor Manufacturing | 4 |
| Electronics & Semiconductor Manufacturing | 4 |
| Electronics & Semiconductor Manufacturing | 4 |
| Electronics & Semiconductor Manufacturing | 4 |
| Electronics & Semiconductor Manufacturing | 4 |
| Electronics & Semiconductor Manufacturing | 4 |
| Electronics & Semiconductor Manufacturing | 4 |
| Electronics & Semiconductor Manufacturing | 4 |
| Electronics & Semiconductor Manufacturing | 4 |
| Electronics & Semiconductor Manufacturing | 4 |
| Electronics & Semiconductor Manufacturing | 4 |
| Electronics & Semiconductor Manufacturing | 4 |
| Electronics & Semiconductor Manufacturing | 4 |
| Electronics & Semiconductor Manufacturing | 4 |
| Electronics & Semiconductor Manufacturing | 4 |
| Electronics & Semiconductor Manufacturing | 4 |
| Electronics & Semiconductor Manufacturing | 4 |
| Electronics & Semiconductor Manufacturing | 4 |
| Electronics & Semiconductor Manufacturing | 4 |
| Electronics & Semiconductor Manufacturing | 4 |
| Electronics & Semiconductor Manufacturing | 4 |
| Electronics & Semiconductor Manufacturing | 4 |
| Electronics & Semiconductor Manufacturing | 4 |
| Electronics & Semiconductor Manufacturing | 4 |
| Electronics & Semiconductor Manufacturing | 4 |
| Electronics & Semiconductor Manufacturing | 4 |
| Electronics & Semiconductor Manufacturing | 4 |
| Electronics & Semiconductor Manufacturing | 4 |
| Electronics & Semiconductor Manufacturing | 4 |
| Electronics & Semiconductor Manufacturing | 4 |
| Electronics & Semiconductor Manufacturing | 4 |
| Electronics & Semiconductor Manufacturing | 4 |
| Electronics & Semiconductor Manufacturing | 4 |
| Electronics & Semiconductor Manufacturing | 4 |
| Electronics & Semiconductor Manufacturing | 4 |
| Electronics & Semiconductor Manufacturing | 4 |
| Electronics & Semiconductor Manufacturing | 4 |
| Electronics & Semiconductor Manufacturing | 4 |
| Electronics & Semiconductor Manufacturing | 4 |
| Electronics & Semiconductor Manufacturing | 4 |
| Electronics & Semiconductor Manufacturing | 4 |
| Electronics & Semiconductor Manufacturing | 4 |
| Electronics & Semiconductor Manufacturing | 4 |
| Electronics & Semiconductor Manufacturing | 4 |

| | |
|---|---|
| Electronics & Semiconductor Manufacturing | 4 |
| Electronics & Semiconductor Manufacturing | 4 |
| Electronics & Semiconductor Manufacturing | 4 |
| Electronics & Semiconductor Manufacturing | 4 |
| Electronics & Semiconductor Manufacturing | 4 |
| Electronics & Semiconductor Manufacturing | 4 |
| Electronics & Semiconductor Manufacturing | 4 |
| Electronics & Semiconductor Manufacturing | 4 |
| Electronics & Semiconductor Manufacturing | 4 |
| Electronics & Semiconductor Manufacturing | 4 |
| Electronics & Semiconductor Manufacturing | 4 |
| Electronics & Semiconductor Manufacturing | 4 |
| Electronics & Semiconductor Manufacturing | 4 |
| Electronics & Semiconductor Manufacturing | 4 |
| Electronics & Semiconductor Manufacturing | 4 |
| Electronics & Semiconductor Manufacturing | 4 |
| Electronics & Semiconductor Manufacturing | 4 |
| Electronics & Semiconductor Manufacturing | 4 |
| Electronics & Semiconductor Manufacturing | 4 |
| Electronics & Semiconductor Manufacturing | 4 |
| Electronics & Semiconductor Manufacturing | 4 |
| Electronics & Semiconductor Manufacturing | 4 |
| Electronics & Semiconductor Manufacturing | 4 |
| Electronics & Semiconductor Manufacturing | 4 |
| Electronics & Semiconductor Manufacturing | 4 |
| Electronics & Semiconductor Manufacturing | 4 |
| Electronics & Semiconductor Manufacturing | 4 |
| Electronics & Semiconductor Manufacturing | 4 |
| Electronics & Semiconductor Manufacturing | 4 |
| Electronics & Semiconductor Manufacturing | 4 |
| Electronics & Semiconductor Manufacturing | 4 |
| Electronics & Semiconductor Manufacturing | 4 |
| Electronics & Semiconductor Manufacturing | 4 |
| Electronics & Semiconductor Manufacturing | 4 |
| Electronics & Semiconductor Manufacturing | 4 |
| Electronics & Semiconductor Manufacturing | 4 |
| Electronics & Semiconductor Manufacturing | 4 |
| Electronics & Semiconductor Manufacturing | 4 |
| Electronics & Semiconductor Manufacturing | 4 |
| Electronics & Semiconductor Manufacturing | 4 |
| Food & Beverage Production | 5 |
| Food & Beverage Production | 5 |
| Food & Beverage Production | 5 |
| Food & Beverage Production | 5 |
| Food & Beverage Production | 5 |
| Food & Beverage Production | 5 |
| Food & Beverage Production | 5 |
| Food & Beverage Production | 5 |
| Food & Beverage Production | 5 |
| Food & Beverage Production | 5 |
| Food & Beverage Production | 5 |
| Food & Beverage Production | 5 |
| Food & Beverage Production | 5 |
| Food & Beverage Production | 5 |
| Food & Beverage Production | 5 |
| Food & Beverage Production | 5 |
| Food & Beverage Production | 5 |
| Food & Beverage Production | 5 |
| Food & Beverage Production | 5 |

| | |
|---|---|
| Food Retailing | 2 |
| General or Speciality Retailing | 2 |
| General or Speciality Retailing | 2 |
| General or Speciality Retailing | 2 |
| General or Speciality Retailing | 2 |
| Health Care, Pharmaceuticals and Biotechnology | 4 |
| Health Care, Pharmaceuticals and Biotechnology | 4 |
| Health Care, Pharmaceuticals and Biotechnology | 4 |
| Health Care, Pharmaceuticals and Biotechnology | 4 |
| Health Care, Pharmaceuticals and Biotechnology | 4 |
| Health Care, Pharmaceuticals and Biotechnology | 4 |
| Health Care, Pharmaceuticals and Biotechnology | 4 |
| Health Care, Pharmaceuticals and Biotechnology | 4 |
| Health Care, Pharmaceuticals and Biotechnology | 4 |
| Health Care, Pharmaceuticals and Biotechnology | 4 |
| Health Care, Pharmaceuticals and Biotechnology | 4 |
| Health Care, Pharmaceuticals and Biotechnology | 4 |
| Health Care, Pharmaceuticals and Biotechnology | 4 |
| Health Care, Pharmaceuticals and Biotechnology | 4 |
| Health Care, Pharmaceuticals and Biotechnology | 4 |
| Health Care, Pharmaceuticals and Biotechnology | 4 |
| Health Care, Pharmaceuticals and Biotechnology | 4 |
| Health Care, Pharmaceuticals and Biotechnology | 4 |
| Health Care, Pharmaceuticals and Biotechnology | 4 |
| Health Care, Pharmaceuticals and Biotechnology | 4 |
| Health Care, Pharmaceuticals and Biotechnology | 4 |
| Health Care, Pharmaceuticals and Biotechnology | 4 |
| Health Care, Pharmaceuticals and Biotechnology | 4 |
| Health Care, Pharmaceuticals and Biotechnology | 4 |
| Health Care, Pharmaceuticals and Biotechnology | 4 |
| Health Care, Pharmaceuticals and Biotechnology | 4 |
| Health Care, Pharmaceuticals and Biotechnology | 4 |
| Health Care, Pharmaceuticals and Biotechnology | 4 |
| Health Care, Pharmaceuticals and Biotechnology | 4 |
| Health Care, Pharmaceuticals and Biotechnology | 4 |
| Health Care, Pharmaceuticals and Biotechnology | 4 |
| Health Care, Pharmaceuticals and Biotechnology | 4 |
| Health Care, Pharmaceuticals and Biotechnology | 4 |
| Health Care, Pharmaceuticals and Biotechnology | 4 |
| Health Care, Pharmaceuticals and Biotechnology | 4 |
| Health Care, Pharmaceuticals and Biotechnology | 4 |
| Health Care, Pharmaceuticals and Biotechnology | 4 |
| Health Care, Pharmaceuticals and Biotechnology | 4 |
| Health Care, Pharmaceuticals and Biotechnology | 4 |
| Health Care, Pharmaceuticals and Biotechnology | 4 |
| Health Care, Pharmaceuticals and Biotechnology | 4 |
| Health Care, Pharmaceuticals and Biotechnology | 4 |
| Health Care, Pharmaceuticals and Biotechnology | 4 |
| Health Care, Pharmaceuticals and Biotechnology | 4 |
| Health Care, Pharmaceuticals and Biotechnology | 4 |
| Health Care, Pharmaceuticals and Biotechnology | 4 |
| Health Care, Pharmaceuticals and Biotechnology | 4 |
| Health Care, Pharmaceuticals and Biotechnology | 4 |
| Health Care, Pharmaceuticals and Biotechnology | 4 |
| Health Care, Pharmaceuticals and Biotechnology | 4 |
| Health Care, Pharmaceuticals and Biotechnology | 4 |
| Health Care, Pharmaceuticals and Biotechnology | 4 |
| Health Care, Pharmaceuticals and Biotechnology | 4 |
| Health Care, Pharmaceuticals and Biotechnology | 4 |

| | |
|---|---|
| Health Care, Pharmaceuticals and Biotechnology | 4 |
| Health Care, Pharmaceuticals and Biotechnology | 4 |
| Health Care, Pharmaceuticals and Biotechnology | 4 |
| Health Care, Pharmaceuticals and Biotechnology | 4 |
| Health Care, Pharmaceuticals and Biotechnology | 4 |
| Health Care, Pharmaceuticals and Biotechnology | 4 |
| Health Care, Pharmaceuticals and Biotechnology | 4 |
| Health Care, Pharmaceuticals and Biotechnology | 4 |
| Health Care, Pharmaceuticals and Biotechnology | 4 |
| Health Care, Pharmaceuticals and Biotechnology | 4 |
| Health Care, Pharmaceuticals and Biotechnology | 4 |
| Health Care, Pharmaceuticals and Biotechnology | 4 |
| Land Development & Construction | 3 |
| Land Development & Construction | 3 |
| Land Development & Construction | 3 |
| Land Development & Construction | 3 |
| Land Development & Construction | 3 |
| Land Development & Construction | 3 |
| Land Development & Construction | 3 |
| Land Development & Construction | 3 |
| Land Development & Construction | 3 |
| Land Development & Construction | 3 |
| Land Development & Construction | 3 |
| Metals & Mining | 5 |
| Metals & Mining | 5 |
| Metals & Mining | 5 |
| Metals & Mining | 5 |
| Metals & Mining | 5 |
| Metals & Mining | 5 |
| Metals & Mining | 5 |
| Metals & Mining | 5 |
| Metals & Mining | 5 |
| Offices & Professional Services | 2 |
| Offices & Professional Services | 2 |
| Offices & Professional Services | 2 |
| Offices & Professional Services | 2 |
| Offices & Professional Services | 2 |
| Offices & Professional Services | 2 |
| Offices & Professional Services | 2 |
| Offices & Professional Services | 2 |
| Offices & Professional Services | 2 |
| Offices & Professional Services | 2 |
| Offices & Professional Services | 2 |
| Offices & Professional Services | 2 |
| Offices & Professional Services | 2 |
| Offices & Professional Services | 2 |
| Offices & Professional Services | 2 |
| Offices & Professional Services | 2 |
| Offices & Professional Services | 2 |
| Offices & Professional Services | 2 |
| Offices & Professional Services | 2 |
| Offices & Professional Services | 2 |
| Offices & Professional Services | 2 |
| Offices & Professional Services | 2 |
| Offices & Professional Services | 2 |
| Offices & Professional Services | 2 |
| Offices & Professional Services | 2 |
| Offices & Professional Services | 2 |
| Offices & Professional Services | 2 |

| | |
|---|---|
| Offices & Professional Services | 2 |
| Offices & Professional Services | 2 |
| Offices & Professional Services | 2 |
| Offices & Professional Services | 2 |
| Offices & Professional Services | 2 |
| Offices & Professional Services | 2 |
| Offices & Professional Services | 2 |
| Offices & Professional Services | 2 |
| Offices & Professional Services | 2 |
| Offices & Professional Services | 2 |
| Offices & Professional Services | 2 |
| Offices & Professional Services | 2 |
| Offices & Professional Services | 2 |
| Offices & Professional Services | 2 |
| Offices & Professional Services | 2 |
| Offices & Professional Services | 2 |
| Offices & Professional Services | 2 |
| Offices & Professional Services | 2 |
| Offices & Professional Services | 2 |
| Offices & Professional Services | 2 |
| Offices & Professional Services | 2 |
| Offices & Professional Services | 2 |
| Offices & Professional Services | 2 |
| Offices & Professional Services | 2 |
| Offices & Professional Services | 2 |
| Offices & Professional Services | 2 |
| Offices & Professional Services | 2 |
| Offices & Professional Services | 2 |
| Offices & Professional Services | 2 |
| Offices & Professional Services | 2 |
| Oil, Gas & Consumable Fuels | 4 |
| Oil, Gas & Consumable Fuels | 4 |
| Oil, Gas & Consumable Fuels | 4 |
| Other (Average of all sectors) | 4 |
| Other (Average of all sectors) | 4 |
| Other (Average of all sectors) | 4 |
| Other (Average of all sectors) | 4 |
| Other (Average of all sectors) | 4 |
| Other (Average of all sectors) | 4 |
| Other (Average of all sectors) | 4 |
| Other (Average of all sectors) | 4 |
| Other (Average of all sectors) | 4 |
| Other (Average of all sectors) | 4 |
| Other (Average of all sectors) | 4 |
| Other (Average of all sectors) | 4 |
| Other (Average of all sectors) | 4 |
| Other (Average of all sectors) | 4 |
| Other (Average of all sectors) | 4 |
| Other (Average of all sectors) | 4 |
| Other (Average of all sectors) | 4 |
| Other (Average of all sectors) | 4 |
| Other (Average of all sectors) | 4 |
| Other (Average of all sectors) | 4 |
| Other (Average of all sectors) | 4 |
| Telecommunication services (including wireless) | 2 |
| Telecommunication services (including wireless) | 2 |
| Telecommunication services (including wireless) | 2 |
| Telecommunication services (including wireless) | 2 |
| Telecommunication services (including wireless) | 2 |

| | |
|---|---|
| Telecommunication services (including wireless) | 2 |
| Telecommunication services (including wireless) | 2 |
| Telecommunication services (including wireless) | 2 |
| Telecommunication services (including wireless) | 2 |
| Telecommunication services (including wireless) | 2 |
| Telecommunication services (including wireless) | 2 |
| Telecommunication services (including wireless) | 2 |
| Telecommunication services (including wireless) | 2 |
| Textiles, Apparel & Luxury Good Production | 5 |
| Textiles, Apparel & Luxury Good Production | 5 |
| Textiles, Apparel & Luxury Good Production | 5 |
| Textiles, Apparel & Luxury Good Production | 5 |
| Transportation Services | 4 |
| Transportation Services | 4 |
| Transportation Services | 4 |
| Transportation Services | 4 |
| Transportation Services | 4 |
| Transportation Services | 4 |
| Transportation Services | 4 |
| Water utilities / Water Service Providers | 5 |

| D_PES_Forest Productivity and Distance to Markets | D_PES_Limited Wild Flora & Fauna Availability | D_PES_Limited Marine Fish Availability | D_REeS_Soil Condition | D_REeS_Water Condition | D_REeS_Air Condition | D_REeS_Ecosystem Condition | D_REeS_Pollination |
|---|---|---|---|---|---|---|---|
| 0 | 1 | 0 | 4 | 5 | 3 | 2 | 2 |
| 0 | 1 | 0 | 4 | 5 | 3 | 2 | 2 |
| 0 | 1 | 0 | 4 | 5 | 3 | 2 | 2 |
| 0 | 1 | 0 | 4 | 5 | 3 | 2 | 2 |
| 0 | 1 | 0 | 4 | 5 | 3 | 2 | 2 |
| 0 | 1 | 0 | 5 | 4 | 3 | 2 | 4 |
| 0 | 1 | 0 | 5 | 4 | 3 | 2 | 4 |
| 0 | 0 | 0 | 0 | 2 | 2 | 0 | 0 |
| 0 | 0 | 0 | 0 | 2 | 2 | 0 | 0 |
| 0 | 0 | 0 | 0 | 2 | 2 | 0 | 0 |
| 0 | 0 | 0 | 0 | 2 | 2 | 0 | 0 |
| 0 | 0 | 0 | 0 | 2 | 2 | 0 | 0 |
| 0 | 0 | 0 | 0 | 2 | 2 | 0 | 0 |
| 0 | 0 | 0 | 0 | 2 | 2 | 0 | 0 |
| 0 | 0 | 0 | 0 | 2 | 2 | 0 | 0 |
| 0 | 0 | 0 | 0 | 2 | 2 | 0 | 0 |
| 0 | 3 | 0 | 0 | 2 | 2 | 0 | 0 |
| 0 | 3 | 0 | 0 | 2 | 2 | 0 | 0 |
| 0 | 3 | 0 | 0 | 2 | 2 | 0 | 0 |
| 0 | 3 | 0 | 0 | 2 | 2 | 0 | 0 |
| 0 | 3 | 0 | 0 | 2 | 2 | 0 | 0 |
| 0 | 3 | 0 | 0 | 2 | 2 | 0 | 0 |
| 0 | 3 | 0 | 0 | 2 | 2 | 0 | 0 |
| 0 | 3 | 0 | 0 | 2 | 2 | 0 | 0 |
| 0 | 3 | 0 | 0 | 2 | 2 | 0 | 0 |
| 0 | 3 | 0 | 0 | 2 | 2 | 0 | 0 |
| 0 | 3 | 0 | 0 | 2 | 2 | 0 | 0 |
| 0 | 3 | 0 | 0 | 2 | 2 | 0 | 0 |
| 0 | 3 | 0 | 0 | 2 | 2 | 0 | 0 |
| 0 | 3 | 0 | 0 | 2 | 2 | 0 | 0 |
| 0 | 3 | 0 | 0 | 2 | 2 | 0 | 0 |
| 0 | 3 | 0 | 0 | 2 | 2 | 0 | 0 |
| 0 | 3 | 0 | 0 | 2 | 2 | 0 | 0 |
| 0 | 3 | 0 | 0 | 2 | 2 | 0 | 0 |
| 0 | 3 | 0 | 0 | 2 | 2 | 0 | 0 |
| 0 | 3 | 0 | 0 | 2 | 2 | 0 | 0 |
| 0 | 3 | 0 | 0 | 2 | 2 | 0 | 0 |
| 0 | 3 | 0 | 0 | 2 | 2 | 0 | 0 |
| 0 | 3 | 0 | 0 | 2 | 2 | 0 | 0 |
| 0 | 3 | 0 | 0 | 2 | 2 | 0 | 0 |
| 0 | 3 | 0 | 0 | 2 | 2 | 0 | 0 |
| 0 | 3 | 0 | 0 | 2 | 2 | 0 | 0 |
| 0 | 3 | 0 | 0 | 2 | 2 | 0 | 0 |
| 0 | 3 | 0 | 0 | 2 | 2 | 0 | 0 |
| 0 | 3 | 0 | 0 | 2 | 2 | 0 | 0 |
| 0 | 3 | 0 | 0 | 2 | 2 | 0 | 0 |
| 0 | 3 | 0 | 0 | 2 | 2 | 0 | 0 |
| 0 | 3 | 0 | 0 | 2 | 2 | 0 | 0 |
| 0 | 3 | 0 | 0 | 2 | 2 | 0 | 0 |
| 0 | 3 | 0 | 0 | 2 | 2 | 0 | 0 |
| 0 | 3 | 0 | 0 | 2 | 2 | 0 | 0 |
| 0 | 3 | 0 | 0 | 2 | 2 | 0 | 0 |
| 0 | 3 | 0 | 0 | 2 | 2 | 0 | 0 |
| 0 | 3 | 0 | 0 | 2 | 2 | 0 | 0 |

| | | | | | | | |
|---|---|---|---|---|---|---|---|
| 0 | 3 | 0 | 0 | 2 | 2 | 0 | 0 |
| 0 | 1 | 0 | 0 | 3 | 2 | 0 | 0 |
| 0 | 1 | 0 | 0 | 3 | 2 | 0 | 0 |
| 0 | 1 | 0 | 0 | 3 | 2 | 0 | 0 |
| 0 | 1 | 0 | 0 | 3 | 2 | 0 | 0 |
| 0 | 1 | 0 | 0 | 3 | 2 | 0 | 0 |
| 0 | 1 | 0 | 0 | 3 | 2 | 0 | 0 |
| 0 | 1 | 0 | 0 | 3 | 2 | 0 | 0 |
| 0 | 1 | 0 | 0 | 3 | 2 | 0 | 0 |
| 0 | 1 | 0 | 0 | 3 | 2 | 0 | 0 |
| 0 | 1 | 0 | 0 | 3 | 2 | 0 | 0 |
| 0 | 1 | 0 | 0 | 3 | 2 | 0 | 0 |
| 0 | 1 | 0 | 0 | 3 | 2 | 0 | 0 |
| 0 | 1 | 0 | 0 | 3 | 2 | 0 | 0 |
| 0 | 1 | 0 | 0 | 3 | 2 | 0 | 0 |
| 0 | 1 | 0 | 0 | 3 | 2 | 0 | 0 |
| 0 | 1 | 0 | 0 | 3 | 2 | 0 | 0 |
| 0 | 1 | 0 | 0 | 3 | 2 | 0 | 0 |
| 0 | 1 | 0 | 0 | 3 | 2 | 0 | 0 |
| 0 | 1 | 0 | 0 | 3 | 2 | 0 | 0 |
| 0 | 1 | 0 | 0 | 3 | 2 | 0 | 0 |
| 0 | 1 | 0 | 0 | 3 | 2 | 0 | 0 |
| 0 | 1 | 0 | 0 | 3 | 2 | 0 | 0 |
| 0 | 1 | 0 | 0 | 3 | 2 | 0 | 0 |
| 4 | 1 | 0 | 0 | 2 | 2 | 0 | 0 |
| 4 | 1 | 0 | 0 | 2 | 2 | 0 | 0 |
| 4 | 1 | 0 | 0 | 2 | 2 | 0 | 0 |
| 4 | 1 | 0 | 0 | 2 | 2 | 0 | 0 |
| 4 | 1 | 0 | 0 | 2 | 2 | 0 | 0 |
| 4 | 1 | 0 | 0 | 2 | 2 | 0 | 0 |
| 4 | 1 | 0 | 0 | 2 | 2 | 0 | 0 |
| 4 | 1 | 0 | 0 | 2 | 2 | 0 | 0 |
| 4 | 0 | 0 | 0 | 2 | 2 | 0 | 0 |
| 4 | 0 | 0 | 0 | 2 | 2 | 0 | 0 |
| 4 | 0 | 0 | 0 | 2 | 2 | 0 | 0 |
| 4 | 0 | 0 | 0 | 2 | 2 | 0 | 0 |
| 4 | 0 | 0 | 0 | 2 | 2 | 0 | 0 |
| 4 | 0 | 0 | 0 | 2 | 2 | 0 | 0 |
| 4 | 0 | 0 | 0 | 2 | 2 | 0 | 0 |
| 4 | 0 | 0 | 0 | 2 | 2 | 0 | 0 |
| 4 | 0 | 0 | 0 | 2 | 2 | 0 | 0 |
| 0 | 0 | 0 | 0 | 2 | 2 | 0 | 0 |
| 0 | 0 | 0 | 0 | 2 | 2 | 0 | 0 |
| 0 | 0 | 0 | 0 | 2 | 2 | 0 | 0 |
| 0 | 0 | 0 | 0 | 2 | 2 | 0 | 0 |
| 0 | 0 | 0 | 0 | 2 | 2 | 0 | 0 |
| 0 | 0 | 0 | 0 | 2 | 2 | 0 | 0 |
| 0 | 0 | 0 | 0 | 2 | 2 | 0 | 0 |
| 0 | 0 | 0 | 0 | 2 | 2 | 0 | 0 |
| 0 | 0 | 0 | 0 | 2 | 2 | 0 | 0 |
| 0 | 0 | 0 | 0 | 2 | 2 | 0 | 0 |
| 0 | 0 | 0 | 0 | 2 | 2 | 0 | 0 |
| 0 | 0 | 0 | 0 | 2 | 2 | 0 | 0 |
| 0 | 0 | 0 | 0 | 2 | 2 | 0 | 0 |
| 0 | 0 | 0 | 0 | 2 | 2 | 0 | 0 |
| 0 | 0 | 0 | 0 | 3 | 1 | 0 | 0 |
| 0 | 0 | 0 | 0 | 2 | 2 | 0 | 0 |
| 0 | 0 | 0 | 0 | 2 | 2 | 0 | 0 |
| 0 | 0 | 0 | 0 | 2 | 2 | 0 | 0 |

| | | | | | | | |
|---|---|---|---|---|---|---|---|
| 0 | 0 | 0 | 0 | 2 | 2 | 0 | 0 |
| 0 | 0 | 0 | 0 | 2 | 2 | 0 | 0 |
| 0 | 0 | 0 | 0 | 2 | 2 | 0 | 0 |
| 0 | 0 | 0 | 0 | 2 | 2 | 0 | 0 |
| 0 | 0 | 0 | 0 | 2 | 2 | 0 | 0 |
| 0 | 0 | 0 | 0 | 2 | 2 | 0 | 0 |
| 0 | 0 | 0 | 0 | 2 | 2 | 0 | 0 |
| 0 | 0 | 0 | 0 | 2 | 2 | 0 | 0 |
| 0 | 0 | 0 | 0 | 2 | 2 | 0 | 0 |
| 0 | 0 | 0 | 0 | 2 | 2 | 0 | 0 |
| 0 | 0 | 0 | 0 | 2 | 2 | 0 | 0 |
| 0 | 0 | 0 | 0 | 2 | 2 | 0 | 0 |
| 0 | 0 | 0 | 0 | 2 | 2 | 0 | 0 |
| 0 | 0 | 0 | 0 | 2 | 2 | 0 | 0 |
| 0 | 0 | 0 | 0 | 2 | 2 | 0 | 0 |
| 0 | 0 | 0 | 0 | 2 | 2 | 0 | 0 |
| 0 | 0 | 0 | 0 | 2 | 2 | 0 | 0 |
| 0 | 0 | 0 | 0 | 2 | 2 | 0 | 0 |
| 0 | 0 | 0 | 0 | 2 | 2 | 0 | 0 |
| 0 | 0 | 0 | 0 | 2 | 2 | 0 | 0 |
| 0 | 0 | 0 | 0 | 2 | 2 | 0 | 0 |
| 0 | 0 | 0 | 0 | 2 | 2 | 0 | 0 |
| 0 | 0 | 0 | 0 | 2 | 2 | 0 | 0 |
| 0 | 0 | 0 | 0 | 2 | 2 | 0 | 0 |
| 0 | 0 | 0 | 0 | 2 | 2 | 0 | 0 |
| 0 | 0 | 0 | 0 | 2 | 2 | 0 | 0 |
| 0 | 0 | 0 | 0 | 2 | 2 | 0 | 0 |
| 0 | 0 | 0 | 0 | 2 | 2 | 0 | 0 |
| 0 | 0 | 0 | 0 | 2 | 2 | 0 | 0 |
| 0 | 0 | 0 | 0 | 2 | 2 | 0 | 0 |
| 0 | 0 | 0 | 0 | 2 | 2 | 0 | 0 |
| 0 | 0 | 0 | 0 | 2 | 2 | 0 | 0 |
| 0 | 0 | 0 | 0 | 2 | 2 | 0 | 0 |
| 0 | 0 | 0 | 0 | 2 | 2 | 0 | 0 |
| 0 | 0 | 0 | 0 | 2 | 2 | 0 | 0 |
| 0 | 0 | 0 | 0 | 2 | 2 | 0 | 0 |
| 0 | 0 | 0 | 0 | 2 | 2 | 0 | 0 |
| 0 | 0 | 0 | 0 | 2 | 2 | 0 | 0 |
| 0 | 0 | 0 | 0 | 2 | 2 | 0 | 0 |
| 0 | 0 | 0 | 0 | 2 | 2 | 0 | 0 |
| 0 | 0 | 0 | 0 | 2 | 2 | 0 | 0 |
| 0 | 0 | 0 | 0 | 2 | 2 | 0 | 0 |
| 0 | 0 | 0 | 0 | 2 | 2 | 0 | 0 |
| 0 | 0 | 0 | 0 | 2 | 2 | 0 | 0 |
| 0 | 0 | 0 | 0 | 2 | 2 | 0 | 0 |
| 0 | 0 | 0 | 0 | 2 | 2 | 0 | 0 |
| 0 | 0 | 0 | 0 | 2 | 2 | 0 | 0 |
| 0 | 0 | 0 | 0 | 2 | 2 | 0 | 0 |
| 0 | 0 | 0 | 0 | 2 | 2 | 0 | 0 |
| 0 | 0 | 0 | 0 | 2 | 2 | 0 | 0 |
| 0 | 0 | 0 | 0 | 2 | 2 | 0 | 0 |
| 0 | 0 | 0 | 0 | 2 | 2 | 0 | 0 |
| 0 | 0 | 0 | 0 | 2 | 2 | 0 | 0 |
| 0 | 0 | 0 | 0 | 2 | 2 | 0 | 0 |
| 0 | 0 | 0 | 0 | 2 | 2 | 0 | 0 |
| 0 | 0 | 0 | 0 | 2 | 2 | 0 | 0 |
| 0 | 0 | 0 | 0 | 2 | 2 | 0 | 0 |
| 0 | 0 | 0 | 0 | 2 | 2 | 0 | 0 |
| 0 | 0 | 0 | 0 | 2 | 2 | 0 | 0 |

| | | | | | | | |
|---|---|---|---|---|---|---|---|
| 0 | 0 | 0 | 0 | 2 | 2 | 0 | 0 |
| 0 | 0 | 0 | 0 | 2 | 2 | 0 | 0 |
| 0 | 0 | 0 | 0 | 2 | 2 | 0 | 0 |
| 0 | 0 | 0 | 0 | 2 | 2 | 0 | 0 |
| 0 | 0 | 0 | 0 | 2 | 2 | 0 | 0 |
| 0 | 0 | 0 | 0 | 2 | 2 | 0 | 0 |
| 0 | 0 | 0 | 0 | 2 | 2 | 0 | 0 |
| 0 | 0 | 0 | 0 | 2 | 2 | 0 | 0 |
| 0 | 0 | 0 | 0 | 2 | 2 | 0 | 0 |
| 0 | 0 | 0 | 0 | 2 | 2 | 0 | 0 |
| 0 | 0 | 0 | 0 | 2 | 2 | 0 | 0 |
| 0 | 0 | 0 | 0 | 2 | 2 | 0 | 0 |
| 0 | 0 | 0 | 0 | 2 | 2 | 0 | 0 |
| 0 | 0 | 0 | 0 | 2 | 2 | 0 | 0 |
| 0 | 0 | 0 | 0 | 2 | 2 | 0 | 0 |
| 0 | 0 | 0 | 0 | 2 | 2 | 0 | 0 |
| 0 | 0 | 0 | 0 | 2 | 2 | 0 | 0 |
| 0 | 0 | 0 | 0 | 2 | 2 | 0 | 0 |
| 0 | 0 | 0 | 0 | 2 | 2 | 0 | 0 |
| 0 | 0 | 0 | 0 | 2 | 2 | 0 | 0 |
| 0 | 0 | 0 | 0 | 2 | 2 | 0 | 0 |
| 0 | 0 | 0 | 0 | 2 | 2 | 0 | 0 |
| 0 | 0 | 0 | 0 | 2 | 2 | 0 | 0 |
| 0 | 0 | 0 | 0 | 2 | 2 | 0 | 0 |
| 0 | 0 | 0 | 0 | 2 | 2 | 0 | 0 |
| 0 | 0 | 0 | 0 | 2 | 2 | 0 | 0 |
| 0 | 0 | 0 | 0 | 2 | 2 | 0 | 0 |
| 0 | 0 | 0 | 0 | 2 | 2 | 0 | 0 |
| 0 | 0 | 0 | 0 | 2 | 2 | 0 | 0 |
| 0 | 0 | 0 | 0 | 2 | 2 | 0 | 0 |
| 0 | 0 | 0 | 0 | 2 | 2 | 0 | 0 |
| 0 | 0 | 0 | 0 | 2 | 2 | 0 | 0 |
| 0 | 0 | 0 | 0 | 2 | 2 | 0 | 0 |
| 0 | 0 | 0 | 0 | 2 | 2 | 0 | 0 |
| 0 | 0 | 0 | 0 | 2 | 2 | 0 | 0 |
| 0 | 0 | 0 | 0 | 2 | 2 | 0 | 0 |
| 0 | 0 | 0 | 0 | 2 | 2 | 0 | 0 |
| 0 | 0 | 0 | 0 | 2 | 2 | 0 | 0 |
| 0 | 0 | 0 | 0 | 2 | 2 | 0 | 0 |
| 0 | 0 | 0 | 0 | 2 | 2 | 0 | 0 |
| 0 | 3 | 0 | 0 | 4 | 2 | 0 | 0 |
| 0 | 3 | 0 | 0 | 4 | 2 | 0 | 0 |
| 0 | 3 | 0 | 0 | 4 | 2 | 0 | 0 |
| 0 | 3 | 0 | 0 | 4 | 2 | 0 | 0 |
| 0 | 3 | 0 | 0 | 4 | 2 | 0 | 0 |
| 0 | 3 | 0 | 0 | 4 | 2 | 0 | 0 |
| 0 | 3 | 0 | 0 | 4 | 2 | 0 | 0 |
| 0 | 3 | 0 | 0 | 4 | 2 | 0 | 0 |
| 0 | 3 | 0 | 0 | 4 | 2 | 0 | 0 |
| 0 | 3 | 0 | 0 | 4 | 2 | 0 | 0 |
| 0 | 3 | 0 | 0 | 4 | 2 | 0 | 0 |
| 0 | 3 | 0 | 0 | 4 | 2 | 0 | 0 |
| 0 | 3 | 0 | 0 | 4 | 2 | 0 | 0 |
| 0 | 3 | 0 | 0 | 4 | 2 | 0 | 0 |
| 0 | 3 | 0 | 0 | 4 | 2 | 0 | 0 |
| 0 | 3 | 0 | 0 | 4 | 2 | 0 | 0 |
| 0 | 3 | 0 | 0 | 4 | 2 | 0 | 0 |
| 0 | 3 | 0 | 0 | 4 | 2 | 0 | 0 |
| 0 | 3 | 0 | 0 | 4 | 2 | 0 | 0 |

| | | | | | | | |
|---|---|---|---|---|---|---|---|
| 0 | 0 | 0 | 0 | 2 | 1 | 0 | 0 |
| 0 | 0 | 0 | 0 | 2 | 1 | 0 | 0 |
| 0 | 0 | 0 | 0 | 2 | 1 | 0 | 0 |
| 0 | 0 | 0 | 0 | 2 | 1 | 0 | 0 |
| 0 | 0 | 0 | 0 | 2 | 1 | 0 | 0 |
| 0 | 3 | 0 | 0 | 3 | 1 | 0 | 0 |
| 0 | 3 | 0 | 0 | 3 | 1 | 0 | 0 |
| 0 | 3 | 0 | 0 | 3 | 1 | 0 | 0 |
| 0 | 3 | 0 | 0 | 3 | 1 | 0 | 0 |
| 0 | 3 | 0 | 0 | 3 | 1 | 0 | 0 |
| 0 | 3 | 0 | 0 | 3 | 1 | 0 | 0 |
| 0 | 3 | 0 | 0 | 3 | 1 | 0 | 0 |
| 0 | 3 | 0 | 0 | 3 | 1 | 0 | 0 |
| 0 | 3 | 0 | 0 | 3 | 1 | 0 | 0 |
| 0 | 3 | 0 | 0 | 3 | 1 | 0 | 0 |
| 0 | 3 | 0 | 0 | 3 | 1 | 0 | 0 |
| 0 | 3 | 0 | 0 | 3 | 1 | 0 | 0 |
| 0 | 3 | 0 | 0 | 3 | 1 | 0 | 0 |
| 0 | 3 | 0 | 0 | 3 | 1 | 0 | 0 |
| 0 | 3 | 0 | 0 | 3 | 1 | 0 | 0 |
| 0 | 3 | 0 | 0 | 3 | 1 | 0 | 0 |
| 0 | 3 | 0 | 0 | 3 | 1 | 0 | 0 |
| 0 | 3 | 0 | 0 | 3 | 1 | 0 | 0 |
| 0 | 3 | 0 | 0 | 3 | 1 | 0 | 0 |
| 0 | 3 | 0 | 0 | 3 | 1 | 0 | 0 |
| 0 | 3 | 0 | 0 | 3 | 1 | 0 | 0 |
| 0 | 3 | 0 | 0 | 3 | 1 | 0 | 0 |
| 0 | 3 | 0 | 0 | 3 | 1 | 0 | 0 |
| 0 | 3 | 0 | 0 | 3 | 1 | 0 | 0 |
| 0 | 3 | 0 | 0 | 3 | 1 | 0 | 0 |
| 0 | 3 | 0 | 0 | 3 | 1 | 0 | 0 |
| 0 | 3 | 0 | 0 | 3 | 1 | 0 | 0 |
| 0 | 3 | 0 | 0 | 3 | 1 | 0 | 0 |
| 0 | 3 | 0 | 0 | 3 | 1 | 0 | 0 |
| 0 | 3 | 0 | 0 | 3 | 1 | 0 | 0 |
| 0 | 3 | 0 | 0 | 3 | 1 | 0 | 0 |
| 0 | 3 | 0 | 0 | 3 | 1 | 0 | 0 |
| 0 | 3 | 0 | 0 | 3 | 1 | 0 | 0 |
| 0 | 3 | 0 | 0 | 3 | 1 | 0 | 0 |
| 0 | 3 | 0 | 0 | 3 | 1 | 0 | 0 |
| 0 | 3 | 0 | 0 | 3 | 1 | 0 | 0 |
| 0 | 3 | 0 | 0 | 3 | 1 | 0 | 0 |
| 0 | 3 | 0 | 0 | 3 | 1 | 0 | 0 |
| 0 | 3 | 0 | 0 | 3 | 1 | 0 | 0 |
| 0 | 3 | 0 | 0 | 3 | 1 | 0 | 0 |
| 0 | 3 | 0 | 0 | 3 | 1 | 0 | 0 |
| 0 | 3 | 0 | 0 | 3 | 1 | 0 | 0 |
| 0 | 3 | 0 | 0 | 3 | 1 | 0 | 0 |
| 0 | 3 | 0 | 0 | 3 | 1 | 0 | 0 |
| 0 | 3 | 0 | 0 | 3 | 1 | 0 | 0 |
| 0 | 3 | 0 | 0 | 3 | 1 | 0 | 0 |
| 0 | 3 | 0 | 0 | 3 | 1 | 0 | 0 |
| 0 | 3 | 0 | 0 | 3 | 1 | 0 | 0 |
| 0 | 3 | 0 | 0 | 3 | 1 | 0 | 0 |
| 0 | 3 | 0 | 0 | 3 | 1 | 0 | 0 |
| 0 | 3 | 0 | 0 | 3 | 1 | 0 | 0 |
| 0 | 3 | 0 | 0 | 3 | 1 | 0 | 0 |
| 0 | 3 | 0 | 0 | 3 | 1 | 0 | 0 |
| 0 | 3 | 0 | 0 | 3 | 1 | 0 | 0 |

| | | | | | | | |
|---|---|---|---|---|---|---|---|
| 0 | 3 | 0 | 0 | 3 | 1 | 0 | 0 |
| 0 | 3 | 0 | 0 | 3 | 1 | 0 | 0 |
| 0 | 3 | 0 | 0 | 3 | 1 | 0 | 0 |
| 0 | 3 | 0 | 0 | 3 | 1 | 0 | 0 |
| 0 | 3 | 0 | 0 | 3 | 1 | 0 | 0 |
| 0 | 3 | 0 | 0 | 3 | 1 | 0 | 0 |
| 0 | 3 | 0 | 0 | 3 | 1 | 0 | 0 |
| 0 | 3 | 0 | 0 | 3 | 1 | 0 | 0 |
| 0 | 3 | 0 | 0 | 3 | 1 | 0 | 0 |
| 0 | 3 | 0 | 0 | 3 | 1 | 0 | 0 |
| 0 | 3 | 0 | 0 | 3 | 1 | 0 | 0 |
| 0 | 3 | 0 | 0 | 3 | 1 | 0 | 0 |
| 4 | 2 | 0 | 1 | 2 | 3 | 0 | 0 |
| 4 | 2 | 0 | 1 | 2 | 3 | 0 | 0 |
| 4 | 2 | 0 | 1 | 2 | 3 | 0 | 0 |
| 4 | 2 | 0 | 1 | 2 | 3 | 0 | 0 |
| 4 | 2 | 0 | 1 | 2 | 3 | 0 | 0 |
| 4 | 2 | 0 | 1 | 2 | 3 | 0 | 0 |
| 4 | 2 | 0 | 1 | 2 | 3 | 0 | 0 |
| 4 | 2 | 0 | 1 | 2 | 3 | 0 | 0 |
| 4 | 2 | 0 | 1 | 2 | 3 | 0 | 0 |
| 4 | 2 | 0 | 1 | 2 | 3 | 0 | 0 |
| 4 | 2 | 0 | 1 | 2 | 3 | 0 | 0 |
| 4 | 0 | 0 | 0 | 2 | 3 | 0 | 0 |
| 4 | 0 | 0 | 0 | 2 | 3 | 0 | 0 |
| 4 | 0 | 0 | 0 | 2 | 3 | 0 | 0 |
| 4 | 0 | 0 | 0 | 2 | 3 | 0 | 0 |
| 4 | 0 | 0 | 0 | 2 | 3 | 0 | 0 |
| 4 | 0 | 0 | 0 | 2 | 3 | 0 | 0 |
| 4 | 0 | 0 | 0 | 2 | 3 | 0 | 0 |
| 4 | 0 | 0 | 0 | 2 | 3 | 0 | 0 |
| 4 | 0 | 0 | 0 | 2 | 3 | 0 | 0 |
| 0 | 0 | 0 | 0 | 2 | 3 | 0 | 0 |
| 0 | 0 | 0 | 0 | 2 | 3 | 0 | 0 |
| 0 | 0 | 0 | 0 | 2 | 3 | 0 | 0 |
| 0 | 0 | 0 | 0 | 2 | 3 | 0 | 0 |
| 0 | 0 | 0 | 0 | 2 | 3 | 0 | 0 |
| 0 | 0 | 0 | 0 | 2 | 3 | 0 | 0 |
| 0 | 0 | 0 | 0 | 2 | 3 | 0 | 0 |
| 0 | 0 | 0 | 0 | 2 | 3 | 0 | 0 |
| 0 | 0 | 0 | 0 | 2 | 3 | 0 | 0 |
| 0 | 0 | 0 | 0 | 2 | 3 | 0 | 0 |
| 0 | 0 | 0 | 0 | 2 | 3 | 0 | 0 |
| 0 | 0 | 0 | 0 | 2 | 3 | 0 | 0 |
| 0 | 0 | 0 | 0 | 2 | 3 | 0 | 0 |
| 0 | 0 | 0 | 0 | 2 | 3 | 0 | 0 |
| 0 | 0 | 0 | 0 | 2 | 3 | 0 | 0 |
| 0 | 0 | 0 | 0 | 2 | 3 | 0 | 0 |
| 0 | 0 | 0 | 0 | 2 | 3 | 0 | 0 |
| 0 | 0 | 0 | 0 | 2 | 3 | 0 | 0 |
| 0 | 0 | 0 | 0 | 2 | 3 | 0 | 0 |
| 0 | 0 | 0 | 0 | 2 | 3 | 0 | 0 |
| 0 | 0 | 0 | 0 | 2 | 3 | 0 | 0 |
| 0 | 0 | 0 | 0 | 2 | 3 | 0 | 0 |
| 0 | 0 | 0 | 0 | 2 | 3 | 0 | 0 |
| 0 | 0 | 0 | 0 | 2 | 3 | 0 | 0 |
| 0 | 0 | 0 | 0 | 2 | 3 | 0 | 0 |
| 0 | 0 | 0 | 0 | 2 | 3 | 0 | 0 |
| 0 | 0 | 0 | 0 | 2 | 3 | 0 | 0 |

| | | | | | | | |
|---|---|---|---|---|---|---|---|
| 0 | 0 | 0 | 0 | 2 | 3 | 0 | 0 |
| 0 | 0 | 0 | 0 | 2 | 3 | 0 | 0 |
| 0 | 0 | 0 | 0 | 2 | 3 | 0 | 0 |
| 0 | 0 | 0 | 0 | 2 | 3 | 0 | 0 |
| 0 | 0 | 0 | 0 | 2 | 3 | 0 | 0 |
| 0 | 0 | 0 | 0 | 2 | 3 | 0 | 0 |
| 0 | 0 | 0 | 0 | 2 | 3 | 0 | 0 |
| 0 | 0 | 0 | 0 | 2 | 3 | 0 | 0 |
| 0 | 0 | 0 | 0 | 2 | 3 | 0 | 0 |
| 0 | 0 | 0 | 0 | 2 | 3 | 0 | 0 |
| 0 | 0 | 0 | 0 | 2 | 3 | 0 | 0 |
| 0 | 0 | 0 | 0 | 2 | 3 | 0 | 0 |
| 0 | 0 | 0 | 0 | 2 | 3 | 0 | 0 |
| 0 | 0 | 0 | 0 | 2 | 3 | 0 | 0 |
| 0 | 0 | 0 | 0 | 2 | 3 | 0 | 0 |
| 0 | 0 | 0 | 0 | 2 | 3 | 0 | 0 |
| 0 | 0 | 0 | 0 | 2 | 3 | 0 | 0 |
| 0 | 0 | 0 | 0 | 2 | 3 | 0 | 0 |
| 0 | 0 | 0 | 0 | 2 | 3 | 0 | 0 |
| 0 | 0 | 0 | 0 | 2 | 3 | 0 | 0 |
| 0 | 0 | 0 | 0 | 2 | 3 | 0 | 0 |
| 0 | 0 | 0 | 0 | 2 | 3 | 0 | 0 |
| 0 | 0 | 0 | 0 | 2 | 3 | 0 | 0 |
| 0 | 0 | 0 | 0 | 2 | 3 | 0 | 0 |
| 0 | 0 | 0 | 0 | 2 | 3 | 0 | 0 |
| 0 | 0 | 0 | 0 | 2 | 3 | 0 | 0 |
| 0 | 0 | 0 | 0 | 2 | 3 | 0 | 0 |
| 0 | 0 | 0 | 0 | 2 | 3 | 0 | 0 |
| 0 | 0 | 0 | 0 | 2 | 3 | 0 | 0 |
| 0 | 0 | 0 | 0 | 2 | 3 | 0 | 0 |
| 4 | 0 | 0 | 0 | 2 | 3 | 0 | 0 |
| 4 | 0 | 0 | 0 | 2 | 3 | 0 | 0 |
| 4 | 0 | 0 | 0 | 2 | 3 | 0 | 0 |
| 1 | 1 | 1 | 1 | 3 | 2 | 1 | 1 |
| 1 | 1 | 1 | 1 | 3 | 2 | 1 | 1 |
| 1 | 1 | 1 | 1 | 3 | 2 | 1 | 1 |
| 1 | 1 | 1 | 1 | 3 | 2 | 1 | 1 |
| 1 | 1 | 1 | 1 | 3 | 2 | 1 | 1 |
| 1 | 1 | 1 | 1 | 3 | 2 | 1 | 1 |
| 1 | 1 | 1 | 1 | 3 | 2 | 1 | 1 |
| 1 | 1 | 1 | 1 | 3 | 2 | 1 | 1 |
| 1 | 1 | 1 | 1 | 3 | 2 | 1 | 1 |
| 1 | 1 | 1 | 1 | 3 | 2 | 1 | 1 |
| 1 | 1 | 1 | 1 | 3 | 2 | 1 | 1 |
| 1 | 1 | 1 | 1 | 3 | 2 | 1 | 1 |
| 1 | 1 | 1 | 1 | 3 | 2 | 1 | 1 |
| 1 | 1 | 1 | 1 | 3 | 2 | 1 | 1 |
| 1 | 1 | 1 | 1 | 3 | 2 | 1 | 1 |
| 1 | 1 | 1 | 1 | 3 | 2 | 1 | 1 |
| 1 | 1 | 1 | 1 | 3 | 2 | 1 | 1 |
| 1 | 1 | 1 | 1 | 3 | 2 | 1 | 1 |
| 1 | 1 | 1 | 1 | 3 | 2 | 1 | 1 |
| 1 | 1 | 1 | 1 | 3 | 2 | 1 | 1 |
| 1 | 1 | 1 | 1 | 3 | 2 | 1 | 1 |
| 2 | 0 | 0 | 0 | 2 | 0 | 0 | 0 |
| 2 | 0 | 0 | 0 | 2 | 0 | 0 | 0 |
| 2 | 0 | 0 | 0 | 2 | 0 | 0 | 0 |
| 2 | 0 | 0 | 0 | 2 | 0 | 0 | 0 |
| 2 | 0 | 0 | 0 | 2 | 0 | 0 | 0 |

| | | | | | | | |
|---|---|---|---|---|---|---|---|
| 2 | 0 | 0 | 0 | 2 | 0 | 0 | 0 |
| 2 | 0 | 0 | 0 | 2 | 0 | 0 | 0 |
| 2 | 0 | 0 | 0 | 2 | 0 | 0 | 0 |
| 2 | 0 | 0 | 0 | 2 | 0 | 0 | 0 |
| 2 | 0 | 0 | 0 | 2 | 0 | 0 | 0 |
| 2 | 0 | 0 | 0 | 2 | 0 | 0 | 0 |
| 2 | 0 | 0 | 0 | 2 | 0 | 0 | 0 |
| 2 | 0 | 0 | 0 | 2 | 0 | 0 | 0 |
| 5 | 2 | 0 | 0 | 2 | 2 | 0 | 0 |
| 5 | 2 | 0 | 0 | 2 | 2 | 0 | 0 |
| 5 | 2 | 0 | 0 | 2 | 2 | 0 | 0 |
| 5 | 2 | 0 | 0 | 2 | 2 | 0 | 0 |
| 2 | 0 | 0 | 0 | 2 | 2 | 0 | 0 |
| 2 | 0 | 0 | 0 | 2 | 2 | 0 | 0 |
| 2 | 0 | 0 | 0 | 2 | 2 | 0 | 0 |
| 2 | 0 | 0 | 0 | 2 | 2 | 0 | 0 |
| 2 | 0 | 0 | 0 | 2 | 2 | 0 | 0 |
| 2 | 0 | 0 | 0 | 2 | 2 | 0 | 0 |
| 2 | 0 | 0 | 0 | 2 | 2 | 0 | 0 |
| 0 | 0 | 0 | 1 | 4 | 2 | 0 | 0 |

| D_REmS_Landslides | D_REmS_Wildfire Hazard | D_REmS_Plant/Forest/Aquatic Pests and Diseases | D_REmS_Herbicide Resistance | D_REmS_Extreme Heat | D_REmS_Tropical Cyclones | D_CS_Tourism Attractiveness | D_Rep_Media Scrutiny |
|---|---|---|---|---|---|---|---|
| 5 | 4 | 4 | 4 | 5 | 5 | 0 | 5 |
| 5 | 4 | 4 | 4 | 5 | 5 | 0 | 5 |
| 5 | 4 | 4 | 4 | 5 | 5 | 0 | 5 |
| 5 | 4 | 4 | 4 | 5 | 5 | 0 | 5 |
| 5 | 4 | 4 | 4 | 5 | 5 | 0 | 5 |
| 5 | 4 | 4 | 4 | 5 | 5 | 0 | 5 |
| 5 | 4 | 4 | 4 | 5 | 5 | 0 | 5 |
| 4 | 3 | 0 | 0 | 3 | 4 | 0 | 3 |
| 4 | 3 | 0 | 0 | 3 | 4 | 0 | 3 |
| 4 | 3 | 0 | 0 | 3 | 4 | 0 | 3 |
| 4 | 3 | 0 | 0 | 3 | 4 | 0 | 3 |
| 4 | 3 | 0 | 0 | 3 | 4 | 0 | 3 |
| 4 | 3 | 0 | 0 | 3 | 4 | 0 | 3 |
| 4 | 3 | 0 | 0 | 3 | 4 | 0 | 3 |
| 4 | 3 | 0 | 0 | 3 | 4 | 0 | 3 |
| 4 | 3 | 0 | 0 | 3 | 4 | 0 | 3 |
| 4 | 3 | 0 | 0 | 3 | 4 | 0 | 1 |
| 4 | 3 | 0 | 0 | 3 | 4 | 0 | 1 |
| 4 | 3 | 0 | 0 | 3 | 4 | 0 | 1 |
| 4 | 3 | 0 | 0 | 3 | 4 | 0 | 1 |
| 4 | 3 | 0 | 0 | 3 | 4 | 0 | 1 |
| 4 | 3 | 0 | 0 | 3 | 4 | 0 | 1 |
| 4 | 3 | 0 | 0 | 3 | 4 | 0 | 1 |
| 4 | 3 | 0 | 0 | 3 | 4 | 0 | 1 |
| 4 | 3 | 0 | 0 | 3 | 4 | 0 | 1 |
| 4 | 3 | 0 | 0 | 3 | 4 | 0 | 1 |
| 4 | 3 | 0 | 0 | 3 | 4 | 0 | 1 |
| 4 | 3 | 0 | 0 | 3 | 4 | 0 | 1 |
| 4 | 3 | 0 | 0 | 3 | 4 | 0 | 1 |
| 4 | 3 | 0 | 0 | 3 | 4 | 0 | 1 |
| 4 | 3 | 0 | 0 | 3 | 4 | 0 | 1 |
| 4 | 3 | 0 | 0 | 3 | 4 | 0 | 1 |
| 4 | 3 | 0 | 0 | 3 | 4 | 0 | 1 |
| 4 | 3 | 0 | 0 | 3 | 4 | 0 | 1 |
| 4 | 3 | 0 | 0 | 3 | 4 | 0 | 1 |
| 4 | 3 | 0 | 0 | 3 | 4 | 0 | 1 |
| 4 | 3 | 0 | 0 | 3 | 4 | 0 | 1 |
| 4 | 3 | 0 | 0 | 3 | 4 | 0 | 1 |
| 4 | 3 | 0 | 0 | 3 | 4 | 0 | 1 |
| 4 | 3 | 0 | 0 | 3 | 4 | 0 | 1 |
| 4 | 3 | 0 | 0 | 3 | 4 | 0 | 1 |
| 4 | 3 | 0 | 0 | 3 | 4 | 0 | 1 |
| 4 | 3 | 0 | 0 | 3 | 4 | 0 | 1 |
| 4 | 3 | 0 | 0 | 3 | 4 | 0 | 1 |
| 4 | 3 | 0 | 0 | 3 | 4 | 0 | 1 |
| 4 | 3 | 0 | 0 | 3 | 4 | 0 | 1 |
| 4 | 3 | 0 | 0 | 3 | 4 | 0 | 1 |
| 4 | 3 | 0 | 0 | 3 | 4 | 0 | 1 |
| 4 | 3 | 0 | 0 | 3 | 4 | 0 | 1 |
| 4 | 3 | 0 | 0 | 3 | 4 | 0 | 1 |
| 4 | 3 | 0 | 0 | 3 | 4 | 0 | 1 |
| 4 | 3 | 0 | 0 | 3 | 4 | 0 | 1 |
| 4 | 3 | 0 | 0 | 3 | 4 | 0 | 1 |
| 4 | 3 | 0 | 0 | 3 | 4 | 0 | 1 |

| | | | | | | | |
|---|---|---|---|---|---|---|---|
| 4 | 3 | 0 | 0 | 3 | 4 | 0 | 1 |
| 4 | 3 | 0 | 0 | 3 | 4 | 0 | 4 |
| 4 | 3 | 0 | 0 | 3 | 4 | 0 | 4 |
| 4 | 3 | 0 | 0 | 3 | 4 | 0 | 4 |
| 4 | 3 | 0 | 0 | 3 | 4 | 0 | 4 |
| 4 | 3 | 0 | 0 | 3 | 4 | 0 | 4 |
| 4 | 3 | 0 | 0 | 3 | 4 | 0 | 4 |
| 4 | 3 | 0 | 0 | 3 | 4 | 0 | 4 |
| 4 | 3 | 0 | 0 | 3 | 4 | 0 | 4 |
| 4 | 3 | 0 | 0 | 3 | 4 | 0 | 4 |
| 4 | 3 | 0 | 0 | 3 | 4 | 0 | 4 |
| 4 | 3 | 0 | 0 | 3 | 4 | 0 | 4 |
| 4 | 3 | 0 | 0 | 3 | 4 | 0 | 4 |
| 4 | 3 | 0 | 0 | 3 | 4 | 0 | 4 |
| 4 | 3 | 0 | 0 | 3 | 4 | 0 | 4 |
| 4 | 3 | 0 | 0 | 3 | 4 | 0 | 4 |
| 4 | 3 | 0 | 0 | 3 | 4 | 0 | 4 |
| 4 | 3 | 0 | 0 | 3 | 4 | 0 | 4 |
| 4 | 3 | 0 | 0 | 3 | 4 | 0 | 4 |
| 4 | 3 | 0 | 0 | 3 | 4 | 0 | 4 |
| 4 | 3 | 0 | 0 | 3 | 4 | 0 | 4 |
| 4 | 3 | 0 | 0 | 3 | 4 | 0 | 4 |
| 4 | 3 | 0 | 0 | 3 | 4 | 0 | 4 |
| 4 | 3 | 0 | 0 | 3 | 4 | 0 | 4 |
| 4 | 3 | 0 | 0 | 3 | 4 | 0 | 4 |
| 4 | 3 | 0 | 0 | 3 | 4 | 0 | 4 |
| 4 | 3 | 0 | 0 | 3 | 4 | 0 | 4 |
| 4 | 3 | 0 | 0 | 3 | 4 | 0 | 4 |
| 4 | 3 | 0 | 0 | 3 | 4 | 0 | 4 |
| 4 | 3 | 0 | 0 | 3 | 4 | 0 | 4 |
| 4 | 3 | 0 | 0 | 3 | 4 | 0 | 4 |
| 4 | 3 | 0 | 0 | 3 | 4 | 0 | 4 |
| 4 | 3 | 0 | 0 | 4 | 4 | 0 | 2 |
| 4 | 3 | 0 | 0 | 4 | 4 | 0 | 2 |
| 4 | 3 | 0 | 0 | 4 | 4 | 0 | 2 |
| 4 | 3 | 0 | 0 | 4 | 4 | 0 | 2 |
| 4 | 3 | 0 | 0 | 4 | 4 | 0 | 2 |
| 4 | 3 | 0 | 0 | 4 | 4 | 0 | 2 |
| 4 | 3 | 0 | 0 | 4 | 4 | 0 | 2 |
| 4 | 3 | 0 | 0 | 4 | 4 | 0 | 2 |
| 4 | 3 | 0 | 0 | 4 | 4 | 0 | 2 |
| 4 | 3 | 0 | 0 | 3 | 4 | 0 | 2 |
| 4 | 3 | 0 | 0 | 3 | 4 | 0 | 2 |
| 4 | 3 | 0 | 0 | 3 | 4 | 0 | 2 |
| 4 | 3 | 0 | 0 | 3 | 4 | 0 | 2 |
| 4 | 3 | 0 | 0 | 3 | 4 | 0 | 2 |
| 4 | 3 | 0 | 0 | 3 | 4 | 0 | 2 |
| 4 | 3 | 0 | 0 | 3 | 4 | 0 | 2 |
| 4 | 3 | 0 | 0 | 3 | 4 | 0 | 2 |
| 4 | 3 | 0 | 0 | 3 | 4 | 0 | 2 |
| 4 | 3 | 0 | 0 | 3 | 4 | 0 | 2 |
| 4 | 3 | 0 | 0 | 3 | 4 | 0 | 2 |
| 4 | 3 | 0 | 0 | 3 | 4 | 0 | 2 |
| 4 | 3 | 0 | 0 | 3 | 4 | 0 | 2 |
| 4 | 3 | 0 | 0 | 3 | 4 | 0 | 2 |
| 4 | 3 | 0 | 0 | 3 | 4 | 0 | 2 |
| 4 | 3 | 0 | 0 | 3 | 4 | 0 | 1 |
| 4 | 3 | 0 | 0 | 3 | 4 | 0 | 1 |
| 4 | 3 | 0 | 0 | 3 | 4 | 0 | 1 |

| | | | | | | | |
|---|---|---|---|---|---|---|---|
| 4 | 3 | 0 | 0 | 3 | 4 | 0 | 1 |
| 4 | 3 | 0 | 0 | 3 | 4 | 0 | 1 |
| 4 | 3 | 0 | 0 | 3 | 4 | 0 | 1 |
| 4 | 3 | 0 | 0 | 3 | 4 | 0 | 1 |
| 4 | 3 | 0 | 0 | 3 | 4 | 0 | 1 |
| 4 | 3 | 0 | 0 | 3 | 4 | 0 | 1 |
| 4 | 3 | 0 | 0 | 3 | 4 | 0 | 1 |
| 4 | 3 | 0 | 0 | 3 | 4 | 0 | 1 |
| 4 | 3 | 0 | 0 | 3 | 4 | 0 | 1 |
| 4 | 3 | 0 | 0 | 3 | 4 | 0 | 1 |
| 4 | 3 | 0 | 0 | 3 | 4 | 0 | 1 |
| 4 | 3 | 0 | 0 | 3 | 4 | 0 | 1 |
| 4 | 3 | 0 | 0 | 3 | 4 | 0 | 1 |
| 4 | 3 | 0 | 0 | 3 | 4 | 0 | 1 |
| 4 | 3 | 0 | 0 | 3 | 4 | 0 | 1 |
| 4 | 3 | 0 | 0 | 3 | 4 | 0 | 1 |
| 4 | 3 | 0 | 0 | 3 | 4 | 0 | 1 |
| 4 | 3 | 0 | 0 | 3 | 4 | 0 | 1 |
| 4 | 3 | 0 | 0 | 3 | 4 | 0 | 1 |
| 4 | 3 | 0 | 0 | 3 | 4 | 0 | 1 |
| 4 | 3 | 0 | 0 | 3 | 4 | 0 | 1 |
| 4 | 3 | 0 | 0 | 3 | 4 | 0 | 1 |
| 4 | 3 | 0 | 0 | 3 | 4 | 0 | 1 |
| 4 | 3 | 0 | 0 | 3 | 4 | 0 | 1 |
| 4 | 3 | 0 | 0 | 3 | 4 | 0 | 1 |
| 4 | 3 | 0 | 0 | 3 | 4 | 0 | 1 |
| 4 | 3 | 0 | 0 | 3 | 4 | 0 | 1 |
| 4 | 3 | 0 | 0 | 3 | 4 | 0 | 1 |
| 4 | 3 | 0 | 0 | 3 | 4 | 0 | 1 |
| 4 | 3 | 0 | 0 | 3 | 4 | 0 | 1 |
| 4 | 3 | 0 | 0 | 3 | 4 | 0 | 1 |
| 4 | 3 | 0 | 0 | 3 | 4 | 0 | 1 |
| 4 | 3 | 0 | 0 | 3 | 4 | 0 | 1 |
| 4 | 3 | 0 | 0 | 3 | 4 | 0 | 1 |
| 4 | 3 | 0 | 0 | 3 | 4 | 0 | 1 |
| 4 | 3 | 0 | 0 | 3 | 4 | 0 | 1 |
| 4 | 3 | 0 | 0 | 3 | 4 | 0 | 1 |
| 4 | 3 | 0 | 0 | 3 | 4 | 0 | 1 |
| 4 | 3 | 0 | 0 | 3 | 4 | 0 | 1 |
| 4 | 3 | 0 | 0 | 3 | 4 | 0 | 1 |
| 4 | 3 | 0 | 0 | 3 | 4 | 0 | 1 |
| 4 | 3 | 0 | 0 | 3 | 4 | 0 | 1 |
| 4 | 3 | 0 | 0 | 3 | 4 | 0 | 1 |
| 4 | 3 | 0 | 0 | 3 | 4 | 0 | 1 |
| 4 | 3 | 0 | 0 | 3 | 4 | 0 | 1 |
| 4 | 3 | 0 | 0 | 3 | 4 | 0 | 1 |
| 4 | 3 | 0 | 0 | 3 | 4 | 0 | 1 |
| 4 | 3 | 0 | 0 | 3 | 4 | 0 | 1 |
| 4 | 3 | 0 | 0 | 3 | 4 | 0 | 1 |
| 4 | 3 | 0 | 0 | 3 | 4 | 0 | 1 |
| 4 | 3 | 0 | 0 | 3 | 4 | 0 | 1 |
| 4 | 3 | 0 | 0 | 3 | 4 | 0 | 1 |
| 4 | 3 | 0 | 0 | 3 | 4 | 0 | 1 |
| 4 | 3 | 0 | 0 | 3 | 4 | 0 | 1 |
| 4 | 3 | 0 | 0 | 3 | 4 | 0 | 1 |
| 4 | 3 | 0 | 0 | 3 | 4 | 0 | 1 |
| 4 | 3 | 0 | 0 | 3 | 4 | 0 | 1 |
| 4 | 3 | 0 | 0 | 3 | 4 | 0 | 1 |
| 4 | 3 | 0 | 0 | 3 | 4 | 0 | 1 |

| | | | | | | | |
|---|---|---|---|---|---|---|---|
| 4 | 3 | 0 | 0 | 3 | 4 | 0 | 1 |
| 4 | 3 | 0 | 0 | 3 | 4 | 0 | 1 |
| 4 | 3 | 0 | 0 | 3 | 4 | 0 | 1 |
| 4 | 3 | 0 | 0 | 3 | 4 | 0 | 1 |
| 4 | 3 | 0 | 0 | 3 | 4 | 0 | 1 |
| 4 | 3 | 0 | 0 | 3 | 4 | 0 | 1 |
| 4 | 3 | 0 | 0 | 3 | 4 | 0 | 1 |
| 4 | 3 | 0 | 0 | 3 | 4 | 0 | 1 |
| 4 | 3 | 0 | 0 | 3 | 4 | 0 | 1 |
| 4 | 3 | 0 | 0 | 3 | 4 | 0 | 1 |
| 4 | 3 | 0 | 0 | 3 | 4 | 0 | 1 |
| 4 | 3 | 0 | 0 | 3 | 4 | 0 | 1 |
| 4 | 3 | 0 | 0 | 3 | 4 | 0 | 1 |
| 4 | 3 | 0 | 0 | 3 | 4 | 0 | 1 |
| 4 | 3 | 0 | 0 | 3 | 4 | 0 | 1 |
| 4 | 3 | 0 | 0 | 3 | 4 | 0 | 1 |
| 4 | 3 | 0 | 0 | 3 | 4 | 0 | 1 |
| 4 | 3 | 0 | 0 | 3 | 4 | 0 | 1 |
| 4 | 3 | 0 | 0 | 3 | 4 | 0 | 1 |
| 4 | 3 | 0 | 0 | 3 | 4 | 0 | 1 |
| 4 | 3 | 0 | 0 | 3 | 4 | 0 | 1 |
| 4 | 3 | 0 | 0 | 3 | 4 | 0 | 1 |
| 4 | 3 | 0 | 0 | 3 | 4 | 0 | 1 |
| 4 | 3 | 0 | 0 | 3 | 4 | 0 | 1 |
| 4 | 3 | 0 | 0 | 3 | 4 | 0 | 1 |
| 4 | 3 | 0 | 0 | 3 | 4 | 0 | 1 |
| 4 | 3 | 0 | 0 | 3 | 4 | 0 | 1 |
| 4 | 3 | 0 | 0 | 3 | 4 | 0 | 1 |
| 4 | 3 | 0 | 0 | 3 | 4 | 0 | 1 |
| 4 | 3 | 0 | 0 | 3 | 4 | 0 | 1 |
| 4 | 3 | 0 | 0 | 3 | 4 | 0 | 1 |
| 4 | 3 | 0 | 0 | 3 | 4 | 0 | 1 |
| 4 | 3 | 0 | 0 | 3 | 4 | 0 | 1 |
| 4 | 3 | 0 | 0 | 3 | 4 | 0 | 1 |
| 4 | 3 | 0 | 0 | 3 | 4 | 0 | 1 |
| 4 | 3 | 0 | 0 | 3 | 4 | 0 | 1 |
| 4 | 3 | 0 | 0 | 3 | 4 | 0 | 1 |
| 4 | 3 | 0 | 0 | 3 | 4 | 0 | 1 |
| 4 | 3 | 0 | 0 | 3 | 4 | 0 | 1 |
| 4 | 3 | 0 | 0 | 3 | 4 | 0 | 1 |
| 4 | 3 | 4 | 0 | 3 | 4 | 0 | 5 |
| 4 | 3 | 4 | 0 | 3 | 4 | 0 | 5 |
| 4 | 3 | 4 | 0 | 3 | 4 | 0 | 5 |
| 4 | 3 | 4 | 0 | 3 | 4 | 0 | 5 |
| 4 | 3 | 4 | 0 | 3 | 4 | 0 | 5 |
| 4 | 3 | 4 | 0 | 3 | 4 | 0 | 5 |
| 4 | 3 | 4 | 0 | 3 | 4 | 0 | 5 |
| 4 | 3 | 4 | 0 | 3 | 4 | 0 | 5 |
| 4 | 3 | 4 | 0 | 3 | 4 | 0 | 5 |
| 4 | 3 | 4 | 0 | 3 | 4 | 0 | 5 |
| 4 | 3 | 4 | 0 | 3 | 4 | 0 | 5 |
| 4 | 3 | 4 | 0 | 3 | 4 | 0 | 5 |
| 4 | 3 | 4 | 0 | 3 | 4 | 0 | 5 |
| 4 | 3 | 4 | 0 | 3 | 4 | 0 | 5 |
| 4 | 3 | 4 | 0 | 3 | 4 | 0 | 5 |
| 4 | 3 | 4 | 0 | 3 | 4 | 0 | 5 |
| 4 | 3 | 4 | 0 | 3 | 4 | 0 | 5 |
| 4 | 3 | 4 | 0 | 3 | 4 | 0 | 5 |
| 4 | 3 | 4 | 0 | 3 | 4 | 0 | 5 |

| | | | | | | | |
|---|---|---|---|---|---|---|---|
| 4 | 3 | 0 | 0 | 3 | 4 | 0 | 3 |
| 4 | 3 | 0 | 0 | 3 | 4 | 0 | 3 |
| 4 | 3 | 0 | 0 | 3 | 4 | 0 | 3 |
| 4 | 3 | 0 | 0 | 3 | 4 | 0 | 3 |
| 4 | 3 | 0 | 0 | 3 | 4 | 0 | 3 |
| 4 | 3 | 4 | 0 | 3 | 4 | 0 | 1 |
| 4 | 3 | 4 | 0 | 3 | 4 | 0 | 1 |
| 4 | 3 | 4 | 0 | 3 | 4 | 0 | 1 |
| 4 | 3 | 4 | 0 | 3 | 4 | 0 | 1 |
| 4 | 3 | 4 | 0 | 3 | 4 | 0 | 1 |
| 4 | 3 | 4 | 0 | 3 | 4 | 0 | 1 |
| 4 | 3 | 4 | 0 | 3 | 4 | 0 | 1 |
| 4 | 3 | 4 | 0 | 3 | 4 | 0 | 1 |
| 4 | 3 | 4 | 0 | 3 | 4 | 0 | 1 |
| 4 | 3 | 4 | 0 | 3 | 4 | 0 | 1 |
| 4 | 3 | 4 | 0 | 3 | 4 | 0 | 1 |
| 4 | 3 | 4 | 0 | 3 | 4 | 0 | 1 |
| 4 | 3 | 4 | 0 | 3 | 4 | 0 | 1 |
| 4 | 3 | 4 | 0 | 3 | 4 | 0 | 1 |
| 4 | 3 | 4 | 0 | 3 | 4 | 0 | 1 |
| 4 | 3 | 4 | 0 | 3 | 4 | 0 | 1 |
| 4 | 3 | 4 | 0 | 3 | 4 | 0 | 1 |
| 4 | 3 | 4 | 0 | 3 | 4 | 0 | 1 |
| 4 | 3 | 4 | 0 | 3 | 4 | 0 | 1 |
| 4 | 3 | 4 | 0 | 3 | 4 | 0 | 1 |
| 4 | 3 | 4 | 0 | 3 | 4 | 0 | 1 |
| 4 | 3 | 4 | 0 | 3 | 4 | 0 | 1 |
| 4 | 3 | 4 | 0 | 3 | 4 | 0 | 1 |
| 4 | 3 | 4 | 0 | 3 | 4 | 0 | 1 |
| 4 | 3 | 4 | 0 | 3 | 4 | 0 | 1 |
| 4 | 3 | 4 | 0 | 3 | 4 | 0 | 1 |
| 4 | 3 | 4 | 0 | 3 | 4 | 0 | 1 |
| 4 | 3 | 4 | 0 | 3 | 4 | 0 | 1 |
| 4 | 3 | 4 | 0 | 3 | 4 | 0 | 1 |
| 4 | 3 | 4 | 0 | 3 | 4 | 0 | 1 |
| 4 | 3 | 4 | 0 | 3 | 4 | 0 | 1 |
| 4 | 3 | 4 | 0 | 3 | 4 | 0 | 1 |
| 4 | 3 | 4 | 0 | 3 | 4 | 0 | 1 |
| 4 | 3 | 4 | 0 | 3 | 4 | 0 | 1 |
| 4 | 3 | 4 | 0 | 3 | 4 | 0 | 1 |
| 4 | 3 | 4 | 0 | 3 | 4 | 0 | 1 |
| 4 | 3 | 4 | 0 | 3 | 4 | 0 | 1 |
| 4 | 3 | 4 | 0 | 3 | 4 | 0 | 1 |
| 4 | 3 | 4 | 0 | 3 | 4 | 0 | 1 |
| 4 | 3 | 4 | 0 | 3 | 4 | 0 | 1 |
| 4 | 3 | 4 | 0 | 3 | 4 | 0 | 1 |
| 4 | 3 | 4 | 0 | 3 | 4 | 0 | 1 |
| 4 | 3 | 4 | 0 | 3 | 4 | 0 | 1 |
| 4 | 3 | 4 | 0 | 3 | 4 | 0 | 1 |
| 4 | 3 | 4 | 0 | 3 | 4 | 0 | 1 |
| 4 | 3 | 4 | 0 | 3 | 4 | 0 | 1 |
| 4 | 3 | 4 | 0 | 3 | 4 | 0 | 1 |
| 4 | 3 | 4 | 0 | 3 | 4 | 0 | 1 |
| 4 | 3 | 4 | 0 | 3 | 4 | 0 | 1 |
| 4 | 3 | 4 | 0 | 3 | 4 | 0 | 1 |
| 4 | 3 | 4 | 0 | 3 | 4 | 0 | 1 |
| 4 | 3 | 4 | 0 | 3 | 4 | 0 | 1 |
| 4 | 3 | 4 | 0 | 3 | 4 | 0 | 1 |
| 4 | 3 | 4 | 0 | 3 | 4 | 0 | 1 |

| | | | | | | | |
|---|---|---|---|---|---|---|---|
| 4 | 3 | 4 | 0 | 3 | 4 | 0 | 1 |
| 4 | 3 | 4 | 0 | 3 | 4 | 0 | 1 |
| 4 | 3 | 4 | 0 | 3 | 4 | 0 | 1 |
| 4 | 3 | 4 | 0 | 3 | 4 | 0 | 1 |
| 4 | 3 | 4 | 0 | 3 | 4 | 0 | 1 |
| 4 | 3 | 4 | 0 | 3 | 4 | 0 | 1 |
| 4 | 3 | 4 | 0 | 3 | 4 | 0 | 1 |
| 4 | 3 | 4 | 0 | 3 | 4 | 0 | 1 |
| 4 | 3 | 4 | 0 | 3 | 4 | 0 | 1 |
| 4 | 3 | 4 | 0 | 3 | 4 | 0 | 1 |
| 4 | 3 | 4 | 0 | 3 | 4 | 0 | 1 |
| 4 | 3 | 4 | 0 | 3 | 4 | 0 | 1 |
| 4 | 3 | 0 | 1 | 4 | 4 | 1 | 4 |
| 4 | 3 | 0 | 1 | 4 | 4 | 1 | 4 |
| 4 | 3 | 0 | 1 | 4 | 4 | 1 | 4 |
| 4 | 3 | 0 | 1 | 4 | 4 | 1 | 4 |
| 4 | 3 | 0 | 1 | 4 | 4 | 1 | 4 |
| 4 | 3 | 0 | 1 | 4 | 4 | 1 | 4 |
| 4 | 3 | 0 | 1 | 4 | 4 | 1 | 4 |
| 4 | 3 | 0 | 1 | 4 | 4 | 1 | 4 |
| 4 | 3 | 0 | 1 | 4 | 4 | 1 | 4 |
| 4 | 3 | 0 | 1 | 4 | 4 | 1 | 4 |
| 4 | 3 | 0 | 1 | 4 | 4 | 1 | 4 |
| 4 | 3 | 0 | 0 | 4 | 4 | 0 | 5 |
| 4 | 3 | 0 | 0 | 4 | 4 | 0 | 5 |
| 4 | 3 | 0 | 0 | 4 | 4 | 0 | 5 |
| 4 | 3 | 0 | 0 | 4 | 4 | 0 | 5 |
| 4 | 3 | 0 | 0 | 4 | 4 | 0 | 5 |
| 4 | 3 | 0 | 0 | 4 | 4 | 0 | 5 |
| 4 | 3 | 0 | 0 | 4 | 4 | 0 | 5 |
| 4 | 3 | 0 | 0 | 4 | 4 | 0 | 5 |
| 4 | 3 | 0 | 0 | 4 | 4 | 0 | 5 |
| 4 | 3 | 0 | 0 | 4 | 4 | 0 | 3 |
| 4 | 3 | 0 | 0 | 4 | 4 | 0 | 3 |
| 4 | 3 | 0 | 0 | 4 | 4 | 0 | 3 |
| 4 | 3 | 0 | 0 | 4 | 4 | 0 | 3 |
| 4 | 3 | 0 | 0 | 4 | 4 | 0 | 3 |
| 4 | 3 | 0 | 0 | 4 | 4 | 0 | 3 |
| 4 | 3 | 0 | 0 | 4 | 4 | 0 | 3 |
| 4 | 3 | 0 | 0 | 4 | 4 | 0 | 3 |
| 4 | 3 | 0 | 0 | 4 | 4 | 0 | 3 |
| 4 | 3 | 0 | 0 | 4 | 4 | 0 | 3 |
| 4 | 3 | 0 | 0 | 4 | 4 | 0 | 3 |
| 4 | 3 | 0 | 0 | 4 | 4 | 0 | 3 |
| 4 | 3 | 0 | 0 | 4 | 4 | 0 | 3 |
| 4 | 3 | 0 | 0 | 4 | 4 | 0 | 3 |
| 4 | 3 | 0 | 0 | 4 | 4 | 0 | 3 |
| 4 | 3 | 0 | 0 | 4 | 4 | 0 | 3 |
| 4 | 3 | 0 | 0 | 4 | 4 | 0 | 3 |
| 4 | 3 | 0 | 0 | 4 | 4 | 0 | 3 |
| 4 | 3 | 0 | 0 | 4 | 4 | 0 | 3 |
| 4 | 3 | 0 | 0 | 4 | 4 | 0 | 3 |
| 4 | 3 | 0 | 0 | 4 | 4 | 0 | 3 |
| 4 | 3 | 0 | 0 | 4 | 4 | 0 | 3 |
| 4 | 3 | 0 | 0 | 4 | 4 | 0 | 3 |
| 4 | 3 | 0 | 0 | 4 | 4 | 0 | 3 |
| 4 | 3 | 0 | 0 | 4 | 4 | 0 | 3 |
| 4 | 3 | 0 | 0 | 4 | 4 | 0 | 3 |
| 4 | 3 | 0 | 0 | 4 | 4 | 0 | 3 |

| | | | | | | | |
|---|---|---|---|---|---|---|---|
| 4 | 3 | 0 | 0 | 4 | 4 | 0 | 3 |
| 4 | 3 | 0 | 0 | 4 | 4 | 0 | 3 |
| 4 | 3 | 0 | 0 | 4 | 4 | 0 | 3 |
| 4 | 3 | 0 | 0 | 4 | 4 | 0 | 3 |
| 4 | 3 | 0 | 0 | 4 | 4 | 0 | 3 |
| 4 | 3 | 0 | 0 | 4 | 4 | 0 | 3 |
| 4 | 3 | 0 | 0 | 4 | 4 | 0 | 3 |
| 4 | 3 | 0 | 0 | 4 | 4 | 0 | 3 |
| 4 | 3 | 0 | 0 | 4 | 4 | 0 | 3 |
| 4 | 3 | 0 | 0 | 4 | 4 | 0 | 3 |
| 4 | 3 | 0 | 0 | 4 | 4 | 0 | 3 |
| 4 | 3 | 0 | 0 | 4 | 4 | 0 | 3 |
| 4 | 3 | 0 | 0 | 4 | 4 | 0 | 3 |
| 4 | 3 | 0 | 0 | 4 | 4 | 0 | 3 |
| 4 | 3 | 0 | 0 | 4 | 4 | 0 | 3 |
| 4 | 3 | 0 | 0 | 4 | 4 | 0 | 3 |
| 4 | 3 | 0 | 0 | 4 | 4 | 0 | 3 |
| 4 | 3 | 0 | 0 | 4 | 4 | 0 | 3 |
| 4 | 3 | 0 | 0 | 4 | 4 | 0 | 3 |
| 4 | 3 | 0 | 0 | 4 | 4 | 0 | 3 |
| 4 | 3 | 0 | 0 | 4 | 4 | 0 | 3 |
| 4 | 3 | 0 | 0 | 4 | 4 | 0 | 3 |
| 4 | 3 | 0 | 0 | 4 | 4 | 0 | 3 |
| 4 | 3 | 0 | 0 | 4 | 4 | 0 | 3 |
| 4 | 3 | 0 | 0 | 4 | 4 | 0 | 3 |
| 4 | 3 | 0 | 0 | 4 | 4 | 0 | 3 |
| 4 | 3 | 0 | 0 | 4 | 4 | 0 | 3 |
| 4 | 3 | 0 | 0 | 4 | 4 | 0 | 3 |
| 4 | 3 | 0 | 0 | 4 | 4 | 0 | 3 |
| 4 | 3 | 0 | 0 | 4 | 4 | 0 | 3 |
| 4 | 3 | 0 | 0 | 4 | 4 | 0 | 4 |
| 4 | 3 | 0 | 0 | 4 | 4 | 0 | 4 |
| 4 | 3 | 0 | 0 | 4 | 4 | 0 | 4 |
| 4 | 3 | 1 | 1 | 4 | 4 | 1 | 3 |
| 4 | 3 | 1 | 1 | 4 | 4 | 1 | 3 |
| 4 | 3 | 1 | 1 | 4 | 4 | 1 | 3 |
| 4 | 3 | 1 | 1 | 4 | 4 | 1 | 3 |
| 4 | 3 | 1 | 1 | 4 | 4 | 1 | 3 |
| 4 | 3 | 1 | 1 | 4 | 4 | 1 | 3 |
| 4 | 3 | 1 | 1 | 4 | 4 | 1 | 3 |
| 4 | 3 | 1 | 1 | 4 | 4 | 1 | 3 |
| 4 | 3 | 1 | 1 | 4 | 4 | 1 | 3 |
| 4 | 3 | 1 | 1 | 4 | 4 | 1 | 3 |
| 4 | 3 | 1 | 1 | 4 | 4 | 1 | 3 |
| 4 | 3 | 1 | 1 | 4 | 4 | 1 | 3 |
| 4 | 3 | 1 | 1 | 4 | 4 | 1 | 3 |
| 4 | 3 | 1 | 1 | 4 | 4 | 1 | 3 |
| 4 | 3 | 1 | 1 | 4 | 4 | 1 | 3 |
| 4 | 3 | 1 | 1 | 4 | 4 | 1 | 3 |
| 4 | 3 | 1 | 1 | 4 | 4 | 1 | 3 |
| 4 | 3 | 1 | 1 | 4 | 4 | 1 | 3 |
| 4 | 3 | 1 | 1 | 4 | 4 | 1 | 3 |
| 4 | 3 | 1 | 1 | 4 | 4 | 1 | 3 |
| 4 | 3 | 1 | 1 | 4 | 4 | 1 | 3 |
| 4 | 3 | 0 | 0 | 3 | 4 | 0 | 1 |
| 4 | 3 | 0 | 0 | 3 | 4 | 0 | 1 |
| 4 | 3 | 0 | 0 | 3 | 4 | 0 | 1 |
| 4 | 3 | 0 | 0 | 3 | 4 | 0 | 1 |
| 4 | 3 | 0 | 0 | 3 | 4 | 0 | 1 |

| | | | | | | | |
|---|---|---|---|---|---|---|---|
| 4 | 3 | 0 | 0 | 3 | 4 | 0 | 1 |
| 4 | 3 | 0 | 0 | 3 | 4 | 0 | 1 |
| 4 | 3 | 0 | 0 | 3 | 4 | 0 | 1 |
| 4 | 3 | 0 | 0 | 3 | 4 | 0 | 1 |
| 4 | 3 | 0 | 0 | 3 | 4 | 0 | 1 |
| 4 | 3 | 0 | 0 | 3 | 4 | 0 | 1 |
| 4 | 3 | 0 | 0 | 3 | 4 | 0 | 1 |
| 4 | 3 | 0 | 0 | 3 | 4 | 0 | 1 |
| 4 | 3 | 0 | 0 | 3 | 4 | 0 | 3 |
| 4 | 3 | 0 | 0 | 3 | 4 | 0 | 3 |
| 4 | 3 | 0 | 0 | 3 | 4 | 0 | 3 |
| 4 | 3 | 0 | 0 | 3 | 4 | 0 | 3 |
| 5 | 4 | 0 | 0 | 4 | 5 | 0 | 1 |
| 5 | 4 | 0 | 0 | 4 | 5 | 0 | 1 |
| 5 | 4 | 0 | 0 | 4 | 5 | 0 | 1 |
| 5 | 4 | 0 | 0 | 4 | 5 | 0 | 1 |
| 5 | 4 | 0 | 0 | 4 | 5 | 0 | 1 |
| 5 | 4 | 0 | 0 | 4 | 5 | 0 | 1 |
| 5 | 4 | 0 | 0 | 4 | 5 | 0 | 1 |
| 5 | 4 | 0 | 2 | 4 | 5 | 0 | 5 |

| D_Rep_Political Situation | D_Rep_Sites of International Interest | D_Rep_Risk Preparation | Dependency_Median | Physical-risk_Median | I_Biod_Land, Freshwater and Sea Use Change | I_Biod_Tree Cover Loss | I_Biod_Invasives |
|---|---|---|---|---|---|---|---|
| 3 | 3 | 2 | 4 | High | 5 | 5 | 3 |
| 3 | 3 | 2 | 4 | High | 5 | 5 | 3 |
| 3 | 3 | 2 | 4 | High | 5 | 5 | 3 |
| 3 | 3 | 2 | 4 | High | 5 | 5 | 3 |
| 3 | 3 | 2 | 4 | High | 5 | 5 | 3 |
| 3 | 3 | 2 | 4 | High | 5 | 5 | 3 |
| 3 | 3 | 2 | 4 | High | 5 | 5 | 3 |
| 2 | 2 | 2 | 2 | Low | 1 | 1 | 0 |
| 2 | 2 | 2 | 2 | Low | 1 | 1 | 0 |
| 2 | 2 | 2 | 2 | Low | 1 | 1 | 0 |
| 2 | 2 | 2 | 2 | Low | 1 | 1 | 0 |
| 2 | 2 | 2 | 2 | Low | 1 | 1 | 0 |
| 2 | 2 | 2 | 2 | Low | 1 | 1 | 0 |
| 2 | 2 | 2 | 2 | Low | 1 | 1 | 0 |
| 2 | 2 | 2 | 2 | Low | 1 | 1 | 0 |
| 2 | 2 | 2 | 2 | Low | 1 | 1 | 0 |
| 2 | 2 | 2 | 2 | Low | 1 | 1 | 0 |
| 2 | 2 | 2 | 2 | Low | 1 | 1 | 0 |
| 2 | 2 | 2 | 2 | Low | 1 | 1 | 0 |
| 2 | 2 | 2 | 2 | Low | 1 | 1 | 0 |
| 2 | 2 | 2 | 2 | Low | 1 | 1 | 0 |
| 2 | 2 | 2 | 2 | Low | 1 | 1 | 0 |
| 2 | 2 | 2 | 2 | Low | 1 | 1 | 0 |
| 2 | 2 | 2 | 2 | Low | 1 | 1 | 0 |
| 2 | 2 | 2 | 2 | Low | 1 | 1 | 0 |
| 2 | 2 | 2 | 2 | Low | 1 | 1 | 0 |
| 2 | 2 | 2 | 2 | Low | 1 | 1 | 0 |
| 2 | 2 | 2 | 2 | Low | 1 | 1 | 0 |
| 2 | 2 | 2 | 2 | Low | 1 | 1 | 0 |
| 2 | 2 | 2 | 2 | Low | 1 | 1 | 0 |
| 2 | 2 | 2 | 2 | Low | 1 | 1 | 0 |
| 2 | 2 | 2 | 2 | Low | 1 | 1 | 0 |
| 2 | 2 | 2 | 2 | Low | 1 | 1 | 0 |
| 2 | 2 | 2 | 2 | Low | 1 | 1 | 0 |
| 2 | 2 | 2 | 2 | Low | 1 | 1 | 0 |
| 2 | 2 | 2 | 2 | Low | 1 | 1 | 0 |
| 2 | 2 | 2 | 2 | Low | 1 | 1 | 0 |
| 2 | 2 | 2 | 2 | Low | 1 | 1 | 0 |
| 2 | 2 | 2 | 2 | Low | 1 | 1 | 0 |
| 2 | 2 | 2 | 2 | Low | 1 | 1 | 0 |
| 2 | 2 | 2 | 2 | Low | 1 | 1 | 0 |
| 2 | 2 | 2 | 2 | Low | 1 | 1 | 0 |
| 2 | 2 | 2 | 2 | Low | 1 | 1 | 0 |
| 2 | 2 | 2 | 2 | Low | 1 | 1 | 0 |
| 2 | 2 | 2 | 2 | Low | 1 | 1 | 0 |
| 2 | 2 | 2 | 2 | Low | 1 | 1 | 0 |
| 2 | 2 | 2 | 2 | Low | 1 | 1 | 0 |
| 2 | 2 | 2 | 2 | Low | 1 | 1 | 0 |
| 2 | 2 | 2 | 2 | Low | 1 | 1 | 0 |
| 2 | 2 | 2 | 2 | Low | 1 | 1 | 0 |
| 2 | 2 | 2 | 2 | Low | 1 | 1 | 0 |
| 2 | 2 | 2 | 2 | Low | 1 | 1 | 0 |
| 2 | 2 | 2 | 2 | Low | 1 | 1 | 0 |
| 2 | 2 | 2 | 2 | Low | 1 | 1 | 0 |

| | | | | | | | |
|---|---|---|---|---|---|---|---|
| 2 | 2 | 2 | 2 | Low | 1 | 1 | 0 |
| 2 | 2 | 2 | 2 | Low | 1 | 1 | 0 |
| 2 | 2 | 2 | 2 | Low | 1 | 1 | 0 |
| 2 | 2 | 2 | 2 | Low | 1 | 1 | 0 |
| 2 | 2 | 2 | 2 | Low | 1 | 1 | 0 |
| 2 | 2 | 2 | 2 | Low | 1 | 1 | 0 |
| 2 | 2 | 2 | 2 | Low | 1 | 1 | 0 |
| 2 | 2 | 2 | 2 | Low | 1 | 1 | 0 |
| 2 | 2 | 2 | 2 | Low | 1 | 1 | 0 |
| 2 | 2 | 2 | 2 | Low | 1 | 1 | 0 |
| 2 | 2 | 2 | 2 | Low | 1 | 1 | 0 |
| 2 | 2 | 2 | 2 | Low | 1 | 1 | 0 |
| 2 | 2 | 2 | 2 | Low | 1 | 1 | 0 |
| 2 | 2 | 2 | 2 | Low | 1 | 1 | 0 |
| 2 | 2 | 2 | 2 | Low | 1 | 1 | 0 |
| 2 | 2 | 2 | 2 | Low | 1 | 1 | 0 |
| 2 | 2 | 2 | 2 | Low | 1 | 1 | 0 |
| 2 | 2 | 2 | 2 | Low | 1 | 1 | 0 |
| 2 | 2 | 2 | 2 | Low | 1 | 1 | 0 |
| 2 | 2 | 2 | 2 | Low | 1 | 1 | 0 |
| 2 | 2 | 2 | 2 | Low | 1 | 1 | 0 |
| 2 | 2 | 2 | 2 | Low | 1 | 1 | 0 |
| 2 | 2 | 2 | 2 | Low | 1 | 1 | 0 |
| 2 | 2 | 2 | 2 | Low | 1 | 1 | 0 |
| 2 | 2 | 2 | 2 | Low | 1 | 5 | 2 |
| 2 | 2 | 2 | 2 | Low | 1 | 5 | 2 |
| 2 | 2 | 2 | 2 | Low | 1 | 5 | 2 |
| 2 | 2 | 2 | 2 | Low | 1 | 5 | 2 |
| 2 | 2 | 2 | 2 | Low | 1 | 5 | 2 |
| 2 | 2 | 2 | 2 | Low | 1 | 5 | 2 |
| 2 | 2 | 2 | 2 | Low | 1 | 5 | 2 |
| 2 | 2 | 2 | 2 | Low | 1 | 5 | 2 |
| 3 | 3 | 2 | 2 | Low | 1 | 4 | 0 |
| 3 | 3 | 2 | 2 | Low | 1 | 4 | 0 |
| 3 | 3 | 2 | 2 | Low | 1 | 4 | 0 |
| 3 | 3 | 2 | 2 | Low | 1 | 4 | 0 |
| 3 | 3 | 2 | 2 | Low | 1 | 4 | 0 |
| 3 | 3 | 2 | 2 | Low | 1 | 4 | 0 |
| 3 | 3 | 2 | 2 | Low | 1 | 4 | 0 |
| 3 | 3 | 2 | 2 | Low | 1 | 4 | 0 |
| 3 | 3 | 2 | 2 | Low | 1 | 4 | 0 |
| 2 | 2 | 2 | 2 | Low | 3 | 1 | 0 |
| 2 | 2 | 2 | 2 | Low | 3 | 1 | 0 |
| 2 | 2 | 2 | 2 | Low | 3 | 1 | 0 |
| 2 | 2 | 2 | 2 | Low | 3 | 1 | 0 |
| 2 | 2 | 2 | 2 | Low | 3 | 1 | 0 |
| 2 | 2 | 2 | 2 | Low | 3 | 1 | 0 |
| 2 | 2 | 2 | 2 | Low | 3 | 1 | 0 |
| 2 | 2 | 2 | 2 | Low | 3 | 1 | 0 |
| 2 | 2 | 2 | 2 | Low | 3 | 1 | 0 |
| 2 | 2 | 2 | 2 | Low | 3 | 1 | 0 |
| 2 | 2 | 2 | 2 | Low | 3 | 1 | 0 |
| 2 | 2 | 2 | 2 | Low | 3 | 1 | 0 |
| 2 | 2 | 2 | 2 | Low | 3 | 1 | 0 |
| 2 | 2 | 2 | 2 | Low | 3 | 1 | 0 |
| 3 | 3 | 2 | 2 | Low | 5 | 4 | 2 |
| 2 | 2 | 2 | 2 | Low | 1 | 1 | 0 |
| 2 | 2 | 2 | 2 | Low | 1 | 1 | 0 |
| 2 | 2 | 2 | 2 | Low | 1 | 1 | 0 |

| | | | | | | | |
|---|---|---|---|---|---|---|---|
| 2 | 2 | 2 | 2 | Low | 1 | 1 | 0 |
| 2 | 2 | 2 | 2 | Low | 1 | 1 | 0 |
| 2 | 2 | 2 | 2 | Low | 1 | 1 | 0 |
| 2 | 2 | 2 | 2 | Low | 1 | 1 | 0 |
| 2 | 2 | 2 | 2 | Low | 1 | 1 | 0 |
| 2 | 2 | 2 | 2 | Low | 1 | 1 | 0 |
| 2 | 2 | 2 | 2 | Low | 1 | 1 | 0 |
| 2 | 2 | 2 | 2 | Low | 1 | 1 | 0 |
| 2 | 2 | 2 | 2 | Low | 1 | 1 | 0 |
| 2 | 2 | 2 | 2 | Low | 1 | 1 | 0 |
| 2 | 2 | 2 | 2 | Low | 1 | 1 | 0 |
| 2 | 2 | 2 | 2 | Low | 1 | 1 | 0 |
| 2 | 2 | 2 | 2 | Low | 1 | 1 | 0 |
| 2 | 2 | 2 | 2 | Low | 1 | 1 | 0 |
| 2 | 2 | 2 | 2 | Low | 1 | 1 | 0 |
| 2 | 2 | 2 | 2 | Low | 1 | 1 | 0 |
| 2 | 2 | 2 | 2 | Low | 1 | 1 | 0 |
| 2 | 2 | 2 | 2 | Low | 1 | 1 | 0 |
| 2 | 2 | 2 | 2 | Low | 1 | 1 | 0 |
| 2 | 2 | 2 | 2 | Low | 1 | 1 | 0 |
| 2 | 2 | 2 | 2 | Low | 1 | 1 | 0 |
| 2 | 2 | 2 | 2 | Low | 1 | 1 | 0 |
| 2 | 2 | 2 | 2 | Low | 1 | 1 | 0 |
| 2 | 2 | 2 | 2 | Low | 1 | 1 | 0 |
| 2 | 2 | 2 | 2 | Low | 1 | 1 | 0 |
| 2 | 2 | 2 | 2 | Low | 1 | 1 | 0 |
| 2 | 2 | 2 | 2 | Low | 1 | 1 | 0 |
| 2 | 2 | 2 | 2 | Low | 1 | 1 | 0 |
| 2 | 2 | 2 | 2 | Low | 1 | 1 | 0 |
| 2 | 2 | 2 | 2 | Low | 1 | 1 | 0 |
| 2 | 2 | 2 | 2 | Low | 1 | 1 | 0 |
| 2 | 2 | 2 | 2 | Low | 1 | 1 | 0 |
| 2 | 2 | 2 | 2 | Low | 1 | 1 | 0 |
| 2 | 2 | 2 | 2 | Low | 1 | 1 | 0 |
| 2 | 2 | 2 | 2 | Low | 1 | 1 | 0 |
| 2 | 2 | 2 | 2 | Low | 1 | 1 | 0 |
| 2 | 2 | 2 | 2 | Low | 1 | 1 | 0 |
| 2 | 2 | 2 | 2 | Low | 1 | 1 | 0 |
| 2 | 2 | 2 | 2 | Low | 1 | 1 | 0 |
| 2 | 2 | 2 | 2 | Low | 1 | 1 | 0 |
| 2 | 2 | 2 | 2 | Low | 1 | 1 | 0 |
| 2 | 2 | 2 | 2 | Low | 1 | 1 | 0 |
| 2 | 2 | 2 | 2 | Low | 1 | 1 | 0 |
| 2 | 2 | 2 | 2 | Low | 1 | 1 | 0 |
| 2 | 2 | 2 | 2 | Low | 1 | 1 | 0 |
| 2 | 2 | 2 | 2 | Low | 1 | 1 | 0 |
| 2 | 2 | 2 | 2 | Low | 1 | 1 | 0 |
| 2 | 2 | 2 | 2 | Low | 1 | 1 | 0 |
| 2 | 2 | 2 | 2 | Low | 1 | 1 | 0 |
| 2 | 2 | 2 | 2 | Low | 1 | 1 | 0 |
| 2 | 2 | 2 | 2 | Low | 1 | 1 | 0 |
| 2 | 2 | 2 | 2 | Low | 1 | 1 | 0 |
| 2 | 2 | 2 | 2 | Low | 1 | 1 | 0 |
| 2 | 2 | 2 | 2 | Low | 1 | 1 | 0 |
| 2 | 2 | 2 | 2 | Low | 1 | 1 | 0 |
| 2 | 2 | 2 | 2 | Low | 1 | 1 | 0 |
| 2 | 2 | 2 | 2 | Low | 1 | 1 | 0 |
| 2 | 2 | 2 | 2 | Low | 1 | 1 | 0 |
| 2 | 2 | 2 | 2 | Low | 1 | 1 | 0 |

| | | | | | | | |
|---|---|---|---|---|---|---|---|
| 2 | 2 | 2 | 2 | Low | 1 | 1 | 0 |
| 2 | 2 | 2 | 2 | Low | 1 | 1 | 0 |
| 2 | 2 | 2 | 2 | Low | 1 | 1 | 0 |
| 2 | 2 | 2 | 2 | Low | 1 | 1 | 0 |
| 2 | 2 | 2 | 2 | Low | 1 | 1 | 0 |
| 2 | 2 | 2 | 2 | Low | 1 | 1 | 0 |
| 2 | 2 | 2 | 2 | Low | 1 | 1 | 0 |
| 2 | 2 | 2 | 2 | Low | 1 | 1 | 0 |
| 2 | 2 | 2 | 2 | Low | 1 | 1 | 0 |
| 2 | 2 | 2 | 2 | Low | 1 | 1 | 0 |
| 2 | 2 | 2 | 2 | Low | 1 | 1 | 0 |
| 2 | 2 | 2 | 2 | Low | 1 | 1 | 0 |
| 2 | 2 | 2 | 2 | Low | 1 | 1 | 0 |
| 2 | 2 | 2 | 2 | Low | 1 | 1 | 0 |
| 2 | 2 | 2 | 2 | Low | 1 | 1 | 0 |
| 2 | 2 | 2 | 2 | Low | 1 | 1 | 0 |
| 2 | 2 | 2 | 2 | Low | 1 | 1 | 0 |
| 2 | 2 | 2 | 2 | Low | 1 | 1 | 0 |
| 2 | 2 | 2 | 2 | Low | 1 | 1 | 0 |
| 2 | 2 | 2 | 2 | Low | 1 | 1 | 0 |
| 2 | 2 | 2 | 2 | Low | 1 | 1 | 0 |
| 2 | 2 | 2 | 2 | Low | 1 | 1 | 0 |
| 2 | 2 | 2 | 2 | Low | 1 | 1 | 0 |
| 2 | 2 | 2 | 2 | Low | 1 | 1 | 0 |
| 2 | 2 | 2 | 2 | Low | 1 | 1 | 0 |
| 2 | 2 | 2 | 2 | Low | 1 | 1 | 0 |
| 2 | 2 | 2 | 2 | Low | 1 | 1 | 0 |
| 2 | 2 | 2 | 2 | Low | 1 | 1 | 0 |
| 2 | 2 | 2 | 2 | Low | 1 | 1 | 0 |
| 2 | 2 | 2 | 2 | Low | 1 | 1 | 0 |
| 2 | 2 | 2 | 2 | Low | 1 | 1 | 0 |
| 2 | 2 | 2 | 2 | Low | 1 | 1 | 0 |
| 2 | 2 | 2 | 2 | Low | 1 | 1 | 0 |
| 2 | 2 | 2 | 2 | Low | 1 | 1 | 0 |
| 2 | 2 | 2 | 2 | Low | 1 | 1 | 0 |
| 2 | 2 | 2 | 2 | Low | 1 | 1 | 0 |
| 2 | 2 | 2 | 2 | Low | 1 | 1 | 0 |
| 2 | 2 | 2 | 2 | Low | 1 | 1 | 0 |
| 2 | 2 | 2 | 2 | Low | 1 | 1 | 0 |
| 2 | 2 | 2 | 2 | Low | 1 | 1 | 0 |
| 3 | 2 | 2 | 3 | Medium | 1 | 1 | 2 |
| 3 | 2 | 2 | 3 | Medium | 1 | 1 | 2 |
| 3 | 2 | 2 | 3 | Medium | 1 | 1 | 2 |
| 3 | 2 | 2 | 3 | Medium | 1 | 1 | 2 |
| 3 | 2 | 2 | 3 | Medium | 1 | 1 | 2 |
| 3 | 2 | 2 | 3 | Medium | 1 | 1 | 2 |
| 3 | 2 | 2 | 3 | Medium | 1 | 1 | 2 |
| 3 | 2 | 2 | 3 | Medium | 1 | 1 | 2 |
| 3 | 2 | 2 | 3 | Medium | 1 | 1 | 2 |
| 3 | 2 | 2 | 3 | Medium | 1 | 1 | 2 |
| 3 | 2 | 2 | 3 | Medium | 1 | 1 | 2 |
| 3 | 2 | 2 | 3 | Medium | 1 | 1 | 2 |
| 3 | 2 | 2 | 3 | Medium | 1 | 1 | 2 |
| 3 | 2 | 2 | 3 | Medium | 1 | 1 | 2 |
| 3 | 2 | 2 | 3 | Medium | 1 | 1 | 2 |
| 3 | 2 | 2 | 3 | Medium | 1 | 1 | 2 |
| 3 | 2 | 2 | 3 | Medium | 1 | 1 | 2 |
| 3 | 2 | 2 | 3 | Medium | 1 | 1 | 2 |
| 3 | 2 | 2 | 3 | Medium | 1 | 1 | 2 |

| | | | | | | | |
|---|---|---|---|---|---|---|---|
| 1 | 0 | 2 | 1 | Very Low | 1 | 1 | 2 |
| 1 | 0 | 2 | 1 | Very Low | 1 | 1 | 2 |
| 1 | 0 | 2 | 1 | Very Low | 1 | 1 | 2 |
| 1 | 0 | 2 | 1 | Very Low | 1 | 1 | 2 |
| 1 | 0 | 2 | 1 | Very Low | 1 | 1 | 2 |
| 2 | 2 | 2 | 2 | Low | 1 | 1 | 0 |
| 2 | 2 | 2 | 2 | Low | 1 | 1 | 0 |
| 2 | 2 | 2 | 2 | Low | 1 | 1 | 0 |
| 2 | 2 | 2 | 2 | Low | 1 | 1 | 0 |
| 2 | 2 | 2 | 2 | Low | 1 | 1 | 0 |
| 2 | 2 | 2 | 2 | Low | 1 | 1 | 0 |
| 2 | 2 | 2 | 2 | Low | 1 | 1 | 0 |
| 2 | 2 | 2 | 2 | Low | 1 | 1 | 0 |
| 2 | 2 | 2 | 2 | Low | 1 | 1 | 0 |
| 2 | 2 | 2 | 2 | Low | 1 | 1 | 0 |
| 2 | 2 | 2 | 2 | Low | 1 | 1 | 0 |
| 2 | 2 | 2 | 2 | Low | 1 | 1 | 0 |
| 2 | 2 | 2 | 2 | Low | 1 | 1 | 0 |
| 2 | 2 | 2 | 2 | Low | 1 | 1 | 0 |
| 2 | 2 | 2 | 2 | Low | 1 | 1 | 0 |
| 2 | 2 | 2 | 2 | Low | 1 | 1 | 0 |
| 2 | 2 | 2 | 2 | Low | 1 | 1 | 0 |
| 2 | 2 | 2 | 2 | Low | 1 | 1 | 0 |
| 2 | 2 | 2 | 2 | Low | 1 | 1 | 0 |
| 2 | 2 | 2 | 2 | Low | 1 | 1 | 0 |
| 2 | 2 | 2 | 2 | Low | 1 | 1 | 0 |
| 2 | 2 | 2 | 2 | Low | 1 | 1 | 0 |
| 2 | 2 | 2 | 2 | Low | 1 | 1 | 0 |
| 2 | 2 | 2 | 2 | Low | 1 | 1 | 0 |
| 2 | 2 | 2 | 2 | Low | 1 | 1 | 0 |
| 2 | 2 | 2 | 2 | Low | 1 | 1 | 0 |
| 2 | 2 | 2 | 2 | Low | 1 | 1 | 0 |
| 2 | 2 | 2 | 2 | Low | 1 | 1 | 0 |
| 2 | 2 | 2 | 2 | Low | 1 | 1 | 0 |
| 2 | 2 | 2 | 2 | Low | 1 | 1 | 0 |
| 2 | 2 | 2 | 2 | Low | 1 | 1 | 0 |
| 2 | 2 | 2 | 2 | Low | 1 | 1 | 0 |
| 2 | 2 | 2 | 2 | Low | 1 | 1 | 0 |
| 2 | 2 | 2 | 2 | Low | 1 | 1 | 0 |
| 2 | 2 | 2 | 2 | Low | 1 | 1 | 0 |
| 2 | 2 | 2 | 2 | Low | 1 | 1 | 0 |
| 2 | 2 | 2 | 2 | Low | 1 | 1 | 0 |
| 2 | 2 | 2 | 2 | Low | 1 | 1 | 0 |
| 2 | 2 | 2 | 2 | Low | 1 | 1 | 0 |
| 2 | 2 | 2 | 2 | Low | 1 | 1 | 0 |
| 2 | 2 | 2 | 2 | Low | 1 | 1 | 0 |
| 2 | 2 | 2 | 2 | Low | 1 | 1 | 0 |
| 2 | 2 | 2 | 2 | Low | 1 | 1 | 0 |
| 2 | 2 | 2 | 2 | Low | 1 | 1 | 0 |
| 2 | 2 | 2 | 2 | Low | 1 | 1 | 0 |
| 2 | 2 | 2 | 2 | Low | 1 | 1 | 0 |
| 2 | 2 | 2 | 2 | Low | 1 | 1 | 0 |
| 2 | 2 | 2 | 2 | Low | 1 | 1 | 0 |
| 2 | 2 | 2 | 2 | Low | 1 | 1 | 0 |
| 2 | 2 | 2 | 2 | Low | 1 | 1 | 0 |
| 2 | 2 | 2 | 2 | Low | 1 | 1 | 0 |
| 2 | 2 | 2 | 2 | Low | 1 | 1 | 0 |
| 2 | 2 | 2 | 2 | Low | 1 | 1 | 0 |
| 2 | 2 | 2 | 2 | Low | 1 | 1 | 0 |

| | | | | | | | |
|---|---|---|---|---|---|---|---|
| 2 | 2 | 2 | 2 | Low | 1 | 1 | 0 |
| 2 | 2 | 2 | 2 | Low | 1 | 1 | 0 |
| 2 | 2 | 2 | 2 | Low | 1 | 1 | 0 |
| 2 | 2 | 2 | 2 | Low | 1 | 1 | 0 |
| 2 | 2 | 2 | 2 | Low | 1 | 1 | 0 |
| 2 | 2 | 2 | 2 | Low | 1 | 1 | 0 |
| 2 | 2 | 2 | 2 | Low | 1 | 1 | 0 |
| 2 | 2 | 2 | 2 | Low | 1 | 1 | 0 |
| 2 | 2 | 2 | 2 | Low | 1 | 1 | 0 |
| 2 | 2 | 2 | 2 | Low | 1 | 1 | 0 |
| 2 | 2 | 2 | 2 | Low | 1 | 1 | 0 |
| 2 | 2 | 2 | 2 | Low | 1 | 1 | 0 |
| 3 | 3 | 2 | 3 | Medium | 5 | 5 | 2 |
| 3 | 3 | 2 | 3 | Medium | 5 | 5 | 2 |
| 3 | 3 | 2 | 3 | Medium | 5 | 5 | 2 |
| 3 | 3 | 2 | 3 | Medium | 5 | 5 | 2 |
| 3 | 3 | 2 | 3 | Medium | 5 | 5 | 2 |
| 3 | 3 | 2 | 3 | Medium | 5 | 5 | 2 |
| 3 | 3 | 2 | 3 | Medium | 5 | 5 | 2 |
| 3 | 3 | 2 | 3 | Medium | 5 | 5 | 2 |
| 3 | 3 | 2 | 3 | Medium | 5 | 5 | 2 |
| 3 | 3 | 2 | 3 | Medium | 5 | 5 | 2 |
| 3 | 3 | 2 | 3 | Medium | 5 | 5 | 2 |
| 3 | 3 | 2 | 3 | Medium | 5 | 5 | 2 |
| 3 | 3 | 2 | 3 | Medium | 5 | 5 | 2 |
| 3 | 3 | 2 | 3 | Medium | 5 | 5 | 2 |
| 3 | 3 | 2 | 3 | Medium | 5 | 5 | 2 |
| 3 | 3 | 2 | 3 | Medium | 5 | 5 | 2 |
| 3 | 3 | 2 | 3 | Medium | 5 | 5 | 2 |
| 3 | 3 | 2 | 3 | Medium | 5 | 5 | 2 |
| 3 | 3 | 2 | 3 | Medium | 5 | 5 | 2 |
| 3 | 3 | 2 | 3 | Medium | 5 | 5 | 2 |
| 1 | 0 | 2 | 1 | Very Low | 1 | 1 | 0 |
| 1 | 0 | 2 | 1 | Very Low | 1 | 1 | 0 |
| 1 | 0 | 2 | 1 | Very Low | 1 | 1 | 0 |
| 1 | 0 | 2 | 1 | Very Low | 1 | 1 | 0 |
| 1 | 0 | 2 | 1 | Very Low | 1 | 1 | 0 |
| 1 | 0 | 2 | 1 | Very Low | 1 | 1 | 0 |
| 1 | 0 | 2 | 1 | Very Low | 1 | 1 | 0 |
| 1 | 0 | 2 | 1 | Very Low | 1 | 1 | 0 |
| 1 | 0 | 2 | 1 | Very Low | 1 | 1 | 0 |
| 1 | 0 | 2 | 1 | Very Low | 1 | 1 | 0 |
| 1 | 0 | 2 | 1 | Very Low | 1 | 1 | 0 |
| 1 | 0 | 2 | 1 | Very Low | 1 | 1 | 0 |
| 1 | 0 | 2 | 1 | Very Low | 1 | 1 | 0 |
| 1 | 0 | 2 | 1 | Very Low | 1 | 1 | 0 |
| 1 | 0 | 2 | 1 | Very Low | 1 | 1 | 0 |
| 1 | 0 | 2 | 1 | Very Low | 1 | 1 | 0 |
| 1 | 0 | 2 | 1 | Very Low | 1 | 1 | 0 |
| 1 | 0 | 2 | 1 | Very Low | 1 | 1 | 0 |
| 1 | 0 | 2 | 1 | Very Low | 1 | 1 | 0 |
| 1 | 0 | 2 | 1 | Very Low | 1 | 1 | 0 |
| 1 | 0 | 2 | 1 | Very Low | 1 | 1 | 0 |
| 1 | 0 | 2 | 1 | Very Low | 1 | 1 | 0 |
| 1 | 0 | 2 | 1 | Very Low | 1 | 1 | 0 |
| 1 | 0 | 2 | 1 | Very Low | 1 | 1 | 0 |
| 1 | 0 | 2 | 1 | Very Low | 1 | 1 | 0 |
| 1 | 0 | 2 | 1 | Very Low | 1 | 1 | 0 |
| 1 | 0 | 2 | 1 | Very Low | 1 | 1 | 0 |

| | | | | | | | |
|---|---|---|---|---|---|---|---|
| 1 | 0 | 2 | 1 | Very Low | 1 | 1 | 0 |
| 1 | 0 | 2 | 1 | Very Low | 1 | 1 | 0 |
| 1 | 0 | 2 | 1 | Very Low | 1 | 1 | 0 |
| 1 | 0 | 2 | 1 | Very Low | 1 | 1 | 0 |
| 1 | 0 | 2 | 1 | Very Low | 1 | 1 | 0 |
| 1 | 0 | 2 | 1 | Very Low | 1 | 1 | 0 |
| 1 | 0 | 2 | 1 | Very Low | 1 | 1 | 0 |
| 1 | 0 | 2 | 1 | Very Low | 1 | 1 | 0 |
| 1 | 0 | 2 | 1 | Very Low | 1 | 1 | 0 |
| 1 | 0 | 2 | 1 | Very Low | 1 | 1 | 0 |
| 1 | 0 | 2 | 1 | Very Low | 1 | 1 | 0 |
| 1 | 0 | 2 | 1 | Very Low | 1 | 1 | 0 |
| 1 | 0 | 2 | 1 | Very Low | 1 | 1 | 0 |
| 1 | 0 | 2 | 1 | Very Low | 1 | 1 | 0 |
| 1 | 0 | 2 | 1 | Very Low | 1 | 1 | 0 |
| 1 | 0 | 2 | 1 | Very Low | 1 | 1 | 0 |
| 1 | 0 | 2 | 1 | Very Low | 1 | 1 | 0 |
| 1 | 0 | 2 | 1 | Very Low | 1 | 1 | 0 |
| 1 | 0 | 2 | 1 | Very Low | 1 | 1 | 0 |
| 1 | 0 | 2 | 1 | Very Low | 1 | 1 | 0 |
| 1 | 0 | 2 | 1 | Very Low | 1 | 1 | 0 |
| 1 | 0 | 2 | 1 | Very Low | 1 | 1 | 0 |
| 1 | 0 | 2 | 1 | Very Low | 1 | 1 | 0 |
| 1 | 0 | 2 | 1 | Very Low | 1 | 1 | 0 |
| 1 | 0 | 2 | 1 | Very Low | 1 | 1 | 0 |
| 1 | 0 | 2 | 1 | Very Low | 1 | 1 | 0 |
| 1 | 0 | 2 | 1 | Very Low | 1 | 1 | 0 |
| 1 | 0 | 2 | 1 | Very Low | 1 | 1 | 0 |
| 1 | 0 | 2 | 1 | Very Low | 1 | 1 | 0 |
| 1 | 0 | 2 | 1 | Very Low | 1 | 1 | 0 |
| 3 | 3 | 2 | 3 | Medium | 5 | 5 | 2 |
| 3 | 3 | 2 | 3 | Medium | 5 | 5 | 2 |
| 3 | 3 | 2 | 3 | Medium | 5 | 5 | 2 |
| 2 | 2 | 2 | 2 | Low | 3 | 3 | 2 |
| 2 | 2 | 2 | 2 | Low | 3 | 3 | 2 |
| 2 | 2 | 2 | 2 | Low | 3 | 3 | 2 |
| 2 | 2 | 2 | 2 | Low | 3 | 3 | 2 |
| 2 | 2 | 2 | 2 | Low | 3 | 3 | 2 |
| 2 | 2 | 2 | 2 | Low | 3 | 3 | 2 |
| 2 | 2 | 2 | 2 | Low | 3 | 3 | 2 |
| 2 | 2 | 2 | 2 | Low | 3 | 3 | 2 |
| 2 | 2 | 2 | 2 | Low | 3 | 3 | 2 |
| 2 | 2 | 2 | 2 | Low | 3 | 3 | 2 |
| 2 | 2 | 2 | 2 | Low | 3 | 3 | 2 |
| 2 | 2 | 2 | 2 | Low | 3 | 3 | 2 |
| 2 | 2 | 2 | 2 | Low | 3 | 3 | 2 |
| 2 | 2 | 2 | 2 | Low | 3 | 3 | 2 |
| 2 | 2 | 2 | 2 | Low | 3 | 3 | 2 |
| 2 | 2 | 2 | 2 | Low | 3 | 3 | 2 |
| 2 | 2 | 2 | 2 | Low | 3 | 3 | 2 |
| 2 | 2 | 2 | 2 | Low | 3 | 3 | 2 |
| 2 | 2 | 2 | 2 | Low | 3 | 3 | 2 |
| 2 | 2 | 2 | 2 | Low | 3 | 3 | 2 |
| 2 | 2 | 2 | 2 | Low | 3 | 3 | 2 |
| 2 | 2 | 2 | 2 | Low | 3 | 5 | 2 |
| 2 | 2 | 2 | 2 | Low | 3 | 5 | 2 |
| 2 | 2 | 2 | 2 | Low | 3 | 5 | 2 |
| 2 | 2 | 2 | 2 | Low | 3 | 5 | 2 |
| 2 | 2 | 2 | 2 | Low | 3 | 5 | 2 |

| | | | | | | | |
|---|---|---|---|---|---|---|---|
| 2 | 2 | 2 | 2 | Low | 3 | 5 | 2 |
| 2 | 2 | 2 | 2 | Low | 3 | 5 | 2 |
| 2 | 2 | 2 | 2 | Low | 3 | 5 | 2 |
| 2 | 2 | 2 | 2 | Low | 3 | 5 | 2 |
| 2 | 2 | 2 | 2 | Low | 3 | 5 | 2 |
| 2 | 2 | 2 | 2 | Low | 3 | 5 | 2 |
| 2 | 2 | 2 | 2 | Low | 3 | 5 | 2 |
| 2 | 2 | 2 | 2 | Low | 3 | 5 | 2 |
| 2 | 2 | 2 | 2 | Low | 1 | 1 | 0 |
| 2 | 2 | 2 | 2 | Low | 1 | 1 | 0 |
| 2 | 2 | 2 | 2 | Low | 1 | 1 | 0 |
| 2 | 2 | 2 | 2 | Low | 1 | 1 | 0 |
| 3 | 3 | 2 | 2 | Low | 5 | 5 | 3 |
| 3 | 3 | 2 | 2 | Low | 5 | 5 | 3 |
| 3 | 3 | 2 | 2 | Low | 5 | 5 | 3 |
| 3 | 3 | 2 | 2 | Low | 5 | 5 | 3 |
| 3 | 3 | 2 | 2 | Low | 5 | 5 | 3 |
| 3 | 3 | 2 | 2 | Low | 5 | 5 | 3 |
| 3 | 3 | 2 | 2 | Low | 5 | 5 | 3 |
| 2 | 2 | 2 | 2 | Low | 1 | 1 | 3 |

| I_Biod_Pollution | I_Envi_Protected/Conserved Areas | I_Envi_Key Biodiversity Areas | I_Envi_Other Important Delineated Areas | I_Envi_Ecosystem Condition | I_Envi_Range Rarity | I_Soci_Indigenous Peoples (IPs); Local Communities (LCs) Lands | I_Soci_Resource Scarcity: Food - Water - Air |
|---|---|---|---|---|---|---|---|
| 5 | 5 | 4 | 4 | 4 | 3 | 5 | 3 |
| 5 | 5 | 4 | 4 | 4 | 3 | 5 | 3 |
| 5 | 5 | 4 | 4 | 4 | 3 | 5 | 3 |
| 5 | 5 | 4 | 4 | 4 | 3 | 5 | 3 |
| 5 | 5 | 4 | 4 | 4 | 3 | 5 | 3 |
| 5 | 5 | 4 | 4 | 4 | 3 | 5 | 3 |
| 5 | 5 | 4 | 4 | 4 | 3 | 5 | 3 |
| 5 | 3 | 2 | 2 | 2 | 1 | 3 | 1 |
| 5 | 3 | 2 | 2 | 2 | 1 | 3 | 1 |
| 5 | 3 | 2 | 2 | 2 | 1 | 3 | 1 |
| 5 | 3 | 2 | 2 | 2 | 1 | 3 | 1 |
| 5 | 3 | 2 | 2 | 2 | 1 | 3 | 1 |
| 5 | 3 | 2 | 2 | 2 | 1 | 3 | 1 |
| 5 | 3 | 2 | 2 | 2 | 1 | 3 | 1 |
| 5 | 3 | 2 | 2 | 2 | 1 | 3 | 1 |
| 5 | 3 | 2 | 2 | 2 | 1 | 3 | 1 |
| 5 | 3 | 2 | 2 | 2 | 1 | 3 | 1 |
| 5 | 3 | 2 | 2 | 2 | 1 | 3 | 1 |
| 5 | 3 | 2 | 2 | 2 | 1 | 3 | 1 |
| 5 | 3 | 2 | 2 | 2 | 1 | 3 | 1 |
| 5 | 3 | 2 | 2 | 2 | 1 | 3 | 1 |
| 5 | 3 | 2 | 2 | 2 | 1 | 3 | 1 |
| 5 | 3 | 2 | 2 | 2 | 1 | 3 | 1 |
| 5 | 3 | 2 | 2 | 2 | 1 | 3 | 1 |
| 5 | 3 | 2 | 2 | 2 | 1 | 3 | 1 |
| 5 | 3 | 2 | 2 | 2 | 1 | 3 | 1 |
| 5 | 3 | 2 | 2 | 2 | 1 | 3 | 1 |
| 5 | 3 | 2 | 2 | 2 | 1 | 3 | 1 |
| 5 | 3 | 2 | 2 | 2 | 1 | 3 | 1 |
| 5 | 3 | 2 | 2 | 2 | 1 | 3 | 1 |
| 5 | 3 | 2 | 2 | 2 | 1 | 3 | 1 |
| 5 | 3 | 2 | 2 | 2 | 1 | 3 | 1 |
| 5 | 3 | 2 | 2 | 2 | 1 | 3 | 1 |
| 5 | 3 | 2 | 2 | 2 | 1 | 3 | 1 |
| 5 | 3 | 2 | 2 | 2 | 1 | 3 | 1 |
| 5 | 3 | 2 | 2 | 2 | 1 | 3 | 1 |
| 5 | 3 | 2 | 2 | 2 | 1 | 3 | 1 |
| 5 | 3 | 2 | 2 | 2 | 1 | 3 | 1 |
| 5 | 3 | 2 | 2 | 2 | 1 | 3 | 1 |
| 5 | 3 | 2 | 2 | 2 | 1 | 3 | 1 |
| 5 | 3 | 2 | 2 | 2 | 1 | 3 | 1 |
| 5 | 3 | 2 | 2 | 2 | 1 | 3 | 1 |
| 5 | 3 | 2 | 2 | 2 | 1 | 3 | 1 |
| 5 | 3 | 2 | 2 | 2 | 1 | 3 | 1 |
| 5 | 3 | 2 | 2 | 2 | 1 | 3 | 1 |
| 5 | 3 | 2 | 2 | 2 | 1 | 3 | 1 |
| 5 | 3 | 2 | 2 | 2 | 1 | 3 | 1 |
| 5 | 3 | 2 | 2 | 2 | 1 | 3 | 1 |
| 5 | 3 | 2 | 2 | 2 | 1 | 3 | 1 |
| 5 | 3 | 2 | 2 | 2 | 1 | 3 | 1 |
| 5 | 3 | 2 | 2 | 2 | 1 | 3 | 1 |
| 5 | 3 | 2 | 2 | 2 | 1 | 3 | 1 |
| 5 | 3 | 2 | 2 | 2 | 1 | 3 | 1 |
| 5 | 3 | 2 | 2 | 2 | 1 | 3 | 1 |

| | | | | | | | |
|---|---|---|---|---|---|---|---|
| 5 | 3 | 2 | 2 | 2 | 1 | 3 | 1 |
| 5 | 3 | 2 | 2 | 2 | 1 | 3 | 1 |
| 5 | 3 | 2 | 2 | 2 | 1 | 3 | 1 |
| 5 | 3 | 2 | 2 | 2 | 1 | 3 | 1 |
| 5 | 3 | 2 | 2 | 2 | 1 | 3 | 1 |
| 5 | 3 | 2 | 2 | 2 | 1 | 3 | 1 |
| 5 | 3 | 2 | 2 | 2 | 1 | 3 | 1 |
| 5 | 3 | 2 | 2 | 2 | 1 | 3 | 1 |
| 5 | 3 | 2 | 2 | 2 | 1 | 3 | 1 |
| 5 | 3 | 2 | 2 | 2 | 1 | 3 | 1 |
| 5 | 3 | 2 | 2 | 2 | 1 | 3 | 1 |
| 5 | 3 | 2 | 2 | 2 | 1 | 3 | 1 |
| 5 | 3 | 2 | 2 | 2 | 1 | 3 | 1 |
| 5 | 3 | 2 | 2 | 2 | 1 | 3 | 1 |
| 5 | 3 | 2 | 2 | 2 | 1 | 3 | 1 |
| 5 | 3 | 2 | 2 | 2 | 1 | 3 | 1 |
| 5 | 3 | 2 | 2 | 2 | 1 | 3 | 1 |
| 5 | 3 | 2 | 2 | 2 | 1 | 3 | 1 |
| 5 | 3 | 2 | 2 | 2 | 1 | 3 | 1 |
| 5 | 3 | 2 | 2 | 2 | 1 | 3 | 1 |
| 5 | 3 | 2 | 2 | 2 | 1 | 3 | 1 |
| 5 | 3 | 2 | 2 | 2 | 1 | 3 | 1 |
| 5 | 3 | 2 | 2 | 2 | 1 | 3 | 1 |
| 5 | 3 | 2 | 2 | 2 | 1 | 3 | 1 |
| 5 | 3 | 2 | 2 | 2 | 1 | 3 | 1 |
| 5 | 3 | 2 | 2 | 2 | 1 | 3 | 1 |
| 5 | 3 | 2 | 2 | 2 | 1 | 3 | 1 |
| 5 | 3 | 2 | 2 | 2 | 1 | 3 | 1 |
| 5 | 3 | 2 | 2 | 2 | 1 | 3 | 1 |
| 5 | 3 | 2 | 2 | 2 | 1 | 3 | 1 |
| 5 | 3 | 2 | 2 | 2 | 1 | 3 | 1 |
| 5 | 3 | 2 | 2 | 2 | 1 | 3 | 1 |
| 5 | 5 | 4 | 4 | 4 | 3 | 3 | 1 |
| 5 | 5 | 4 | 4 | 4 | 3 | 3 | 1 |
| 5 | 5 | 4 | 4 | 4 | 3 | 3 | 1 |
| 5 | 5 | 4 | 4 | 4 | 3 | 3 | 1 |
| 5 | 5 | 4 | 4 | 4 | 3 | 3 | 1 |
| 5 | 5 | 4 | 4 | 4 | 3 | 3 | 1 |
| 5 | 5 | 4 | 4 | 4 | 3 | 3 | 1 |
| 5 | 5 | 4 | 4 | 4 | 3 | 3 | 1 |
| 5 | 5 | 4 | 4 | 4 | 3 | 3 | 1 |
| 4 | 4 | 3 | 2 | 2 | 3 | 3 | 0 |
| 4 | 4 | 3 | 2 | 2 | 3 | 3 | 0 |
| 4 | 4 | 3 | 2 | 2 | 3 | 3 | 0 |
| 4 | 4 | 3 | 2 | 2 | 3 | 3 | 0 |
| 4 | 4 | 3 | 2 | 2 | 3 | 3 | 0 |
| 4 | 4 | 3 | 2 | 2 | 3 | 3 | 0 |
| 4 | 4 | 3 | 2 | 2 | 3 | 3 | 0 |
| 4 | 4 | 3 | 2 | 2 | 3 | 3 | 0 |
| 4 | 4 | 3 | 2 | 2 | 3 | 3 | 0 |
| 4 | 4 | 3 | 2 | 2 | 3 | 3 | 0 |
| 4 | 4 | 3 | 2 | 2 | 3 | 3 | 0 |
| 4 | 4 | 3 | 2 | 2 | 3 | 3 | 0 |
| 4 | 4 | 3 | 2 | 2 | 3 | 3 | 0 |
| 4 | 4 | 3 | 2 | 2 | 3 | 3 | 0 |
| 4 | 4 | 3 | 4 | 4 | 3 | 5 | 1 |
| 5 | 3 | 2 | 2 | 2 | 1 | 3 | 1 |
| 5 | 3 | 2 | 2 | 2 | 1 | 3 | 1 |
| 5 | 3 | 2 | 2 | 2 | 1 | 3 | 1 |

| | | | | | | | |
|---|---|---|---|---|---|---|---|
| 5 | 3 | 2 | 2 | 2 | 1 | 3 | 1 |
| 5 | 3 | 2 | 2 | 2 | 1 | 3 | 1 |
| 5 | 3 | 2 | 2 | 2 | 1 | 3 | 1 |
| 5 | 3 | 2 | 2 | 2 | 1 | 3 | 1 |
| 5 | 3 | 2 | 2 | 2 | 1 | 3 | 1 |
| 5 | 3 | 2 | 2 | 2 | 1 | 3 | 1 |
| 5 | 3 | 2 | 2 | 2 | 1 | 3 | 1 |
| 5 | 3 | 2 | 2 | 2 | 1 | 3 | 1 |
| 5 | 3 | 2 | 2 | 2 | 1 | 3 | 1 |
| 5 | 3 | 2 | 2 | 2 | 1 | 3 | 1 |
| 5 | 3 | 2 | 2 | 2 | 1 | 3 | 1 |
| 5 | 3 | 2 | 2 | 2 | 1 | 3 | 1 |
| 5 | 3 | 2 | 2 | 2 | 1 | 3 | 1 |
| 5 | 3 | 2 | 2 | 2 | 1 | 3 | 1 |
| 5 | 3 | 2 | 2 | 2 | 1 | 3 | 1 |
| 5 | 3 | 2 | 2 | 2 | 1 | 3 | 1 |
| 5 | 3 | 2 | 2 | 2 | 1 | 3 | 1 |
| 5 | 3 | 2 | 2 | 2 | 1 | 3 | 1 |
| 5 | 3 | 2 | 2 | 2 | 1 | 3 | 1 |
| 5 | 3 | 2 | 2 | 2 | 1 | 3 | 1 |
| 5 | 3 | 2 | 2 | 2 | 1 | 3 | 1 |
| 5 | 3 | 2 | 2 | 2 | 1 | 3 | 1 |
| 5 | 3 | 2 | 2 | 2 | 1 | 3 | 1 |
| 5 | 3 | 2 | 2 | 2 | 1 | 3 | 1 |
| 5 | 3 | 2 | 2 | 2 | 1 | 3 | 1 |
| 5 | 3 | 2 | 2 | 2 | 1 | 3 | 1 |
| 5 | 3 | 2 | 2 | 2 | 1 | 3 | 1 |
| 5 | 3 | 2 | 2 | 2 | 1 | 3 | 1 |
| 5 | 3 | 2 | 2 | 2 | 1 | 3 | 1 |
| 5 | 3 | 2 | 2 | 2 | 1 | 3 | 1 |
| 5 | 3 | 2 | 2 | 2 | 1 | 3 | 1 |
| 5 | 3 | 2 | 2 | 2 | 1 | 3 | 1 |
| 5 | 3 | 2 | 2 | 2 | 1 | 3 | 1 |
| 5 | 3 | 2 | 2 | 2 | 1 | 3 | 1 |
| 5 | 3 | 2 | 2 | 2 | 1 | 3 | 1 |
| 5 | 3 | 2 | 2 | 2 | 1 | 3 | 1 |
| 5 | 3 | 2 | 2 | 2 | 1 | 3 | 1 |
| 5 | 3 | 2 | 2 | 2 | 1 | 3 | 1 |
| 5 | 3 | 2 | 2 | 2 | 1 | 3 | 1 |
| 5 | 3 | 2 | 2 | 2 | 1 | 3 | 1 |
| 5 | 3 | 2 | 2 | 2 | 1 | 3 | 1 |
| 5 | 3 | 2 | 2 | 2 | 1 | 3 | 1 |
| 5 | 3 | 2 | 2 | 2 | 1 | 3 | 1 |
| 5 | 3 | 2 | 2 | 2 | 1 | 3 | 1 |
| 5 | 3 | 2 | 2 | 2 | 1 | 3 | 1 |
| 5 | 3 | 2 | 2 | 2 | 1 | 3 | 1 |
| 5 | 3 | 2 | 2 | 2 | 1 | 3 | 1 |
| 5 | 3 | 2 | 2 | 2 | 1 | 3 | 1 |
| 5 | 3 | 2 | 2 | 2 | 1 | 3 | 1 |
| 5 | 3 | 2 | 2 | 2 | 1 | 3 | 1 |
| 5 | 3 | 2 | 2 | 2 | 1 | 3 | 1 |
| 5 | 3 | 2 | 2 | 2 | 1 | 3 | 1 |
| 5 | 3 | 2 | 2 | 2 | 1 | 3 | 1 |
| 5 | 3 | 2 | 2 | 2 | 1 | 3 | 1 |
| 5 | 3 | 2 | 2 | 2 | 1 | 3 | 1 |
| 5 | 3 | 2 | 2 | 2 | 1 | 3 | 1 |
| 5 | 3 | 2 | 2 | 2 | 1 | 3 | 1 |
| 5 | 3 | 2 | 2 | 2 | 1 | 3 | 1 |
| 5 | 3 | 2 | 2 | 2 | 1 | 3 | 1 |

| | | | | | | | |
|---|---|---|---|---|---|---|---|
| 5 | 3 | 2 | 2 | 2 | 1 | 3 | 1 |
| 5 | 3 | 2 | 2 | 2 | 1 | 3 | 1 |
| 5 | 3 | 2 | 2 | 2 | 1 | 3 | 1 |
| 5 | 3 | 2 | 2 | 2 | 1 | 3 | 1 |
| 5 | 3 | 2 | 2 | 2 | 1 | 3 | 1 |
| 5 | 3 | 2 | 2 | 2 | 1 | 3 | 1 |
| 5 | 3 | 2 | 2 | 2 | 1 | 3 | 1 |
| 5 | 3 | 2 | 2 | 2 | 1 | 3 | 1 |
| 5 | 3 | 2 | 2 | 2 | 1 | 3 | 1 |
| 5 | 3 | 2 | 2 | 2 | 1 | 3 | 1 |
| 5 | 3 | 2 | 2 | 2 | 1 | 3 | 1 |
| 5 | 3 | 2 | 2 | 2 | 1 | 3 | 1 |
| 5 | 3 | 2 | 2 | 2 | 1 | 3 | 1 |
| 5 | 3 | 2 | 2 | 2 | 1 | 3 | 1 |
| 5 | 3 | 2 | 2 | 2 | 1 | 3 | 1 |
| 5 | 3 | 2 | 2 | 2 | 1 | 3 | 1 |
| 5 | 3 | 2 | 2 | 2 | 1 | 3 | 1 |
| 5 | 3 | 2 | 2 | 2 | 1 | 3 | 1 |
| 5 | 3 | 2 | 2 | 2 | 1 | 3 | 1 |
| 5 | 3 | 2 | 2 | 2 | 1 | 3 | 1 |
| 5 | 3 | 2 | 2 | 2 | 1 | 3 | 1 |
| 5 | 3 | 2 | 2 | 2 | 1 | 3 | 1 |
| 5 | 3 | 2 | 2 | 2 | 1 | 3 | 1 |
| 5 | 3 | 2 | 2 | 2 | 1 | 3 | 1 |
| 5 | 3 | 2 | 2 | 2 | 1 | 3 | 1 |
| 5 | 3 | 2 | 2 | 2 | 1 | 3 | 1 |
| 5 | 3 | 2 | 2 | 2 | 1 | 3 | 1 |
| 5 | 3 | 2 | 2 | 2 | 1 | 3 | 1 |
| 5 | 3 | 2 | 2 | 2 | 1 | 3 | 1 |
| 5 | 3 | 2 | 2 | 2 | 1 | 3 | 1 |
| 5 | 3 | 2 | 2 | 2 | 1 | 3 | 1 |
| 5 | 3 | 2 | 2 | 2 | 1 | 3 | 1 |
| 5 | 3 | 2 | 2 | 2 | 1 | 3 | 1 |
| 5 | 3 | 2 | 2 | 2 | 1 | 3 | 1 |
| 5 | 3 | 2 | 2 | 2 | 1 | 3 | 1 |
| 5 | 3 | 2 | 2 | 2 | 1 | 3 | 1 |
| 5 | 3 | 2 | 2 | 2 | 1 | 3 | 1 |
| 5 | 3 | 2 | 2 | 2 | 1 | 3 | 1 |
| 5 | 3 | 2 | 2 | 2 | 1 | 3 | 1 |
| 5 | 3 | 2 | 2 | 2 | 1 | 3 | 1 |
| 4 | 3 | 2 | 2 | 2 | 1 | 3 | 2 |
| 4 | 3 | 2 | 2 | 2 | 1 | 3 | 2 |
| 4 | 3 | 2 | 2 | 2 | 1 | 3 | 2 |
| 4 | 3 | 2 | 2 | 2 | 1 | 3 | 2 |
| 4 | 3 | 2 | 2 | 2 | 1 | 3 | 2 |
| 4 | 3 | 2 | 2 | 2 | 1 | 3 | 2 |
| 4 | 3 | 2 | 2 | 2 | 1 | 3 | 2 |
| 4 | 3 | 2 | 2 | 2 | 1 | 3 | 2 |
| 4 | 3 | 2 | 2 | 2 | 1 | 3 | 2 |
| 4 | 3 | 2 | 2 | 2 | 1 | 3 | 2 |
| 4 | 3 | 2 | 2 | 2 | 1 | 3 | 2 |
| 4 | 3 | 2 | 2 | 2 | 1 | 3 | 2 |
| 4 | 3 | 2 | 2 | 2 | 1 | 3 | 2 |
| 4 | 3 | 2 | 2 | 2 | 1 | 3 | 2 |
| 4 | 3 | 2 | 2 | 2 | 1 | 3 | 2 |
| 4 | 3 | 2 | 2 | 2 | 1 | 3 | 2 |
| 4 | 3 | 2 | 2 | 2 | 1 | 3 | 2 |
| 4 | 3 | 2 | 2 | 2 | 1 | 3 | 2 |
| 4 | 3 | 2 | 2 | 2 | 1 | 3 | 2 |

| | | | | | | | |
|---|---|---|---|---|---|---|---|
| 4 | 1 | 1 | 1 | 1 | 0 | 1 | 1 |
| 4 | 1 | 1 | 1 | 1 | 0 | 1 | 0 |
| 4 | 1 | 1 | 1 | 1 | 0 | 1 | 0 |
| 4 | 1 | 1 | 1 | 1 | 0 | 1 | 0 |
| 4 | 1 | 1 | 1 | 1 | 0 | 1 | 0 |
| 5 | 3 | 2 | 2 | 2 | 1 | 3 | 1 |
| 5 | 3 | 2 | 2 | 2 | 1 | 3 | 1 |
| 5 | 3 | 2 | 2 | 2 | 1 | 3 | 1 |
| 5 | 3 | 2 | 2 | 2 | 1 | 3 | 1 |
| 5 | 3 | 2 | 2 | 2 | 1 | 3 | 1 |
| 5 | 3 | 2 | 2 | 2 | 1 | 3 | 1 |
| 5 | 3 | 2 | 2 | 2 | 1 | 3 | 1 |
| 5 | 3 | 2 | 2 | 2 | 1 | 3 | 1 |
| 5 | 3 | 2 | 2 | 2 | 1 | 3 | 1 |
| 5 | 3 | 2 | 2 | 2 | 1 | 3 | 1 |
| 5 | 3 | 2 | 2 | 2 | 1 | 3 | 1 |
| 5 | 3 | 2 | 2 | 2 | 1 | 3 | 1 |
| 5 | 3 | 2 | 2 | 2 | 1 | 3 | 1 |
| 5 | 3 | 2 | 2 | 2 | 1 | 3 | 1 |
| 5 | 3 | 2 | 2 | 2 | 1 | 3 | 1 |
| 5 | 3 | 2 | 2 | 2 | 1 | 3 | 1 |
| 5 | 3 | 2 | 2 | 2 | 1 | 3 | 1 |
| 5 | 3 | 2 | 2 | 2 | 1 | 3 | 1 |
| 5 | 3 | 2 | 2 | 2 | 1 | 3 | 1 |
| 5 | 3 | 2 | 2 | 2 | 1 | 3 | 1 |
| 5 | 3 | 2 | 2 | 2 | 1 | 3 | 1 |
| 5 | 3 | 2 | 2 | 2 | 1 | 3 | 1 |
| 5 | 3 | 2 | 2 | 2 | 1 | 3 | 1 |
| 5 | 3 | 2 | 2 | 2 | 1 | 3 | 1 |
| 5 | 3 | 2 | 2 | 2 | 1 | 3 | 1 |
| 5 | 3 | 2 | 2 | 2 | 1 | 3 | 1 |
| 5 | 3 | 2 | 2 | 2 | 1 | 3 | 1 |
| 5 | 3 | 2 | 2 | 2 | 1 | 3 | 1 |
| 5 | 3 | 2 | 2 | 2 | 1 | 3 | 1 |
| 5 | 3 | 2 | 2 | 2 | 1 | 3 | 1 |
| 5 | 3 | 2 | 2 | 2 | 1 | 3 | 1 |
| 5 | 3 | 2 | 2 | 2 | 1 | 3 | 1 |
| 5 | 3 | 2 | 2 | 2 | 1 | 3 | 1 |
| 5 | 3 | 2 | 2 | 2 | 1 | 3 | 1 |
| 5 | 3 | 2 | 2 | 2 | 1 | 3 | 1 |
| 5 | 3 | 2 | 2 | 2 | 1 | 3 | 1 |
| 5 | 3 | 2 | 2 | 2 | 1 | 3 | 1 |
| 5 | 3 | 2 | 2 | 2 | 1 | 3 | 1 |
| 5 | 3 | 2 | 2 | 2 | 1 | 3 | 1 |
| 5 | 3 | 2 | 2 | 2 | 1 | 3 | 1 |
| 5 | 3 | 2 | 2 | 2 | 1 | 3 | 1 |
| 5 | 3 | 2 | 2 | 2 | 1 | 3 | 1 |
| 5 | 3 | 2 | 2 | 2 | 1 | 3 | 1 |
| 5 | 3 | 2 | 2 | 2 | 1 | 3 | 1 |
| 5 | 3 | 2 | 2 | 2 | 1 | 3 | 1 |
| 5 | 3 | 2 | 2 | 2 | 1 | 3 | 1 |
| 5 | 3 | 2 | 2 | 2 | 1 | 3 | 1 |
| 5 | 3 | 2 | 2 | 2 | 1 | 3 | 1 |
| 5 | 3 | 2 | 2 | 2 | 1 | 3 | 1 |
| 5 | 3 | 2 | 2 | 2 | 1 | 3 | 1 |
| 5 | 3 | 2 | 2 | 2 | 1 | 3 | 1 |
| 5 | 3 | 2 | 2 | 2 | 1 | 3 | 1 |
| 5 | 3 | 2 | 2 | 2 | 1 | 3 | 1 |
| 5 | 3 | 2 | 2 | 2 | 1 | 3 | 1 |

| | | | | | | | |
|---|---|---|---|---|---|---|---|
| 5 | 3 | 2 | 2 | 2 | 1 | 3 | 1 |
| 5 | 3 | 2 | 2 | 2 | 1 | 3 | 1 |
| 5 | 3 | 2 | 2 | 2 | 1 | 3 | 1 |
| 5 | 3 | 2 | 2 | 2 | 1 | 3 | 1 |
| 5 | 3 | 2 | 2 | 2 | 1 | 3 | 1 |
| 5 | 3 | 2 | 2 | 2 | 1 | 3 | 1 |
| 5 | 3 | 2 | 2 | 2 | 1 | 3 | 1 |
| 5 | 3 | 2 | 2 | 2 | 1 | 3 | 1 |
| 5 | 3 | 2 | 2 | 2 | 1 | 3 | 1 |
| 5 | 3 | 2 | 2 | 2 | 1 | 3 | 1 |
| 5 | 3 | 2 | 2 | 2 | 1 | 3 | 1 |
| 5 | 3 | 2 | 2 | 2 | 1 | 3 | 1 |
| 5 | 5 | 4 | 4 | 4 | 3 | 5 | 1 |
| 5 | 5 | 4 | 4 | 4 | 3 | 5 | 1 |
| 5 | 5 | 4 | 4 | 4 | 3 | 5 | 1 |
| 5 | 5 | 4 | 4 | 4 | 3 | 5 | 1 |
| 5 | 5 | 4 | 4 | 4 | 3 | 5 | 1 |
| 5 | 5 | 4 | 4 | 4 | 3 | 5 | 1 |
| 5 | 5 | 4 | 4 | 4 | 3 | 5 | 1 |
| 5 | 5 | 4 | 4 | 4 | 3 | 5 | 1 |
| 5 | 5 | 4 | 4 | 4 | 3 | 5 | 1 |
| 5 | 5 | 4 | 4 | 4 | 3 | 5 | 1 |
| 5 | 5 | 4 | 4 | 4 | 3 | 5 | 1 |
| 5 | 5 | 4 | 4 | 4 | 3 | 5 | 2 |
| 5 | 5 | 4 | 4 | 4 | 3 | 5 | 2 |
| 5 | 5 | 4 | 4 | 4 | 3 | 5 | 2 |
| 5 | 5 | 4 | 4 | 4 | 3 | 5 | 2 |
| 5 | 5 | 4 | 4 | 4 | 3 | 5 | 2 |
| 5 | 5 | 4 | 4 | 4 | 3 | 5 | 2 |
| 5 | 5 | 4 | 4 | 4 | 3 | 5 | 2 |
| 5 | 5 | 4 | 4 | 4 | 3 | 5 | 2 |
| 5 | 5 | 4 | 4 | 4 | 3 | 5 | 2 |
| 2 | 1 | 1 | 1 | 1 | 0 | 1 | 0 |
| 2 | 1 | 1 | 1 | 1 | 0 | 1 | 0 |
| 2 | 1 | 1 | 1 | 1 | 0 | 1 | 0 |
| 2 | 1 | 1 | 1 | 1 | 0 | 1 | 0 |
| 2 | 1 | 1 | 1 | 1 | 0 | 1 | 0 |
| 2 | 1 | 1 | 1 | 1 | 0 | 1 | 0 |
| 2 | 1 | 1 | 1 | 1 | 0 | 1 | 0 |
| 2 | 1 | 1 | 1 | 1 | 0 | 1 | 0 |
| 2 | 1 | 1 | 1 | 1 | 0 | 1 | 0 |
| 2 | 1 | 1 | 1 | 1 | 0 | 1 | 0 |
| 2 | 1 | 1 | 1 | 1 | 0 | 1 | 0 |
| 2 | 1 | 1 | 1 | 1 | 0 | 1 | 0 |
| 2 | 1 | 1 | 1 | 1 | 0 | 1 | 0 |
| 2 | 1 | 1 | 1 | 1 | 0 | 1 | 0 |
| 2 | 1 | 1 | 1 | 1 | 0 | 1 | 0 |
| 2 | 1 | 1 | 1 | 1 | 0 | 1 | 0 |
| 2 | 1 | 1 | 1 | 1 | 0 | 1 | 0 |
| 2 | 1 | 1 | 1 | 1 | 0 | 1 | 0 |
| 2 | 1 | 1 | 1 | 1 | 0 | 1 | 0 |
| 2 | 1 | 1 | 1 | 1 | 0 | 1 | 0 |
| 2 | 1 | 1 | 1 | 1 | 0 | 1 | 0 |
| 2 | 1 | 1 | 1 | 1 | 0 | 1 | 0 |
| 2 | 1 | 1 | 1 | 1 | 0 | 1 | 0 |
| 2 | 1 | 1 | 1 | 1 | 0 | 1 | 0 |
| 2 | 1 | 1 | 1 | 1 | 0 | 1 | 0 |
| 2 | 1 | 1 | 1 | 1 | 0 | 1 | 0 |
| 2 | 1 | 1 | 1 | 1 | 0 | 1 | 0 |

| | | | | | | | |
|---|---|---|---|---|---|---|---|
| 2 | 1 | 1 | 1 | 1 | 0 | 1 | 0 |
| 2 | 1 | 1 | 1 | 1 | 0 | 1 | 0 |
| 2 | 1 | 1 | 1 | 1 | 0 | 1 | 0 |
| 2 | 1 | 1 | 1 | 1 | 0 | 1 | 0 |
| 2 | 1 | 1 | 1 | 1 | 0 | 1 | 0 |
| 2 | 1 | 1 | 1 | 1 | 0 | 1 | 0 |
| 2 | 1 | 1 | 1 | 1 | 0 | 1 | 0 |
| 2 | 1 | 1 | 1 | 1 | 0 | 1 | 0 |
| 2 | 1 | 1 | 1 | 1 | 0 | 1 | 0 |
| 2 | 1 | 1 | 1 | 1 | 0 | 1 | 0 |
| 2 | 1 | 1 | 1 | 1 | 0 | 1 | 0 |
| 2 | 1 | 1 | 1 | 1 | 0 | 1 | 0 |
| 2 | 1 | 1 | 1 | 1 | 0 | 1 | 0 |
| 2 | 1 | 1 | 1 | 1 | 0 | 1 | 0 |
| 2 | 1 | 1 | 1 | 1 | 0 | 1 | 0 |
| 2 | 1 | 1 | 1 | 1 | 0 | 1 | 0 |
| 2 | 1 | 1 | 1 | 1 | 0 | 1 | 0 |
| 2 | 1 | 1 | 1 | 1 | 0 | 1 | 0 |
| 2 | 1 | 1 | 1 | 1 | 0 | 1 | 0 |
| 2 | 1 | 1 | 1 | 1 | 0 | 1 | 0 |
| 2 | 1 | 1 | 1 | 1 | 0 | 1 | 0 |
| 2 | 1 | 1 | 1 | 1 | 0 | 1 | 0 |
| 2 | 1 | 1 | 1 | 1 | 0 | 1 | 0 |
| 2 | 1 | 1 | 1 | 1 | 0 | 1 | 0 |
| 2 | 1 | 1 | 1 | 1 | 0 | 1 | 0 |
| 2 | 1 | 1 | 1 | 1 | 0 | 1 | 0 |
| 2 | 1 | 1 | 1 | 1 | 0 | 1 | 0 |
| 2 | 1 | 1 | 1 | 1 | 0 | 1 | 0 |
| 2 | 1 | 1 | 1 | 1 | 0 | 1 | 0 |
| 2 | 1 | 1 | 1 | 1 | 0 | 1 | 0 |
| 5 | 5 | 4 | 4 | 4 | 3 | 5 | 2 |
| 5 | 5 | 4 | 4 | 4 | 3 | 5 | 2 |
| 5 | 5 | 4 | 4 | 4 | 3 | 5 | 2 |
| 1 | 3 | 3 | 3 | 3 | 2 | 3 | 1 |
| 1 | 3 | 3 | 3 | 3 | 2 | 3 | 1 |
| 1 | 3 | 3 | 3 | 3 | 2 | 3 | 1 |
| 1 | 3 | 3 | 3 | 3 | 2 | 3 | 1 |
| 1 | 3 | 3 | 3 | 3 | 2 | 3 | 1 |
| 1 | 3 | 3 | 3 | 3 | 2 | 3 | 1 |
| 1 | 3 | 3 | 3 | 3 | 2 | 3 | 1 |
| 1 | 3 | 3 | 3 | 3 | 2 | 3 | 1 |
| 1 | 3 | 3 | 3 | 3 | 2 | 3 | 1 |
| 1 | 3 | 3 | 3 | 3 | 2 | 3 | 1 |
| 1 | 3 | 3 | 3 | 3 | 2 | 3 | 1 |
| 1 | 3 | 3 | 3 | 3 | 2 | 3 | 1 |
| 1 | 3 | 3 | 3 | 3 | 2 | 3 | 1 |
| 1 | 3 | 3 | 3 | 3 | 2 | 3 | 1 |
| 1 | 3 | 3 | 3 | 3 | 2 | 3 | 1 |
| 1 | 3 | 3 | 3 | 3 | 2 | 3 | 1 |
| 1 | 3 | 3 | 3 | 3 | 2 | 3 | 1 |
| 1 | 3 | 3 | 3 | 3 | 2 | 3 | 1 |
| 1 | 3 | 3 | 3 | 3 | 2 | 3 | 1 |
| 1 | 3 | 3 | 3 | 3 | 2 | 3 | 1 |
| 1 | 3 | 3 | 3 | 3 | 2 | 3 | 1 |
| 2 | 3 | 2 | 2 | 2 | 2 | 3 | 0 |
| 2 | 3 | 2 | 2 | 2 | 2 | 3 | 0 |
| 2 | 3 | 2 | 2 | 2 | 2 | 3 | 0 |
| 2 | 3 | 2 | 2 | 2 | 2 | 3 | 0 |
| 2 | 3 | 2 | 2 | 2 | 2 | 3 | 0 |

| | | | | | | | |
|---|---|---|---|---|---|---|---|
| 2 | 3 | 2 | 2 | 2 | 2 | 3 | 0 |
| 2 | 3 | 2 | 2 | 2 | 2 | 3 | 0 |
| 2 | 3 | 2 | 2 | 2 | 2 | 3 | 0 |
| 2 | 3 | 2 | 2 | 2 | 2 | 3 | 0 |
| 2 | 3 | 2 | 2 | 2 | 2 | 3 | 0 |
| 2 | 3 | 2 | 2 | 2 | 2 | 3 | 0 |
| 2 | 3 | 2 | 2 | 2 | 2 | 3 | 0 |
| 2 | 3 | 2 | 2 | 2 | 2 | 3 | 0 |
| 5 | 3 | 2 | 2 | 2 | 1 | 3 | 2 |
| 5 | 3 | 2 | 2 | 2 | 1 | 3 | 2 |
| 5 | 3 | 2 | 2 | 2 | 1 | 3 | 2 |
| 5 | 3 | 2 | 2 | 2 | 1 | 3 | 2 |
| 4 | 5 | 4 | 4 | 4 | 2 | 5 | 1 |
| 4 | 5 | 4 | 4 | 4 | 2 | 5 | 1 |
| 4 | 5 | 4 | 4 | 4 | 2 | 5 | 1 |
| 4 | 5 | 4 | 4 | 4 | 2 | 5 | 1 |
| 4 | 5 | 4 | 4 | 4 | 2 | 5 | 1 |
| 4 | 5 | 4 | 4 | 4 | 2 | 5 | 1 |
| 4 | 5 | 4 | 4 | 4 | 2 | 5 | 1 |
| 2 | 3 | 2 | 2 | 2 | 2 | 3 | 1 |

| I_Soci_Labor/Human Rights | I_Soci_Financial Inequality | Impact_Median | Reputational risk |
|---|---|---|---|
| 4 | 2 | 4 | High |
| 4 | 2 | 4 | High |
| 4 | 2 | 4 | High |
| 4 | 2 | 4 | High |
| 4 | 2 | 4 | High |
| 4 | 2 | 4 | High |
| 4 | 2 | 4 | High |
| 2 | 2 | 2 | Low |
| 2 | 2 | 2 | Low |
| 2 | 2 | 2 | Low |
| 2 | 2 | 2 | Low |
| 2 | 2 | 2 | Low |
| 2 | 2 | 2 | Low |
| 2 | 2 | 2 | Low |
| 2 | 2 | 2 | Low |
| 2 | 2 | 2 | Low |
| 2 | 2 | 2 | Low |
| 2 | 2 | 2 | Low |
| 2 | 2 | 2 | Low |
| 2 | 2 | 2 | Low |
| 2 | 2 | 2 | Low |
| 2 | 2 | 2 | Low |
| 2 | 2 | 2 | Low |
| 2 | 2 | 2 | Low |
| 2 | 2 | 2 | Low |
| 2 | 2 | 2 | Low |
| 2 | 2 | 2 | Low |
| 2 | 2 | 2 | Low |
| 2 | 2 | 2 | Low |
| 2 | 2 | 2 | Low |
| 2 | 2 | 2 | Low |
| 2 | 2 | 2 | Low |
| 2 | 2 | 2 | Low |
| 2 | 2 | 2 | Low |
| 2 | 2 | 2 | Low |
| 2 | 2 | 2 | Low |
| 2 | 2 | 2 | Low |
| 2 | 2 | 2 | Low |
| 2 | 2 | 2 | Low |
| 2 | 2 | 2 | Low |
| 2 | 2 | 2 | Low |
| 2 | 2 | 2 | Low |
| 2 | 2 | 2 | Low |
| 2 | 2 | 2 | Low |
| 2 | 2 | 2 | Low |
| 2 | 2 | 2 | Low |
| 2 | 2 | 2 | Low |
| 2 | 2 | 2 | Low |
| 2 | 2 | 2 | Low |
| 2 | 2 | 2 | Low |
| 2 | 2 | 2 | Low |
| 2 | 2 | 2 | Low |
| 2 | 2 | 2 | Low |
| 2 | 2 | 2 | Low |

| | | | |
|---|---|---|---|
| 2 | 2 | 2 | Low |
| 2 | 2 | 2 | Low |
| 2 | 2 | 2 | Low |
| 2 | 2 | 2 | Low |
| 2 | 2 | 2 | Low |
| 2 | 2 | 2 | Low |
| 2 | 2 | 2 | Low |
| 2 | 2 | 2 | Low |
| 2 | 2 | 2 | Low |
| 2 | 2 | 2 | Low |
| 2 | 2 | 2 | Low |
| 2 | 2 | 2 | Low |
| 2 | 2 | 2 | Low |
| 2 | 2 | 2 | Low |
| 2 | 2 | 2 | Low |
| 2 | 2 | 2 | Low |
| 2 | 2 | 2 | Low |
| 2 | 2 | 2 | Low |
| 2 | 2 | 2 | Low |
| 2 | 2 | 2 | Low |
| 2 | 2 | 2 | Low |
| 2 | 2 | 2 | Low |
| 2 | 2 | 2 | Low |
| 2 | 2 | 2 | Low |
| 2 | 2 | 2 | Low |
| 2 | 2 | 2 | Low |
| 2 | 2 | 2 | Low |
| 2 | 2 | 2 | Low |
| 2 | 2 | 2 | Low |
| 2 | 2 | 2 | Low |
| 2 | 2 | 2 | Low |
| 2 | 2 | 2 | Low |
| 2 | 2 | 3 | Medium |
| 2 | 2 | 3 | Medium |
| 2 | 2 | 3 | Medium |
| 2 | 2 | 3 | Medium |
| 2 | 2 | 3 | Medium |
| 2 | 2 | 3 | Medium |
| 2 | 2 | 3 | Medium |
| 2 | 2 | 3 | Medium |
| 2 | 2 | 3 | Medium |
| 2 | 2 | 2 | Low |
| 2 | 2 | 2 | Low |
| 2 | 2 | 2 | Low |
| 2 | 2 | 2 | Low |
| 2 | 2 | 2 | Low |
| 2 | 2 | 2 | Low |
| 2 | 2 | 2 | Low |
| 2 | 2 | 2 | Low |
| 2 | 2 | 2 | Low |
| 2 | 2 | 2 | Low |
| 2 | 2 | 2 | Low |
| 2 | 2 | 2 | Low |
| 2 | 2 | 2 | Low |
| 2 | 2 | 2 | Low |
| 2 | 2 | 4 | High |
| 2 | 2 | 2 | Low |
| 2 | 2 | 2 | Low |
| 2 | 2 | 2 | Low |

| | | | |
|---|---|---|---|
| 2 | 2 | 2 | Low |
| 2 | 2 | 2 | Low |
| 2 | 2 | 2 | Low |
| 2 | 2 | 2 | Low |
| 2 | 2 | 2 | Low |
| 2 | 2 | 2 | Low |
| 2 | 2 | 2 | Low |
| 2 | 2 | 2 | Low |
| 2 | 2 | 2 | Low |
| 2 | 2 | 2 | Low |
| 2 | 2 | 2 | Low |
| 2 | 2 | 2 | Low |
| 2 | 2 | 2 | Low |
| 2 | 2 | 2 | Low |
| 2 | 2 | 2 | Low |
| 2 | 2 | 2 | Low |
| 2 | 2 | 2 | Low |
| 2 | 2 | 2 | Low |
| 2 | 2 | 2 | Low |
| 2 | 2 | 2 | Low |
| 2 | 2 | 2 | Low |
| 2 | 2 | 2 | Low |
| 2 | 2 | 2 | Low |
| 2 | 2 | 2 | Low |
| 2 | 2 | 2 | Low |
| 2 | 2 | 2 | Low |
| 2 | 2 | 2 | Low |
| 2 | 2 | 2 | Low |
| 2 | 2 | 2 | Low |
| 2 | 2 | 2 | Low |
| 2 | 2 | 2 | Low |
| 2 | 2 | 2 | Low |
| 2 | 2 | 2 | Low |
| 2 | 2 | 2 | Low |
| 2 | 2 | 2 | Low |
| 2 | 2 | 2 | Low |
| 2 | 2 | 2 | Low |
| 2 | 2 | 2 | Low |
| 2 | 2 | 2 | Low |
| 2 | 2 | 2 | Low |
| 2 | 2 | 2 | Low |
| 2 | 2 | 2 | Low |
| 2 | 2 | 2 | Low |
| 2 | 2 | 2 | Low |
| 2 | 2 | 2 | Low |
| 2 | 2 | 2 | Low |
| 2 | 2 | 2 | Low |
| 2 | 2 | 2 | Low |
| 2 | 2 | 2 | Low |
| 2 | 2 | 2 | Low |
| 2 | 2 | 2 | Low |
| 2 | 2 | 2 | Low |
| 2 | 2 | 2 | Low |
| 2 | 2 | 2 | Low |
| 2 | 2 | 2 | Low |
| 2 | 2 | 2 | Low |
| 2 | 2 | 2 | Low |
| 2 | 2 | 2 | Low |
| 2 | 2 | 2 | Low |

| | | | |
|---|---|---|---|
| 2 | 2 | 2 | Low |
| 2 | 2 | 2 | Low |
| 2 | 2 | 2 | Low |
| 2 | 2 | 2 | Low |
| 2 | 2 | 2 | Low |
| 2 | 2 | 2 | Low |
| 2 | 2 | 2 | Low |
| 2 | 2 | 2 | Low |
| 2 | 2 | 2 | Low |
| 2 | 2 | 2 | Low |
| 2 | 2 | 2 | Low |
| 2 | 2 | 2 | Low |
| 2 | 2 | 2 | Low |
| 2 | 2 | 2 | Low |
| 2 | 2 | 2 | Low |
| 2 | 2 | 2 | Low |
| 2 | 2 | 2 | Low |
| 2 | 2 | 2 | Low |
| 2 | 2 | 2 | Low |
| 2 | 2 | 2 | Low |
| 2 | 2 | 2 | Low |
| 2 | 2 | 2 | Low |
| 2 | 2 | 2 | Low |
| 2 | 2 | 2 | Low |
| 2 | 2 | 2 | Low |
| 2 | 2 | 2 | Low |
| 2 | 2 | 2 | Low |
| 2 | 2 | 2 | Low |
| 2 | 2 | 2 | Low |
| 2 | 2 | 2 | Low |
| 2 | 2 | 2 | Low |
| 2 | 2 | 2 | Low |
| 2 | 2 | 2 | Low |
| 2 | 2 | 2 | Low |
| 2 | 2 | 2 | Low |
| 2 | 2 | 2 | Low |
| 2 | 2 | 2 | Low |
| 2 | 2 | 2 | Low |
| 2 | 2 | 2 | Low |
| 2 | 2 | 2 | Low |
| 2 | 2 | 2 | Low |
| 2 | 2 | 2 | Low |
| 2 | 2 | 2 | Low |
| 2 | 2 | 2 | Low |
| 2 | 2 | 2 | Low |
| 2 | 2 | 2 | Low |
| 2 | 2 | 2 | Low |
| 2 | 2 | 2 | Low |
| 2 | 2 | 2 | Low |
| 2 | 2 | 2 | Low |
| 2 | 2 | 2 | Low |
| 2 | 2 | 2 | Low |
| 2 | 2 | 2 | Low |
| 2 | 2 | 2 | Low |
| 2 | 2 | 2 | Low |
| 2 | 2 | 2 | Low |
| 2 | 2 | 2 | Low |
| 2 | 2 | 2 | Low |
| 2 | 2 | 2 | Low |

| | | | |
|---|---|---|---|
| 2 | 2 | 1 | Very Low |
| 2 | 2 | 1 | Very Low |
| 2 | 2 | 1 | Very Low |
| 2 | 2 | 1 | Very Low |
| 2 | 2 | 1 | Very Low |
| 2 | 2 | 2 | Low |
| 2 | 2 | 2 | Low |
| 2 | 2 | 2 | Low |
| 2 | 2 | 2 | Low |
| 2 | 2 | 2 | Low |
| 2 | 2 | 2 | Low |
| 2 | 2 | 2 | Low |
| 2 | 2 | 2 | Low |
| 2 | 2 | 2 | Low |
| 2 | 2 | 2 | Low |
| 2 | 2 | 2 | Low |
| 2 | 2 | 2 | Low |
| 2 | 2 | 2 | Low |
| 2 | 2 | 2 | Low |
| 2 | 2 | 2 | Low |
| 2 | 2 | 2 | Low |
| 2 | 2 | 2 | Low |
| 2 | 2 | 2 | Low |
| 2 | 2 | 2 | Low |
| 2 | 2 | 2 | Low |
| 2 | 2 | 2 | Low |
| 2 | 2 | 2 | Low |
| 2 | 2 | 2 | Low |
| 2 | 2 | 2 | Low |
| 2 | 2 | 2 | Low |
| 2 | 2 | 2 | Low |
| 2 | 2 | 2 | Low |
| 2 | 2 | 2 | Low |
| 2 | 2 | 2 | Low |
| 2 | 2 | 2 | Low |
| 2 | 2 | 2 | Low |
| 2 | 2 | 2 | Low |
| 2 | 2 | 2 | Low |
| 2 | 2 | 2 | Low |
| 2 | 2 | 2 | Low |
| 2 | 2 | 2 | Low |
| 2 | 2 | 2 | Low |
| 2 | 2 | 2 | Low |
| 2 | 2 | 2 | Low |
| 2 | 2 | 2 | Low |
| 2 | 2 | 2 | Low |
| 2 | 2 | 2 | Low |
| 2 | 2 | 2 | Low |
| 2 | 2 | 2 | Low |
| 2 | 2 | 2 | Low |
| 2 | 2 | 2 | Low |
| 2 | 2 | 2 | Low |
| 2 | 2 | 2 | Low |
| 2 | 2 | 2 | Low |
| 2 | 2 | 2 | Low |
| 2 | 2 | 2 | Low |
| 2 | 2 | 2 | Low |
| 2 | 2 | 2 | Low |
| 2 | 2 | 2 | Low |

| | | | |
|---|---|---|---|
| 2 | 2 | 2 | Low |
| 2 | 2 | 2 | Low |
| 2 | 2 | 2 | Low |
| 2 | 2 | 2 | Low |
| 2 | 2 | 2 | Low |
| 2 | 2 | 2 | Low |
| 2 | 2 | 2 | Low |
| 2 | 2 | 2 | Low |
| 2 | 2 | 2 | Low |
| 2 | 2 | 2 | Low |
| 2 | 2 | 2 | Low |
| 2 | 2 | 2 | Low |
| 4 | 2 | 4 | High |
| 4 | 2 | 4 | High |
| 4 | 2 | 4 | High |
| 4 | 2 | 4 | High |
| 4 | 2 | 4 | High |
| 4 | 2 | 4 | High |
| 4 | 2 | 4 | High |
| 4 | 2 | 4 | High |
| 4 | 2 | 4 | High |
| 4 | 2 | 4 | High |
| 4 | 2 | 4 | High |
| 4 | 2 | 4 | High |
| 4 | 2 | 4 | High |
| 4 | 2 | 4 | High |
| 4 | 2 | 4 | High |
| 4 | 2 | 4 | High |
| 4 | 2 | 4 | High |
| 4 | 2 | 4 | High |
| 4 | 2 | 4 | High |
| 4 | 2 | 4 | High |
| 2 | 2 | 1 | Very Low |
| 2 | 2 | 1 | Very Low |
| 2 | 2 | 1 | Very Low |
| 2 | 2 | 1 | Very Low |
| 2 | 2 | 1 | Very Low |
| 2 | 2 | 1 | Very Low |
| 2 | 2 | 1 | Very Low |
| 2 | 2 | 1 | Very Low |
| 2 | 2 | 1 | Very Low |
| 2 | 2 | 1 | Very Low |
| 2 | 2 | 1 | Very Low |
| 2 | 2 | 1 | Very Low |
| 2 | 2 | 1 | Very Low |
| 2 | 2 | 1 | Very Low |
| 2 | 2 | 1 | Very Low |
| 2 | 2 | 1 | Very Low |
| 2 | 2 | 1 | Very Low |
| 2 | 2 | 1 | Very Low |
| 2 | 2 | 1 | Very Low |
| 2 | 2 | 1 | Very Low |
| 2 | 2 | 1 | Very Low |
| 2 | 2 | 1 | Very Low |
| 2 | 2 | 1 | Very Low |
| 2 | 2 | 1 | Very Low |
| 2 | 2 | 1 | Very Low |
| 2 | 2 | 1 | Very Low |
| 2 | 2 | 1 | Very Low |

| | | | |
|---|---|---|---|
| 2 | 2 | 1 | Very Low |
| 2 | 2 | 1 | Very Low |
| 2 | 2 | 1 | Very Low |
| 2 | 2 | 1 | Very Low |
| 2 | 2 | 1 | Very Low |
| 2 | 2 | 1 | Very Low |
| 2 | 2 | 1 | Very Low |
| 2 | 2 | 1 | Very Low |
| 2 | 2 | 1 | Very Low |
| 2 | 2 | 1 | Very Low |
| 2 | 2 | 1 | Very Low |
| 2 | 2 | 1 | Very Low |
| 2 | 2 | 1 | Very Low |
| 2 | 2 | 1 | Very Low |
| 2 | 2 | 1 | Very Low |
| 2 | 2 | 1 | Very Low |
| 2 | 2 | 1 | Very Low |
| 2 | 2 | 1 | Very Low |
| 2 | 2 | 1 | Very Low |
| 2 | 2 | 1 | Very Low |
| 2 | 2 | 1 | Very Low |
| 2 | 2 | 1 | Very Low |
| 2 | 2 | 1 | Very Low |
| 2 | 2 | 1 | Very Low |
| 2 | 2 | 1 | Very Low |
| 2 | 2 | 1 | Very Low |
| 2 | 2 | 1 | Very Low |
| 2 | 2 | 1 | Very Low |
| 2 | 2 | 1 | Very Low |
| 2 | 2 | 1 | Very Low |
| 4 | 2 | 4 | High |
| 4 | 2 | 4 | High |
| 4 | 2 | 4 | High |
| 3 | 2 | 3 | Medium |
| 3 | 2 | 3 | Medium |
| 3 | 2 | 3 | Medium |
| 3 | 2 | 3 | Medium |
| 3 | 2 | 3 | Medium |
| 3 | 2 | 3 | Medium |
| 3 | 2 | 3 | Medium |
| 3 | 2 | 3 | Medium |
| 3 | 2 | 3 | Medium |
| 3 | 2 | 3 | Medium |
| 3 | 2 | 3 | Medium |
| 3 | 2 | 3 | Medium |
| 3 | 2 | 3 | Medium |
| 3 | 2 | 3 | Medium |
| 3 | 2 | 3 | Medium |
| 3 | 2 | 3 | Medium |
| 3 | 2 | 3 | Medium |
| 3 | 2 | 3 | Medium |
| 3 | 2 | 3 | Medium |
| 3 | 2 | 3 | Medium |
| 3 | 2 | 3 | Medium |
| 2 | 2 | 2 | Low |
| 2 | 2 | 2 | Low |
| 2 | 2 | 2 | Low |
| 2 | 2 | 2 | Low |
| 2 | 2 | 2 | Low |

| | | | |
|---|---|---|---|
| 2 | 2 | 2 | Low |
| 2 | 2 | 2 | Low |
| 2 | 2 | 2 | Low |
| 2 | 2 | 2 | Low |
| 2 | 2 | 2 | Low |
| 2 | 2 | 2 | Low |
| 2 | 2 | 2 | Low |
| 2 | 2 | 2 | Low |
| 4 | 2 | 2 | Low |
| 4 | 2 | 2 | Low |
| 4 | 2 | 2 | Low |
| 4 | 2 | 2 | Low |
| 2 | 2 | 4 | High |
| 2 | 2 | 4 | High |
| 2 | 2 | 4 | High |
| 2 | 2 | 4 | High |
| 2 | 2 | 4 | High |
| 2 | 2 | 4 | High |
| 2 | 2 | 4 | High |
| 2 | 2 | 2 | Low |

| Index | Company Name | Code |
|---|---|---|
| SSE 180 | INNER MONGOLIA YILI INDUSTRIAL GROUF | 600887 |
| SSE 180 | TONGWEI CO.,LTD | 600438 |
| SSE 180 | Ecovacs Robotics Co., Ltd. | 603486 |
| SSE 180 | Haier Smart Home Co., Ltd. | 600690 |
| SSE 180 | Ningbo Deye Technology Co., Ltd. | 605117 |
| SSE 180 | AECC AVIATION POWER CO,LTD | 600893 |
| SSE 180 | Anhui Jianghuai Automobile Group Corp.,L | 600418 |
| SSE 180 | AVIC HEAVY MACHINERY CO.,LTD. | 600765 |
| SSE 180 | AVIC SHENYANG AIRCRAFT COMPANY LIMI | 600760 |
| SSE 180 | Bethel Auto Safety Systems Co.,Ltd | 603596 |
| SSE 180 | Great Wall Motor Company Limited | 601633 |
| SSE 180 | GUANGZHOU AUTOMOBILE GROUP CO.,LT | 601238 |
| SSE 180 | SAIC Motor Corporation Limited | 600104 |
| SSE 180 | China Shipbuilding Industry Company Limit | 601989 |
| SSE 180 | China Spacesat Co.,Ltd. | 600118 |
| SSE 180 | CRRC Corporation Limited | 601766 |
| SSE 180 | DONGFANG ELECTRIC CORPORATION LIMI | 600875 |
| SSE 180 | FUYAO GLASS INDUSTRY GROUP CO., LTD. | 600660 |
| SSE 180 | HOYUAN Green Energy Co.,Ltd | 603185 |
| SSE 180 | jiangsu hengli hydraulic co.Ltd | 601100 |
| SSE 180 | Ming Yang Smart Energy Group Limited | 601615 |
| SSE 180 | Ningbo Tuopu Group Co.,Ltd. | 601689 |
| SSE 180 | SANY HEAVY INDUSTRY CO.,LTD | 600031 |
| SSE 180 | TBEA CO.,LTD. | 600089 |
| SSE 180 | Wingtech Technology Co.,Ltd. | 600745 |
| SSE 180 | ZHEJIANG CHINT ELECTRICS CO.,LTD. | 601877 |
| SSE 180 | ZHEJIANG HUAYOU COBALT CO., LTD. | 603799 |
| SSE 180 | Xinjiang Daqo New Energy Co.,Ltd. | 688303 |
| SSE 180 | JIANGSU PACIFIC QUARTZ CO.,LTD. | 603688 |
| SSE 180 | NINGBO ORIENT WIRES & CABLES CO., LTD | 603606 |
| SSE 180 | SHANDONG HUALU-HENGSHENG CHEMIC/ | 600426 |
| SSE 180 | Wanhua Chemical Group Co.,Ltd. | 600309 |
| SSE 180 | China Jushi Co.,Ltd. | 600176 |
| SSE 180 | China National Chemical Engineering CO.,L | 601117 |
| SSE 180 | China Northern Rare Earth (Group) High-Te | 600111 |
| SSE 180 | Hoshine Silicon Industry Co., Ltd. | 603260 |
| SSE 180 | Jinduicheng Molybdenum Co.,Ltd | 601958 |
| SSE 180 | YUNNAN YUNTIANHUA CO.,LTD | 600096 |
| SSE 180 | ZHEJIANG JUHUA CO.,LTD. | 600160 |
| SSE 180 | Anhui Conch Cement Company Limited | 600585 |
| SSE 180 | Baoshan Iron & Steel Co., Ltd. | 600019 |
| SSE 180 | Inner Mongolia BaoTou Steel Union Co.,Ltc | 600010 |
| SSE 180 | Oppein Home Group Inc. | 603833 |
| SSE 180 | China National Nuclear Power Co., Ltd. | 601985 |
| SSE 180 | China Shenhua Energy Company Limited | 601088 |
| SSE 180 | CNOOC Limited | 600938 |
| SSE 180 | Huadian Power International Corporation I | 600027 |

| | | |
|---|---|---|
| SSE 180 | Huaneng Power International, INC. | 600011 |
| SSE 180 | SHAANXI COAL INDUSTRY COMPANY LIMIT | 601225 |
| SSE 180 | Yankuang Energy Group Company Limited | 600188 |
| SSE 180 | China Energy Engineering Corporation Limi | 601868 |
| SSE 180 | China Three Gorges Renewables (Group) C | 600905 |
| SSE 180 | LONGi Green Energy Technology Co., Ltd. | 601012 |
| SSE 180 | Shanghai Aiko Solar Energy Co., Ltd. | 600732 |
| SSE 180 | GD POWER DEVELOPMENT CO., LTD | 600795 |
| SSE 180 | Power Construction Corporation of China,L | 601669 |
| SSE 180 | SHANGHAI ELECTRIC POWER COMPANY LI | 600021 |
| SSE 180 | China Yangtze Power Co.,ltd. | 600900 |
| SSE 180 | Advanced Micro-Fabrication Equipment In | 688012 |
| SSE 180 | Cambricon Technologies Corporation Limit | 688256 |
| SSE 180 | GigaDevice Semiconductor Inc. | 603986 |
| SSE 180 | GoodWe Technologies Co.,Ltd. | 688390 |
| SSE 180 | Hygon Information Technology Co., Ltd. | 688041 |
| SSE 180 | Jinko Solar Co., Ltd. | 688223 |
| SSE 180 | Montage Technology Co., Ltd. | 688008 |
| SSE 180 | Pylon Technologies Co., Ltd. | 688063 |
| SSE 180 | Semiconductor Manufacturing Internationa | 688981 |
| SSE 180 | STARPOWER SEMICONDUCTOR LTD. | 603290 |
| SSE 180 | Transsion Holdings LTD.CO | 688036 |
| SSE 180 | Trina Solar Co., Ltd. | 688599 |
| SSE 180 | Western Superconducting Technologies Co | 688122 |
| SSE 180 | Will Semiconductor CO., Ltd. Shanghai | 603501 |
| SSE 180 | Dawning Information Industry Co., Ltd. | 603019 |
| SSE 180 | HANGZHOU FIRST APPLIED MATERIAL CO., | 603806 |
| SSE 180 | Hangzhou Lion Microelectronics Co.,Ltd. | 605358 |
| SSE 180 | Hangzhou Silan Microelectronics Co.,Ltd. | 600460 |
| SSE 180 | JCET Group Co., Ltd. | 600584 |
| SSE 180 | Shanghai Putailai New Energy Technology ( | 603659 |
| SSE 180 | JUEWEI FOOD CO., LTD. | 603517 |
| SSE 180 | ANGEL YEAST CO.,LTD | 600298 |
| SSE 180 | Anhui Kouzi Distillery Co., Ltd. | 603589 |
| SSE 180 | ANJOY FOODS GROUP CO.,LTD. | 603345 |
| SSE 180 | Chongqing Brewery Company Limited | 600132 |
| SSE 180 | Foshan Haitian Flavouring and Food Compa | 603288 |
| SSE 180 | SHANXI XINGHUACUN FEN WINE FACTORY | 600809 |
| SSE 180 | TSINGTAO BREWERY COMPANY LIMITED | 600600 |
| SSE 180 | Jiangsu King’s Luck Brewery Joint – Stock C | 603369 |
| SSE 180 | JONJEE HI-TECH INDUSTRIAL & COMMERCI | 600872 |
| SSE 180 | KWEICHOW MOUTAI CO.,LTD. | 600519 |

| | | |
|---|---|---|
| SSE 180 | SICHUAN SWELLFUN CO.,LTD | 600779 |
| SSE 180 | China Tourism Group Duty Free Corporatio | 601888 |
| SSE 180 | Shanghai Jin Jiang International Hotels Co., | 600754 |
| SSE 180 | WANGFUJING GROUP CO., LTD. | 600859 |
| SSE 180 | BEIJING TONG REN TANG CO., LTD | 600085 |
| SSE 180 | Beijing Wantai Biological Pharmacy Enterpi | 603392 |
| SSE 180 | Bloomage Biotechnology Corporation Limit | 688363 |
| SSE 180 | Jiangsu Hengrui Pharmaceuticals Co.,Ltd. | 600276 |
| SSE 180 | Shanghai Fosun Pharmaceutical (Group) Cc | 600196 |
| SSE 180 | Shanghai Junshi Biosciences Co., Ltd. | 688180 |
| SSE 180 | Shanghai Pharmaceuticals Holding Co., Ltd. | 601607 |
| SSE 180 | Shanghai United Imaging Healthcare Co., Lt | 688271 |
| SSE 180 | TOPCHOICE MEDICAL CO.INC | 600763 |
| SSE 180 | Yifeng Pharmacy Chain Co., Ltd. | 603939 |
| SSE 180 | Zhejiang Huahai Pharmaceutical Co.,LTD. | 600521 |
| SSE 180 | CHINA MEHECO GROUP CO., LTD. | 600056 |
| SSE 180 | Guangzhou Baiyunshan Pharmaceutical Ho | 600332 |
| SSE 180 | Proya Cosmetics Co.,LTD | 603605 |
| SSE 180 | WuXi AppTec Co., Ltd. | 603259 |
| SSE 180 | ZHANGZHOU PIENTZEHUANG PHARMACEU | 600436 |
| SSE 180 | China Communications Construction Co.,Lt | 601800 |
| SSE 180 | China Railway Construction Corporation Lir | 601186 |
| SSE 180 | China Railway Group Limited | 601390 |
| SSE 180 | China State Construction Engineering Corp | 601668 |
| SSE 180 | Metallurgical Corporation of China Ltd. | 601618 |
| SSE 180 | Poly Developments and Holdings Group Co | 600048 |
| SSE 180 | Seazen Holdings Co.,Ltd. | 601155 |
| SSE 180 | SHANGHAI ZHANGJIANG HI-TECH PARK DE | 600895 |
| SSE 180 | Ningxia Baofeng Energy Group Co.,Ltd. | 600989 |
| SSE 180 | ALUMINUM CORPORATION OF CHINA LIMI | 601600 |
| SSE 180 | Chifeng Jilong Gold Mining Co.,Ltd. | 600988 |
| SSE 180 | SHANDONG GOLD MINING CO.,LTD. | 600547 |
| SSE 180 | ZHONGJIN GOLD CORPORATION LIMITED | 600489 |
| SSE 180 | Zijin Mining Group Company Limited | 601899 |
| SSE 180 | CMOC Group Limited | 603993 |
| SSE 180 | Beijing Kingsoft Office Software, Inc. | 688111 |
| SSE 180 | NEW CHINA LIFE INSURANCE COMPANY LT | 601336 |
| SSE 180 | ORIENT SECURITIES COMPANY LIMITED | 600958 |
| SSE 180 | SHANGHAI PUDONG DEVELOPMENT BANK | 600000 |
| SSE 180 | SOOCHOW SECURITIES CO.,LTD. | 601555 |

| | | |
|---|---|---|
| SSE 180 | Yonyou Network Technology Co., Ltd. | 600588 |
| SSE 180 | ZheJiang China Commodities City Group Co | 600415 |
| SSE 180 | Zheshang Securities Co.,Ltd. | 601878 |
| SSE 180 | 360 Security Technology Inc. | 601360 |
| SSE 180 | AGRICULTURAL BANK OF CHINA LIMITED | 601288 |
| SSE 180 | BANK OF BEIJING CO.,LTD. | 601169 |
| SSE 180 | BANK OF CHINA LIMITED | 601988 |
| SSE 180 | BANK OF COMMUNICATIONS CO.,LTD. | 601328 |
| SSE 180 | BANK OF JIANGSU CO., LTD. | 600919 |
| SSE 180 | BOC International (China) Co., Ltd. | 601696 |
| SSE 180 | CAPITAL SECURITIES CORPORATION LIMITE | 601136 |
| SSE 180 | CHINA CITIC BANK CORPORATION LIMITED | 601998 |
| SSE 180 | CHINA CONSTRUCTION BANK CORPORATIO | 601939 |
| SSE 180 | CHINA EVERBRIGHT BANK COMPANY LIMIT | 601818 |
| SSE 180 | INDUSTRIAL AND COMMERCIAL BANK OF C | 601398 |
| SSE 180 | INDUSTRIAL BANK CO.,LTD. | 601166 |
| SSE 180 | PING AN INSURANCE （GROUP） COMPAN | 601318 |
| SSE 180 | POSTAL SAVINGS BANK OF CHINA, LTD. | 601658 |
| SSE 180 | The People's Insurance Company (Group) o | 601319 |
| SSE 180 | China Galaxy Securities Co., Ltd. | 601881 |
| SSE 180 | China Industrial Securities Co.,Ltd | 601377 |
| SSE 180 | China International Capital Corporation Lin | 601995 |
| SSE 180 | CHINA LIFE INSURANCE COMPANY LIMITED | 601628 |
| SSE 180 | China Merchants Bank Co., Ltd. | 600036 |
| SSE 180 | China Merchants Securities Co.,Ltd. | 600999 |
| SSE 180 | CHINA MINSHENG BANK | 600016 |
| SSE 180 | China Pacific Insurance (Group) Co., Ltd. | 601601 |
| SSE 180 | CHINA SECURITIES CO., LTD. | 601066 |
| SSE 180 | CINDA SECURITIES CO.,LTD | 601059 |
| SSE 180 | CITIC Securities Company Limited | 600030 |
| SSE 180 | Everbright Securities Company Limited | 601788 |
| SSE 180 | Guotai Junan Securities Co. Ltd. | 601211 |
| SSE 180 | HAITONG Securities Company Limited | 600837 |
| SSE 180 | HUATAI SECURITIES CO.,LTD | 601688 |
| SSE 180 | HUNDSUN TECHNOLOGIES INC. | 600570 |
| SSE 180 | NARI Technology Co., Ltd. | 600406 |
| SSE 180 | China Petroleum & Chemical Corporation | 600028 |
| SSE 180 | COSCO SHIPPING Energy Transportation Co | 600026 |
| SSE 180 | PetroChina Company Limited | 601857 |
| SSE 180 | CHINA MOBILE LIMITED | 600941 |

| | | |
|---|---|---|
| SSE 180 | China Telecom Corporation Limited | 601728 |
| SSE 180 | China Satellite Communications Co., Ltd. | 601698 |
| SSE 180 | CHINA UNITED NETWORK COMMUNICATIC | 600050 |
| SSE 180 | Foxconn Industrial Internet Co., Ltd. | 601138 |
| SSE 180 | HENGLI PETROCHEMICAL CO.,LTD. | 600346 |
| SSE 180 | Beijing-Shanghai High Speed Railway Co.,Lt | 601816 |
| SSE 180 | Air China Limited | 601111 |
| SSE 180 | China CSSC Holdings Limited | 600150 |
| SSE 180 | Shanghai International Airport Co., Ltd. | 600009 |
| SSE 180 | CHINA MERCHANTS ENERGY SHIPPING CO. | 601872 |
| SSE 180 | COSCO SHIPPING Holdings Co., Ltd. | 601919 |

| Sector | Water Scarcity | Forest Productivity | Limited Wild Flora & Fauna | Limited Marine Fish |
|---|---|---|---|---|
| Agriculture (animal products) | 5 | 0 | 1 | 0 |
| Agriculture (plant products) | 5 | 0 | 1 | 0 |
| Appliances & General Goods Manufacturing | 4 | 0 | 0 | 0 |
| Appliances & General Goods Manufacturing | 4 | 0 | 0 | 0 |
| Appliances & General Goods Manufacturing | 4 | 0 | 0 | 0 |
| Automotive, Electrical Equipment & Machinery | 4 | 0 | 3 | 0 |
| Automotive, Electrical Equipment & Machinery | 4 | 0 | 3 | 0 |
| Automotive, Electrical Equipment & Machinery | 4 | 0 | 3 | 0 |
| Automotive, Electrical Equipment & Machinery | 4 | 0 | 3 | 0 |
| Automotive, Electrical Equipment & Machinery | 4 | 0 | 3 | 0 |
| Automotive, Electrical Equipment & Machinery | 4 | 0 | 3 | 0 |
| Automotive, Electrical Equipment & Machinery | 4 | 0 | 3 | 0 |
| Automotive, Electrical Equipment & Machinery | 4 | 0 | 3 | 0 |
| Automotive, Electrical Equipment & Machinery | 4 | 0 | 3 | 0 |
| Automotive, Electrical Equipment & Machinery | 4 | 0 | 3 | 0 |
| Automotive, Electrical Equipment & Machinery | 4 | 0 | 3 | 0 |
| Automotive, Electrical Equipment & Machinery | 4 | 0 | 3 | 0 |
| Automotive, Electrical Equipment & Machinery | 4 | 0 | 3 | 0 |
| Automotive, Electrical Equipment & Machinery | 4 | 0 | 3 | 0 |
| Automotive, Electrical Equipment & Machinery | 4 | 0 | 3 | 0 |
| Automotive, Electrical Equipment & Machinery | 4 | 0 | 3 | 0 |
| Automotive, Electrical Equipment & Machinery | 4 | 0 | 3 | 0 |
| Automotive, Electrical Equipment & Machinery | 4 | 0 | 3 | 0 |
| Automotive, Electrical Equipment & Machinery | 4 | 0 | 3 | 0 |
| Automotive, Electrical Equipment & Machinery | 4 | 0 | 3 | 0 |
| Automotive, Electrical Equipment & Machinery | 4 | 0 | 3 | 0 |
| Automotive, Electrical Equipment & Machinery | 4 | 0 | 3 | 0 |
| Chemicals & Other Materials Production | 4 | 0 | 1 | 0 |
| Chemicals & Other Materials Production | 4 | 0 | 1 | 0 |
| Chemicals & Other Materials Production | 4 | 0 | 1 | 0 |
| Chemicals & Other Materials Production | 4 | 0 | 1 | 0 |
| Chemicals & Other Materials Production | 4 | 0 | 1 | 0 |
| Chemicals & Other Materials Production | 4 | 0 | 1 | 0 |
| Chemicals & Other Materials Production | 4 | 0 | 1 | 0 |
| Chemicals & Other Materials Production | 4 | 0 | 1 | 0 |
| Chemicals & Other Materials Production | 4 | 0 | 1 | 0 |
| Chemicals & Other Materials Production | 4 | 0 | 1 | 0 |
| Chemicals & Other Materials Production | 4 | 0 | 1 | 0 |
| Chemicals & Other Materials Production | 4 | 0 | 1 | 0 |
| Construction Materials | 5 | 4 | 1 | 0 |
| Construction Materials | 5 | 4 | 1 | 0 |
| Construction Materials | 5 | 4 | 1 | 0 |
| Construction Materials | 5 | 4 | 1 | 0 |
| Electric Energy Production - Combustion (Bion | 5 | 4 | 0 | 0 |
| Electric Energy Production - Combustion (Bion | 5 | 4 | 0 | 0 |
| Electric Energy Production - Combustion (Bion | 5 | 4 | 0 | 0 |
| Electric Energy Production - Combustion (Bion | 5 | 4 | 0 | 0 |

| | | | | |
|---|---|---|---|---|
| Electric Energy Production - Combustion (Bion | 5 | 4 | 0 | 0 |
| Electric Energy Production - Combustion (Bion | 5 | 4 | 0 | 0 |
| Electric Energy Production - Combustion (Bion | 5 | 4 | 0 | 0 |
| Electric Energy Production - Solar, Wind | 3 | 0 | 0 | 0 |
| Electric Energy Production - Solar, Wind | 3 | 0 | 0 | 0 |
| Electric Energy Production - Solar, Wind | 3 | 0 | 0 | 0 |
| Electric Energy Production - Solar, Wind | 3 | 0 | 0 | 0 |
| Electric Energy Production - Solar, Wind | 3 | 0 | 0 | 0 |
| Electric Energy Production - Solar, Wind | 3 | 0 | 0 | 0 |
| Electric Energy Production - Solar, Wind | 3 | 0 | 0 | 0 |
| Electric Energy Production – Hydropower | 5 | 0 | 0 | 0 |
| Electronics & Semiconductor Manufacturing | 4 | 0 | 0 | 0 |
| Electronics & Semiconductor Manufacturing | 4 | 0 | 0 | 0 |
| Electronics & Semiconductor Manufacturing | 4 | 0 | 0 | 0 |
| Electronics & Semiconductor Manufacturing | 4 | 0 | 0 | 0 |
| Electronics & Semiconductor Manufacturing | 4 | 0 | 0 | 0 |
| Electronics & Semiconductor Manufacturing | 4 | 0 | 0 | 0 |
| Electronics & Semiconductor Manufacturing | 4 | 0 | 0 | 0 |
| Electronics & Semiconductor Manufacturing | 4 | 0 | 0 | 0 |
| Electronics & Semiconductor Manufacturing | 4 | 0 | 0 | 0 |
| Electronics & Semiconductor Manufacturing | 4 | 0 | 0 | 0 |
| Electronics & Semiconductor Manufacturing | 4 | 0 | 0 | 0 |
| Electronics & Semiconductor Manufacturing | 4 | 0 | 0 | 0 |
| Electronics & Semiconductor Manufacturing | 4 | 0 | 0 | 0 |
| Electronics & Semiconductor Manufacturing | 4 | 0 | 0 | 0 |
| Electronics & Semiconductor Manufacturing | 4 | 0 | 0 | 0 |
| Electronics & Semiconductor Manufacturing | 4 | 0 | 0 | 0 |
| Electronics & Semiconductor Manufacturing | 4 | 0 | 0 | 0 |
| Electronics & Semiconductor Manufacturing | 4 | 0 | 0 | 0 |
| Electronics & Semiconductor Manufacturing | 4 | 0 | 0 | 0 |
| Food & Beverage Production | 5 | 0 | 3 | 0 |
| Food & Beverage Production | 5 | 0 | 3 | 0 |
| Food & Beverage Production | 5 | 0 | 3 | 0 |
| Food & Beverage Production | 5 | 0 | 3 | 0 |
| Food & Beverage Production | 5 | 0 | 3 | 0 |
| Food & Beverage Production | 5 | 0 | 3 | 0 |
| Food & Beverage Production | 5 | 0 | 3 | 0 |
| Food & Beverage Production | 5 | 0 | 3 | 0 |
| Food & Beverage Production | 5 | 0 | 3 | 0 |
| Food & Beverage Production | 5 | 0 | 3 | 0 |
| Food & Beverage Production | 5 | 0 | 3 | 0 |

| | | | | |
|---|---|---|---|---|
| Food & Beverage Production | 5 | 0 | 3 | 0 |
| General or Speciality Retailing | 2 | 0 | 0 | 0 |
| General or Speciality Retailing | 2 | 0 | 0 | 0 |
| General or Speciality Retailing | 2 | 0 | 0 | 0 |
| Health Care, Pharmaceuticals and Biotechnolo | 4 | 0 | 3 | 0 |
| Health Care, Pharmaceuticals and Biotechnolo | 4 | 0 | 3 | 0 |
| Health Care, Pharmaceuticals and Biotechnolo | 4 | 0 | 3 | 0 |
| Health Care, Pharmaceuticals and Biotechnolo | 4 | 0 | 3 | 0 |
| Health Care, Pharmaceuticals and Biotechnolo | 4 | 0 | 3 | 0 |
| Health Care, Pharmaceuticals and Biotechnolo | 4 | 0 | 3 | 0 |
| Health Care, Pharmaceuticals and Biotechnolo | 4 | 0 | 3 | 0 |
| Health Care, Pharmaceuticals and Biotechnolo | 4 | 0 | 3 | 0 |
| Health Care, Pharmaceuticals and Biotechnolo | 4 | 0 | 3 | 0 |
| Health Care, Pharmaceuticals and Biotechnolo | 4 | 0 | 3 | 0 |
| Health Care, Pharmaceuticals and Biotechnolo | 4 | 0 | 3 | 0 |
| Health Care, Pharmaceuticals and Biotechnolo | 4 | 0 | 3 | 0 |
| Health Care, Pharmaceuticals and Biotechnolo | 4 | 0 | 3 | 0 |
| Health Care, Pharmaceuticals and Biotechnolo | 4 | 0 | 3 | 0 |
| Health Care, Pharmaceuticals and Biotechnolo | 4 | 0 | 3 | 0 |
| Health Care, Pharmaceuticals and Biotechnolo | 4 | 0 | 3 | 0 |
| Land Development & Construction | 3 | 4 | 2 | 0 |
| Land Development & Construction | 3 | 4 | 2 | 0 |
| Land Development & Construction | 3 | 4 | 2 | 0 |
| Land Development & Construction | 3 | 4 | 2 | 0 |
| Land Development & Construction | 3 | 4 | 2 | 0 |
| Land Development & Construction | 3 | 4 | 2 | 0 |
| Land Development & Construction | 3 | 4 | 2 | 0 |
| Land Development & Construction | 3 | 4 | 2 | 0 |
| Metals & Mining | 5 | 4 | 0 | 0 |
| Metals & Mining | 5 | 4 | 0 | 0 |
| Metals & Mining | 5 | 4 | 0 | 0 |
| Metals & Mining | 5 | 4 | 0 | 0 |
| Metals & Mining | 5 | 4 | 0 | 0 |
| Metals & Mining | 5 | 4 | 0 | 0 |
| Metals & Mining | 5 | 4 | 0 | 0 |
| Offices & Professional Services | 2 | 0 | 0 | 0 |
| Offices & Professional Services | 2 | 0 | 0 | 0 |
| Offices & Professional Services | 2 | 0 | 0 | 0 |
| Offices & Professional Services | 2 | 0 | 0 | 0 |
| Offices & Professional Services | 2 | 0 | 0 | 0 |

| | | | | |
|---|---|---|---|---|
| Offices & Professional Services | 2 | 0 | 0 | 0 |
| Offices & Professional Services | 2 | 0 | 0 | 0 |
| Offices & Professional Services | 2 | 0 | 0 | 0 |
| Offices & Professional Services | 2 | 0 | 0 | 0 |
| Offices & Professional Services | 2 | 0 | 0 | 0 |
| Offices & Professional Services | 2 | 0 | 0 | 0 |
| Offices & Professional Services | 2 | 0 | 0 | 0 |
| Offices & Professional Services | 2 | 0 | 0 | 0 |
| Offices & Professional Services | 2 | 0 | 0 | 0 |
| Offices & Professional Services | 2 | 0 | 0 | 0 |
| Offices & Professional Services | 2 | 0 | 0 | 0 |
| Offices & Professional Services | 2 | 0 | 0 | 0 |
| Offices & Professional Services | 2 | 0 | 0 | 0 |
| Offices & Professional Services | 2 | 0 | 0 | 0 |
| Offices & Professional Services | 2 | 0 | 0 | 0 |
| Offices & Professional Services | 2 | 0 | 0 | 0 |
| Offices & Professional Services | 2 | 0 | 0 | 0 |
| Offices & Professional Services | 2 | 0 | 0 | 0 |
| Offices & Professional Services | 2 | 0 | 0 | 0 |
| Offices & Professional Services | 2 | 0 | 0 | 0 |
| Offices & Professional Services | 2 | 0 | 0 | 0 |
| Offices & Professional Services | 2 | 0 | 0 | 0 |
| Offices & Professional Services | 2 | 0 | 0 | 0 |
| Offices & Professional Services | 2 | 0 | 0 | 0 |
| Offices & Professional Services | 2 | 0 | 0 | 0 |
| Offices & Professional Services | 2 | 0 | 0 | 0 |
| Offices & Professional Services | 2 | 0 | 0 | 0 |
| Offices & Professional Services | 2 | 0 | 0 | 0 |
| Offices & Professional Services | 2 | 0 | 0 | 0 |
| Offices & Professional Services | 2 | 0 | 0 | 0 |
| Offices & Professional Services | 2 | 0 | 0 | 0 |
| Offices & Professional Services | 2 | 0 | 0 | 0 |
| Offices & Professional Services | 2 | 0 | 0 | 0 |
| Offices & Professional Services | 2 | 0 | 0 | 0 |
| Offices & Professional Services | 2 | 0 | 0 | 0 |
| Oil, Gas & Consumable Fuels | 4 | 4 | 0 | 0 |
| Oil, Gas & Consumable Fuels | 4 | 4 | 0 | 0 |
| Oil, Gas & Consumable Fuels | 4 | 4 | 0 | 0 |
| Telecommunication services (including wireles | 2 | 2 | 0 | 0 |

| | | | | |
|---|---|---|---|---|
| Telecommunication services (including wireles | 2 | 2 | 0 | 0 |
| Telecommunication services (including wireles | 2 | 2 | 0 | 0 |
| Telecommunication services (including wireles | 2 | 2 | 0 | 0 |
| Telecommunication services (including wireles | 2 | 2 | 0 | 0 |
| Textiles, Apparel & Luxury Good Production | 5 | 5 | 2 | 0 |
| Transportation Services | 4 | 2 | 0 | 0 |
| Transportation Services | 4 | 2 | 0 | 0 |
| Transportation Services | 4 | 2 | 0 | 0 |
| Transportation Services | 4 | 2 | 0 | 0 |
| Transportation Services | 4 | 2 | 0 | 0 |
| Transportation Services | 4 | 2 | 0 | 0 |

| Soil Condition | Water Condition | Air Condition | Ecosystem Condition | Pollination | Landslides | Wildfire Hazard |
|---|---|---|---|---|---|---|
| 4 | 5 | 3 | 2 | 2 | 5 | 4 |
| 5 | 4 | 3 | 2 | 4 | 5 | 4 |
| 0 | 2 | 2 | 0 | 0 | 4 | 3 |
| 0 | 2 | 2 | 0 | 0 | 4 | 3 |
| 0 | 2 | 2 | 0 | 0 | 4 | 3 |
| 0 | 2 | 2 | 0 | 0 | 4 | 3 |
| 0 | 2 | 2 | 0 | 0 | 4 | 3 |
| 0 | 2 | 2 | 0 | 0 | 4 | 3 |
| 0 | 2 | 2 | 0 | 0 | 4 | 3 |
| 0 | 2 | 2 | 0 | 0 | 4 | 3 |
| 0 | 2 | 2 | 0 | 0 | 4 | 3 |
| 0 | 2 | 2 | 0 | 0 | 4 | 3 |
| 0 | 2 | 2 | 0 | 0 | 4 | 3 |
| 0 | 2 | 2 | 0 | 0 | 4 | 3 |
| 0 | 2 | 2 | 0 | 0 | 4 | 3 |
| 0 | 2 | 2 | 0 | 0 | 4 | 3 |
| 0 | 2 | 2 | 0 | 0 | 4 | 3 |
| 0 | 2 | 2 | 0 | 0 | 4 | 3 |
| 0 | 2 | 2 | 0 | 0 | 4 | 3 |
| 0 | 2 | 2 | 0 | 0 | 4 | 3 |
| 0 | 2 | 2 | 0 | 0 | 4 | 3 |
| 0 | 2 | 2 | 0 | 0 | 4 | 3 |
| 0 | 2 | 2 | 0 | 0 | 4 | 3 |
| 0 | 2 | 2 | 0 | 0 | 4 | 3 |
| 0 | 2 | 2 | 0 | 0 | 4 | 3 |
| 0 | 2 | 2 | 0 | 0 | 4 | 3 |
| 0 | 2 | 2 | 0 | 0 | 4 | 3 |
| 0 | 3 | 2 | 0 | 0 | 4 | 3 |
| 0 | 3 | 2 | 0 | 0 | 4 | 3 |
| 0 | 3 | 2 | 0 | 0 | 4 | 3 |
| 0 | 3 | 2 | 0 | 0 | 4 | 3 |
| 0 | 3 | 2 | 0 | 0 | 4 | 3 |
| 0 | 3 | 2 | 0 | 0 | 4 | 3 |
| 0 | 3 | 2 | 0 | 0 | 4 | 3 |
| 0 | 3 | 2 | 0 | 0 | 4 | 3 |
| 0 | 3 | 2 | 0 | 0 | 4 | 3 |
| 0 | 3 | 2 | 0 | 0 | 4 | 3 |
| 0 | 3 | 2 | 0 | 0 | 4 | 3 |
| 0 | 3 | 2 | 0 | 0 | 4 | 3 |
| 0 | 2 | 2 | 0 | 0 | 4 | 3 |
| 0 | 2 | 2 | 0 | 0 | 4 | 3 |
| 0 | 2 | 2 | 0 | 0 | 4 | 3 |
| 0 | 2 | 2 | 0 | 0 | 4 | 3 |
| 0 | 2 | 2 | 0 | 0 | 4 | 3 |
| 0 | 2 | 2 | 0 | 0 | 4 | 3 |
| 0 | 2 | 2 | 0 | 0 | 4 | 3 |
| 0 | 2 | 2 | 0 | 0 | 4 | 3 |

| | | | | | | |
|---|---|---|---|---|---|---|
| 0 | 2 | 2 | 0 | 0 | 4 | 3 |
| 0 | 2 | 2 | 0 | 0 | 4 | 3 |
| 0 | 2 | 2 | 0 | 0 | 4 | 3 |
| 0 | 2 | 2 | 0 | 0 | 4 | 3 |
| 0 | 2 | 2 | 0 | 0 | 4 | 3 |
| 0 | 2 | 2 | 0 | 0 | 4 | 3 |
| 0 | 2 | 2 | 0 | 0 | 4 | 3 |
| 0 | 2 | 2 | 0 | 0 | 4 | 3 |
| 0 | 2 | 2 | 0 | 0 | 4 | 3 |
| 0 | 2 | 2 | 0 | 0 | 4 | 3 |
| 0 | 3 | 1 | 0 | 0 | 4 | 3 |
| 0 | 2 | 2 | 0 | 0 | 4 | 3 |
| 0 | 2 | 2 | 0 | 0 | 4 | 3 |
| 0 | 2 | 2 | 0 | 0 | 4 | 3 |
| 0 | 2 | 2 | 0 | 0 | 4 | 3 |
| 0 | 2 | 2 | 0 | 0 | 4 | 3 |
| 0 | 2 | 2 | 0 | 0 | 4 | 3 |
| 0 | 2 | 2 | 0 | 0 | 4 | 3 |
| 0 | 2 | 2 | 0 | 0 | 4 | 3 |
| 0 | 2 | 2 | 0 | 0 | 4 | 3 |
| 0 | 2 | 2 | 0 | 0 | 4 | 3 |
| 0 | 2 | 2 | 0 | 0 | 4 | 3 |
| 0 | 2 | 2 | 0 | 0 | 4 | 3 |
| 0 | 2 | 2 | 0 | 0 | 4 | 3 |
| 0 | 2 | 2 | 0 | 0 | 4 | 3 |
| 0 | 2 | 2 | 0 | 0 | 4 | 3 |
| 0 | 2 | 2 | 0 | 0 | 4 | 3 |
| 0 | 2 | 2 | 0 | 0 | 4 | 3 |
| 0 | 2 | 2 | 0 | 0 | 4 | 3 |
| 0 | 2 | 2 | 0 | 0 | 4 | 3 |
| 0 | 2 | 2 | 0 | 0 | 4 | 3 |
| 0 | 2 | 2 | 0 | 0 | 4 | 3 |
| 0 | 4 | 2 | 0 | 0 | 4 | 3 |
| 0 | 4 | 2 | 0 | 0 | 4 | 3 |
| 0 | 4 | 2 | 0 | 0 | 4 | 3 |
| 0 | 4 | 2 | 0 | 0 | 4 | 3 |
| 0 | 4 | 2 | 0 | 0 | 4 | 3 |
| 0 | 4 | 2 | 0 | 0 | 4 | 3 |
| 0 | 4 | 2 | 0 | 0 | 4 | 3 |
| 0 | 4 | 2 | 0 | 0 | 4 | 3 |
| 0 | 4 | 2 | 0 | 0 | 4 | 3 |
| 0 | 4 | 2 | 0 | 0 | 4 | 3 |
| 0 | 4 | 2 | 0 | 0 | 4 | 3 |

| | | | | | | |
|---|---|---|---|---|---|---|
| 0 | 4 | 2 | 0 | 0 | 4 | 3 |
| 0 | 2 | 1 | 0 | 0 | 4 | 3 |
| 0 | 2 | 1 | 0 | 0 | 4 | 3 |
| 0 | 2 | 1 | 0 | 0 | 4 | 3 |
| 0 | 3 | 1 | 0 | 0 | 4 | 3 |
| 0 | 3 | 1 | 0 | 0 | 4 | 3 |
| 0 | 3 | 1 | 0 | 0 | 4 | 3 |
| 0 | 3 | 1 | 0 | 0 | 4 | 3 |
| 0 | 3 | 1 | 0 | 0 | 4 | 3 |
| 0 | 3 | 1 | 0 | 0 | 4 | 3 |
| 0 | 3 | 1 | 0 | 0 | 4 | 3 |
| 0 | 3 | 1 | 0 | 0 | 4 | 3 |
| 0 | 3 | 1 | 0 | 0 | 4 | 3 |
| 0 | 3 | 1 | 0 | 0 | 4 | 3 |
| 0 | 3 | 1 | 0 | 0 | 4 | 3 |
| 0 | 3 | 1 | 0 | 0 | 4 | 3 |
| 0 | 3 | 1 | 0 | 0 | 4 | 3 |
| 0 | 3 | 1 | 0 | 0 | 4 | 3 |
| 0 | 3 | 1 | 0 | 0 | 4 | 3 |
| 0 | 3 | 1 | 0 | 0 | 4 | 3 |
| 1 | 2 | 3 | 0 | 0 | 4 | 3 |
| 1 | 2 | 3 | 0 | 0 | 4 | 3 |
| 1 | 2 | 3 | 0 | 0 | 4 | 3 |
| 1 | 2 | 3 | 0 | 0 | 4 | 3 |
| 1 | 2 | 3 | 0 | 0 | 4 | 3 |
| 1 | 2 | 3 | 0 | 0 | 4 | 3 |
| 1 | 2 | 3 | 0 | 0 | 4 | 3 |
| 1 | 2 | 3 | 0 | 0 | 4 | 3 |
| 0 | 2 | 3 | 0 | 0 | 4 | 3 |
| 0 | 2 | 3 | 0 | 0 | 4 | 3 |
| 0 | 2 | 3 | 0 | 0 | 4 | 3 |
| 0 | 2 | 3 | 0 | 0 | 4 | 3 |
| 0 | 2 | 3 | 0 | 0 | 4 | 3 |
| 0 | 2 | 3 | 0 | 0 | 4 | 3 |
| 0 | 2 | 3 | 0 | 0 | 4 | 3 |
| 0 | 2 | 3 | 0 | 0 | 4 | 3 |
| 0 | 2 | 3 | 0 | 0 | 4 | 3 |
| 0 | 2 | 3 | 0 | 0 | 4 | 3 |
| 0 | 2 | 3 | 0 | 0 | 4 | 3 |
| 0 | 2 | 3 | 0 | 0 | 4 | 3 |

| | | | | | | |
|---|---|---|---|---|---|---|
| 0 | 2 | 3 | 0 | 0 | 4 | 3 |
| 0 | 2 | 3 | 0 | 0 | 4 | 3 |
| 0 | 2 | 3 | 0 | 0 | 4 | 3 |
| 0 | 2 | 3 | 0 | 0 | 4 | 3 |
| 0 | 2 | 3 | 0 | 0 | 4 | 3 |
| 0 | 2 | 3 | 0 | 0 | 4 | 3 |
| 0 | 2 | 3 | 0 | 0 | 4 | 3 |
| 0 | 2 | 3 | 0 | 0 | 4 | 3 |
| 0 | 2 | 3 | 0 | 0 | 4 | 3 |
| 0 | 2 | 3 | 0 | 0 | 4 | 3 |
| 0 | 2 | 3 | 0 | 0 | 4 | 3 |
| 0 | 2 | 3 | 0 | 0 | 4 | 3 |
| 0 | 2 | 3 | 0 | 0 | 4 | 3 |
| 0 | 2 | 3 | 0 | 0 | 4 | 3 |
| 0 | 2 | 3 | 0 | 0 | 4 | 3 |
| 0 | 2 | 3 | 0 | 0 | 4 | 3 |
| 0 | 2 | 3 | 0 | 0 | 4 | 3 |
| 0 | 2 | 3 | 0 | 0 | 4 | 3 |
| 0 | 2 | 3 | 0 | 0 | 4 | 3 |
| 0 | 2 | 3 | 0 | 0 | 4 | 3 |
| 0 | 2 | 3 | 0 | 0 | 4 | 3 |
| 0 | 2 | 3 | 0 | 0 | 4 | 3 |
| 0 | 2 | 3 | 0 | 0 | 4 | 3 |
| 0 | 2 | 3 | 0 | 0 | 4 | 3 |
| 0 | 2 | 3 | 0 | 0 | 4 | 3 |
| 0 | 2 | 3 | 0 | 0 | 4 | 3 |
| 0 | 2 | 3 | 0 | 0 | 4 | 3 |
| 0 | 2 | 3 | 0 | 0 | 4 | 3 |
| 0 | 2 | 3 | 0 | 0 | 4 | 3 |
| 0 | 2 | 3 | 0 | 0 | 4 | 3 |
| 0 | 2 | 3 | 0 | 0 | 4 | 3 |
| 0 | 2 | 3 | 0 | 0 | 4 | 3 |
| 0 | 2 | 3 | 0 | 0 | 4 | 3 |
| 0 | 2 | 3 | 0 | 0 | 4 | 3 |
| 0 | 2 | 3 | 0 | 0 | 4 | 3 |
| 0 | 2 | 3 | 0 | 0 | 4 | 3 |
| 0 | 2 | 3 | 0 | 0 | 4 | 3 |
| 0 | 2 | 3 | 0 | 0 | 4 | 3 |
| 0 | 2 | 3 | 0 | 0 | 4 | 3 |
| 0 | 2 | 0 | 0 | 0 | 4 | 3 |

| | | | | | | |
|---|---|---|---|---|---|---|
| 0 | 2 | 0 | 0 | 0 | 4 | 3 |
| 0 | 2 | 0 | 0 | 0 | 4 | 3 |
| 0 | 2 | 0 | 0 | 0 | 4 | 3 |
| 0 | 2 | 0 | 0 | 0 | 4 | 3 |
| 0 | 2 | 2 | 0 | 0 | 4 | 3 |
| 0 | 2 | 2 | 0 | 0 | 5 | 4 |
| 0 | 2 | 2 | 0 | 0 | 5 | 4 |
| 0 | 2 | 2 | 0 | 0 | 5 | 4 |
| 0 | 2 | 2 | 0 | 0 | 5 | 4 |
| 0 | 2 | 2 | 0 | 0 | 5 | 4 |
| 0 | 2 | 2 | 0 | 0 | 5 | 4 |

| Plant/Forest/ Aquatic Pests | Herbicide Resistance | Extreme Heat | Tropical Cyclones | Tourism Attractiveness | Media Scrutiny | Political Situation |
|---|---|---|---|---|---|---|
| 4 | 4 | 5 | 5 | 0 | 5 | 3 |
| 4 | 4 | 5 | 5 | 0 | 5 | 3 |
| 0 | 0 | 3 | 4 | 0 | 3 | 2 |
| 0 | 0 | 3 | 4 | 0 | 3 | 2 |
| 0 | 0 | 3 | 4 | 0 | 3 | 2 |
| 0 | 0 | 3 | 4 | 0 | 1 | 2 |
| 0 | 0 | 3 | 4 | 0 | 1 | 2 |
| 0 | 0 | 3 | 4 | 0 | 1 | 2 |
| 0 | 0 | 3 | 4 | 0 | 1 | 2 |
| 0 | 0 | 3 | 4 | 0 | 1 | 2 |
| 0 | 0 | 3 | 4 | 0 | 1 | 2 |
| 0 | 0 | 3 | 4 | 0 | 1 | 2 |
| 0 | 0 | 3 | 4 | 0 | 1 | 2 |
| 0 | 0 | 3 | 4 | 0 | 1 | 2 |
| 0 | 0 | 3 | 4 | 0 | 1 | 2 |
| 0 | 0 | 3 | 4 | 0 | 1 | 2 |
| 0 | 0 | 3 | 4 | 0 | 1 | 2 |
| 0 | 0 | 3 | 4 | 0 | 1 | 2 |
| 0 | 0 | 3 | 4 | 0 | 1 | 2 |
| 0 | 0 | 3 | 4 | 0 | 1 | 2 |
| 0 | 0 | 3 | 4 | 0 | 1 | 2 |
| 0 | 0 | 3 | 4 | 0 | 1 | 2 |
| 0 | 0 | 3 | 4 | 0 | 1 | 2 |
| 0 | 0 | 3 | 4 | 0 | 1 | 2 |
| 0 | 0 | 3 | 4 | 0 | 1 | 2 |
| 0 | 0 | 3 | 4 | 0 | 1 | 2 |
| 0 | 0 | 3 | 4 | 0 | 1 | 2 |
| 0 | 0 | 3 | 4 | 0 | 4 | 2 |
| 0 | 0 | 3 | 4 | 0 | 4 | 2 |
| 0 | 0 | 3 | 4 | 0 | 4 | 2 |
| 0 | 0 | 3 | 4 | 0 | 4 | 2 |
| 0 | 0 | 3 | 4 | 0 | 4 | 2 |
| 0 | 0 | 3 | 4 | 0 | 4 | 2 |
| 0 | 0 | 3 | 4 | 0 | 4 | 2 |
| 0 | 0 | 3 | 4 | 0 | 4 | 2 |
| 0 | 0 | 3 | 4 | 0 | 4 | 2 |
| 0 | 0 | 3 | 4 | 0 | 4 | 2 |
| 0 | 0 | 3 | 4 | 0 | 4 | 2 |
| 0 | 0 | 3 | 4 | 0 | 4 | 2 |
| 0 | 0 | 3 | 4 | 0 | 4 | 2 |
| 0 | 0 | 3 | 4 | 0 | 4 | 2 |
| 0 | 0 | 3 | 4 | 0 | 4 | 2 |
| 0 | 0 | 3 | 4 | 0 | 4 | 2 |
| 0 | 0 | 4 | 4 | 0 | 2 | 3 |
| 0 | 0 | 4 | 4 | 0 | 2 | 3 |
| 0 | 0 | 4 | 4 | 0 | 2 | 3 |
| 0 | 0 | 4 | 4 | 0 | 2 | 3 |

| | | | | | | |
|---|---|---|---|---|---|---|
| 0 | 0 | 4 | 4 | 0 | 2 | 3 |
| 0 | 0 | 4 | 4 | 0 | 2 | 3 |
| 0 | 0 | 4 | 4 | 0 | 2 | 3 |
| 0 | 0 | 3 | 4 | 0 | 2 | 2 |
| 0 | 0 | 3 | 4 | 0 | 2 | 2 |
| 0 | 0 | 3 | 4 | 0 | 2 | 2 |
| 0 | 0 | 3 | 4 | 0 | 2 | 2 |
| 0 | 0 | 3 | 4 | 0 | 2 | 2 |
| 0 | 0 | 3 | 4 | 0 | 2 | 2 |
| 0 | 0 | 3 | 4 | 0 | 2 | 2 |
| 0 | 0 | 3 | 4 | 0 | 2 | 3 |
| 0 | 0 | 3 | 4 | 0 | 1 | 2 |
| 0 | 0 | 3 | 4 | 0 | 1 | 2 |
| 0 | 0 | 3 | 4 | 0 | 1 | 2 |
| 0 | 0 | 3 | 4 | 0 | 1 | 2 |
| 0 | 0 | 3 | 4 | 0 | 1 | 2 |
| 0 | 0 | 3 | 4 | 0 | 1 | 2 |
| 0 | 0 | 3 | 4 | 0 | 1 | 2 |
| 0 | 0 | 3 | 4 | 0 | 1 | 2 |
| 0 | 0 | 3 | 4 | 0 | 1 | 2 |
| 0 | 0 | 3 | 4 | 0 | 1 | 2 |
| 0 | 0 | 3 | 4 | 0 | 1 | 2 |
| 0 | 0 | 3 | 4 | 0 | 1 | 2 |
| 0 | 0 | 3 | 4 | 0 | 1 | 2 |
| 0 | 0 | 3 | 4 | 0 | 1 | 2 |
| 0 | 0 | 3 | 4 | 0 | 1 | 2 |
| 0 | 0 | 3 | 4 | 0 | 1 | 2 |
| 0 | 0 | 3 | 4 | 0 | 1 | 2 |
| 0 | 0 | 3 | 4 | 0 | 1 | 2 |
| 0 | 0 | 3 | 4 | 0 | 1 | 2 |
| 0 | 0 | 3 | 4 | 0 | 1 | 2 |
| 4 | 0 | 3 | 4 | 0 | 5 | 3 |
| 4 | 0 | 3 | 4 | 0 | 5 | 3 |
| 4 | 0 | 3 | 4 | 0 | 5 | 3 |
| 4 | 0 | 3 | 4 | 0 | 5 | 3 |
| 4 | 0 | 3 | 4 | 0 | 5 | 3 |
| 4 | 0 | 3 | 4 | 0 | 5 | 3 |
| 4 | 0 | 3 | 4 | 0 | 5 | 3 |
| 4 | 0 | 3 | 4 | 0 | 5 | 3 |
| 4 | 0 | 3 | 4 | 0 | 5 | 3 |
| 4 | 0 | 3 | 4 | 0 | 5 | 3 |
| 4 | 0 | 3 | 4 | 0 | 5 | 3 |

| | | | | | | |
|---|---|---|---|---|---|---|
| 4 | 0 | 3 | 4 | 0 | 5 | 3 |
| 0 | 0 | 3 | 4 | 0 | 3 | 1 |
| 0 | 0 | 3 | 4 | 0 | 3 | 1 |
| 0 | 0 | 3 | 4 | 0 | 3 | 1 |
| 4 | 0 | 3 | 4 | 0 | 1 | 2 |
| 4 | 0 | 3 | 4 | 0 | 1 | 2 |
| 4 | 0 | 3 | 4 | 0 | 1 | 2 |
| 4 | 0 | 3 | 4 | 0 | 1 | 2 |
| 4 | 0 | 3 | 4 | 0 | 1 | 2 |
| 4 | 0 | 3 | 4 | 0 | 1 | 2 |
| 4 | 0 | 3 | 4 | 0 | 1 | 2 |
| 4 | 0 | 3 | 4 | 0 | 1 | 2 |
| 4 | 0 | 3 | 4 | 0 | 1 | 2 |
| 4 | 0 | 3 | 4 | 0 | 1 | 2 |
| 4 | 0 | 3 | 4 | 0 | 1 | 2 |
| 4 | 0 | 3 | 4 | 0 | 1 | 2 |
| 4 | 0 | 3 | 4 | 0 | 1 | 2 |
| 4 | 0 | 3 | 4 | 0 | 1 | 2 |
| 4 | 0 | 3 | 4 | 0 | 1 | 2 |
| 4 | 0 | 3 | 4 | 0 | 1 | 2 |
| 0 | 1 | 4 | 4 | 1 | 4 | 3 |
| 0 | 1 | 4 | 4 | 1 | 4 | 3 |
| 0 | 1 | 4 | 4 | 1 | 4 | 3 |
| 0 | 1 | 4 | 4 | 1 | 4 | 3 |
| 0 | 1 | 4 | 4 | 1 | 4 | 3 |
| 0 | 1 | 4 | 4 | 1 | 4 | 3 |
| 0 | 1 | 4 | 4 | 1 | 4 | 3 |
| 0 | 1 | 4 | 4 | 1 | 4 | 3 |
| 0 | 0 | 4 | 4 | 0 | 5 | 3 |
| 0 | 0 | 4 | 4 | 0 | 5 | 3 |
| 0 | 0 | 4 | 4 | 0 | 5 | 3 |
| 0 | 0 | 4 | 4 | 0 | 5 | 3 |
| 0 | 0 | 4 | 4 | 0 | 5 | 3 |
| 0 | 0 | 4 | 4 | 0 | 5 | 3 |
| 0 | 0 | 4 | 4 | 0 | 5 | 3 |
| 0 | 0 | 4 | 4 | 0 | 3 | 1 |
| 0 | 0 | 4 | 4 | 0 | 3 | 1 |
| 0 | 0 | 4 | 4 | 0 | 3 | 1 |
| 0 | 0 | 4 | 4 | 0 | 3 | 1 |
| 0 | 0 | 4 | 4 | 0 | 3 | 1 |

| | | | | | | |
|---|---|---|---|---|---|---|
| 0 | 0 | 4 | 4 | 0 | 3 | 1 |
| 0 | 0 | 4 | 4 | 0 | 3 | 1 |
| 0 | 0 | 4 | 4 | 0 | 3 | 1 |
| 0 | 0 | 4 | 4 | 0 | 3 | 1 |
| 0 | 0 | 4 | 4 | 0 | 3 | 1 |
| 0 | 0 | 4 | 4 | 0 | 3 | 1 |
| 0 | 0 | 4 | 4 | 0 | 3 | 1 |
| 0 | 0 | 4 | 4 | 0 | 3 | 1 |
| 0 | 0 | 4 | 4 | 0 | 3 | 1 |
| 0 | 0 | 4 | 4 | 0 | 3 | 1 |
| 0 | 0 | 4 | 4 | 0 | 3 | 1 |
| 0 | 0 | 4 | 4 | 0 | 3 | 1 |
| 0 | 0 | 4 | 4 | 0 | 3 | 1 |
| 0 | 0 | 4 | 4 | 0 | 3 | 1 |
| 0 | 0 | 4 | 4 | 0 | 3 | 1 |
| 0 | 0 | 4 | 4 | 0 | 3 | 1 |
| 0 | 0 | 4 | 4 | 0 | 3 | 1 |
| 0 | 0 | 4 | 4 | 0 | 3 | 1 |
| 0 | 0 | 4 | 4 | 0 | 3 | 1 |
| 0 | 0 | 4 | 4 | 0 | 3 | 1 |
| 0 | 0 | 4 | 4 | 0 | 3 | 1 |
| 0 | 0 | 4 | 4 | 0 | 3 | 1 |
| 0 | 0 | 4 | 4 | 0 | 3 | 1 |
| 0 | 0 | 4 | 4 | 0 | 3 | 1 |
| 0 | 0 | 4 | 4 | 0 | 3 | 1 |
| 0 | 0 | 4 | 4 | 0 | 3 | 1 |
| 0 | 0 | 4 | 4 | 0 | 3 | 1 |
| 0 | 0 | 4 | 4 | 0 | 3 | 1 |
| 0 | 0 | 4 | 4 | 0 | 3 | 1 |
| 0 | 0 | 4 | 4 | 0 | 3 | 1 |
| 0 | 0 | 4 | 4 | 0 | 3 | 1 |
| 0 | 0 | 4 | 4 | 0 | 3 | 1 |
| 0 | 0 | 4 | 4 | 0 | 3 | 1 |
| 0 | 0 | 4 | 4 | 0 | 3 | 1 |
| 0 | 0 | 4 | 4 | 0 | 3 | 1 |
| 0 | 0 | 4 | 4 | 0 | 3 | 1 |
| 0 | 0 | 4 | 4 | 0 | 4 | 3 |
| 0 | 0 | 4 | 4 | 0 | 4 | 3 |
| 0 | 0 | 4 | 4 | 0 | 4 | 3 |
| 0 | 0 | 3 | 4 | 0 | 1 | 2 |

| | | | | | | |
|---|---|---|---|---|---|---|
| 0 | 0 | 3 | 4 | 0 | 1 | 2 |
| 0 | 0 | 3 | 4 | 0 | 1 | 2 |
| 0 | 0 | 3 | 4 | 0 | 1 | 2 |
| 0 | 0 | 3 | 4 | 0 | 1 | 2 |
| 0 | 0 | 3 | 4 | 0 | 3 | 2 |
| 0 | 0 | 4 | 5 | 0 | 1 | 3 |
| 0 | 0 | 4 | 5 | 0 | 1 | 3 |
| 0 | 0 | 4 | 5 | 0 | 1 | 3 |
| 0 | 0 | 4 | 5 | 0 | 1 | 3 |
| 0 | 0 | 4 | 5 | 0 | 1 | 3 |
| 0 | 0 | 4 | 5 | 0 | 1 | 3 |

| Sites of International | Risk Preparation | Land, Freshwater | Tree Cover Loss | Invasives | Pollution | Protected/Conserved Areas |
|---|---|---|---|---|---|---|
| 3 | 2 | 5 | 5 | 3 | 5 | 5 |
| 3 | 2 | 5 | 5 | 3 | 5 | 5 |
| 2 | 2 | 1 | 1 | 0 | 5 | 3 |
| 2 | 2 | 1 | 1 | 0 | 5 | 3 |
| 2 | 2 | 1 | 1 | 0 | 5 | 3 |
| 2 | 2 | 1 | 1 | 0 | 5 | 3 |
| 2 | 2 | 1 | 1 | 0 | 5 | 3 |
| 2 | 2 | 1 | 1 | 0 | 5 | 3 |
| 2 | 2 | 1 | 1 | 0 | 5 | 3 |
| 2 | 2 | 1 | 1 | 0 | 5 | 3 |
| 2 | 2 | 1 | 1 | 0 | 5 | 3 |
| 2 | 2 | 1 | 1 | 0 | 5 | 3 |
| 2 | 2 | 1 | 1 | 0 | 5 | 3 |
| 2 | 2 | 1 | 1 | 0 | 5 | 3 |
| 2 | 2 | 1 | 1 | 0 | 5 | 3 |
| 2 | 2 | 1 | 1 | 0 | 5 | 3 |
| 2 | 2 | 1 | 1 | 0 | 5 | 3 |
| 2 | 2 | 1 | 1 | 0 | 5 | 3 |
| 2 | 2 | 1 | 1 | 0 | 5 | 3 |
| 2 | 2 | 1 | 1 | 0 | 5 | 3 |
| 2 | 2 | 1 | 1 | 0 | 5 | 3 |
| 2 | 2 | 1 | 1 | 0 | 5 | 3 |
| 2 | 2 | 1 | 1 | 0 | 5 | 3 |
| 2 | 2 | 1 | 1 | 0 | 5 | 3 |
| 2 | 2 | 1 | 1 | 0 | 5 | 3 |
| 2 | 2 | 1 | 1 | 0 | 5 | 3 |
| 2 | 2 | 1 | 1 | 0 | 5 | 3 |
| 2 | 2 | 1 | 1 | 0 | 5 | 3 |
| 2 | 2 | 1 | 1 | 0 | 5 | 3 |
| 2 | 2 | 1 | 1 | 0 | 5 | 3 |
| 2 | 2 | 1 | 1 | 0 | 5 | 3 |
| 2 | 2 | 1 | 1 | 0 | 5 | 3 |
| 2 | 2 | 1 | 1 | 0 | 5 | 3 |
| 2 | 2 | 1 | 1 | 0 | 5 | 3 |
| 2 | 2 | 1 | 1 | 0 | 5 | 3 |
| 2 | 2 | 1 | 1 | 0 | 5 | 3 |
| 2 | 2 | 1 | 1 | 0 | 5 | 3 |
| 2 | 2 | 1 | 1 | 0 | 5 | 3 |
| 2 | 2 | 1 | 1 | 0 | 5 | 3 |
| 2 | 2 | 1 | 5 | 2 | 5 | 3 |
| 2 | 2 | 1 | 5 | 2 | 5 | 3 |
| 2 | 2 | 1 | 5 | 2 | 5 | 3 |
| 2 | 2 | 1 | 5 | 2 | 5 | 3 |
| 3 | 2 | 1 | 4 | 0 | 5 | 5 |
| 3 | 2 | 1 | 4 | 0 | 5 | 5 |
| 3 | 2 | 1 | 4 | 0 | 5 | 5 |
| 3 | 2 | 1 | 4 | 0 | 5 | 5 |

| | | | | | | |
|---|---|---|---|---|---|---|
| 3 | 2 | 1 | 4 | 0 | 5 | 5 |
| 3 | 2 | 1 | 4 | 0 | 5 | 5 |
| 3 | 2 | 1 | 4 | 0 | 5 | 5 |
| 2 | 2 | 3 | 1 | 0 | 4 | 4 |
| 2 | 2 | 3 | 1 | 0 | 4 | 4 |
| 2 | 2 | 3 | 1 | 0 | 4 | 4 |
| 2 | 2 | 3 | 1 | 0 | 4 | 4 |
| 2 | 2 | 3 | 1 | 0 | 4 | 4 |
| 2 | 2 | 3 | 1 | 0 | 4 | 4 |
| 2 | 2 | 3 | 1 | 0 | 4 | 4 |
| 3 | 2 | 5 | 4 | 2 | 4 | 4 |
| 2 | 2 | 1 | 1 | 0 | 5 | 3 |
| 2 | 2 | 1 | 1 | 0 | 5 | 3 |
| 2 | 2 | 1 | 1 | 0 | 5 | 3 |
| 2 | 2 | 1 | 1 | 0 | 5 | 3 |
| 2 | 2 | 1 | 1 | 0 | 5 | 3 |
| 2 | 2 | 1 | 1 | 0 | 5 | 3 |
| 2 | 2 | 1 | 1 | 0 | 5 | 3 |
| 2 | 2 | 1 | 1 | 0 | 5 | 3 |
| 2 | 2 | 1 | 1 | 0 | 5 | 3 |
| 2 | 2 | 1 | 1 | 0 | 5 | 3 |
| 2 | 2 | 1 | 1 | 0 | 5 | 3 |
| 2 | 2 | 1 | 1 | 0 | 5 | 3 |
| 2 | 2 | 1 | 1 | 0 | 5 | 3 |
| 2 | 2 | 1 | 1 | 0 | 5 | 3 |
| 2 | 2 | 1 | 1 | 0 | 5 | 3 |
| 2 | 2 | 1 | 1 | 0 | 5 | 3 |
| 2 | 2 | 1 | 1 | 0 | 5 | 3 |
| 2 | 2 | 1 | 1 | 0 | 5 | 3 |
| 2 | 2 | 1 | 1 | 0 | 5 | 3 |
| 2 | 2 | 1 | 1 | 0 | 5 | 3 |
| 2 | 2 | 1 | 1 | 2 | 4 | 3 |
| 2 | 2 | 1 | 1 | 2 | 4 | 3 |
| 2 | 2 | 1 | 1 | 2 | 4 | 3 |
| 2 | 2 | 1 | 1 | 2 | 4 | 3 |
| 2 | 2 | 1 | 1 | 2 | 4 | 3 |
| 2 | 2 | 1 | 1 | 2 | 4 | 3 |
| 2 | 2 | 1 | 1 | 2 | 4 | 3 |
| 2 | 2 | 1 | 1 | 2 | 4 | 3 |
| 2 | 2 | 1 | 1 | 2 | 4 | 3 |
| 2 | 2 | 1 | 1 | 2 | 4 | 3 |
| 2 | 2 | 1 | 1 | 2 | 4 | 3 |

| | | | | | | |
|---|---|---|---|---|---|---|
| 2 | 2 | 1 | 1 | 2 | 4 | 3 |
| 0 | 2 | 1 | 1 | 2 | 4 | 1 |
| 0 | 2 | 1 | 1 | 2 | 4 | 1 |
| 0 | 2 | 1 | 1 | 2 | 4 | 1 |
| 2 | 2 | 1 | 1 | 0 | 5 | 3 |
| 2 | 2 | 1 | 1 | 0 | 5 | 3 |
| 2 | 2 | 1 | 1 | 0 | 5 | 3 |
| 2 | 2 | 1 | 1 | 0 | 5 | 3 |
| 2 | 2 | 1 | 1 | 0 | 5 | 3 |
| 2 | 2 | 1 | 1 | 0 | 5 | 3 |
| 2 | 2 | 1 | 1 | 0 | 5 | 3 |
| 2 | 2 | 1 | 1 | 0 | 5 | 3 |
| 2 | 2 | 1 | 1 | 0 | 5 | 3 |
| 2 | 2 | 1 | 1 | 0 | 5 | 3 |
| 2 | 2 | 1 | 1 | 0 | 5 | 3 |
| 2 | 2 | 1 | 1 | 0 | 5 | 3 |
| 2 | 2 | 1 | 1 | 0 | 5 | 3 |
| 2 | 2 | 1 | 1 | 0 | 5 | 3 |
| 2 | 2 | 1 | 1 | 0 | 5 | 3 |
| 2 | 2 | 1 | 1 | 0 | 5 | 3 |
| 3 | 2 | 5 | 5 | 2 | 5 | 5 |
| 3 | 2 | 5 | 5 | 2 | 5 | 5 |
| 3 | 2 | 5 | 5 | 2 | 5 | 5 |
| 3 | 2 | 5 | 5 | 2 | 5 | 5 |
| 3 | 2 | 5 | 5 | 2 | 5 | 5 |
| 3 | 2 | 5 | 5 | 2 | 5 | 5 |
| 3 | 2 | 5 | 5 | 2 | 5 | 5 |
| 3 | 2 | 5 | 5 | 2 | 5 | 5 |
| 3 | 2 | 5 | 5 | 2 | 5 | 5 |
| 3 | 2 | 5 | 5 | 2 | 5 | 5 |
| 3 | 2 | 5 | 5 | 2 | 5 | 5 |
| 3 | 2 | 5 | 5 | 2 | 5 | 5 |
| 3 | 2 | 5 | 5 | 2 | 5 | 5 |
| 3 | 2 | 5 | 5 | 2 | 5 | 5 |
| 3 | 2 | 5 | 5 | 2 | 5 | 5 |
| 0 | 2 | 1 | 1 | 0 | 2 | 1 |
| 0 | 2 | 1 | 1 | 0 | 2 | 1 |
| 0 | 2 | 1 | 1 | 0 | 2 | 1 |
| 0 | 2 | 1 | 1 | 0 | 2 | 1 |
| 0 | 2 | 1 | 1 | 0 | 2 | 1 |

| | | | | | | |
|---|---|---|---|---|---|---|
| 0 | 2 | 1 | 1 | 0 | 2 | 1 |
| 0 | 2 | 1 | 1 | 0 | 2 | 1 |
| 0 | 2 | 1 | 1 | 0 | 2 | 1 |
| 0 | 2 | 1 | 1 | 0 | 2 | 1 |
| 0 | 2 | 1 | 1 | 0 | 2 | 1 |
| 0 | 2 | 1 | 1 | 0 | 2 | 1 |
| 0 | 2 | 1 | 1 | 0 | 2 | 1 |
| 0 | 2 | 1 | 1 | 0 | 2 | 1 |
| 0 | 2 | 1 | 1 | 0 | 2 | 1 |
| 0 | 2 | 1 | 1 | 0 | 2 | 1 |
| 0 | 2 | 1 | 1 | 0 | 2 | 1 |
| 0 | 2 | 1 | 1 | 0 | 2 | 1 |
| 0 | 2 | 1 | 1 | 0 | 2 | 1 |
| 0 | 2 | 1 | 1 | 0 | 2 | 1 |
| 0 | 2 | 1 | 1 | 0 | 2 | 1 |
| 0 | 2 | 1 | 1 | 0 | 2 | 1 |
| 0 | 2 | 1 | 1 | 0 | 2 | 1 |
| 0 | 2 | 1 | 1 | 0 | 2 | 1 |
| 0 | 2 | 1 | 1 | 0 | 2 | 1 |
| 0 | 2 | 1 | 1 | 0 | 2 | 1 |
| 0 | 2 | 1 | 1 | 0 | 2 | 1 |
| 0 | 2 | 1 | 1 | 0 | 2 | 1 |
| 0 | 2 | 1 | 1 | 0 | 2 | 1 |
| 0 | 2 | 1 | 1 | 0 | 2 | 1 |
| 0 | 2 | 1 | 1 | 0 | 2 | 1 |
| 0 | 2 | 1 | 1 | 0 | 2 | 1 |
| 0 | 2 | 1 | 1 | 0 | 2 | 1 |
| 0 | 2 | 1 | 1 | 0 | 2 | 1 |
| 0 | 2 | 1 | 1 | 0 | 2 | 1 |
| 0 | 2 | 1 | 1 | 0 | 2 | 1 |
| 0 | 2 | 1 | 1 | 0 | 2 | 1 |
| 0 | 2 | 1 | 1 | 0 | 2 | 1 |
| 0 | 2 | 1 | 1 | 0 | 2 | 1 |
| 0 | 2 | 1 | 1 | 0 | 2 | 1 |
| 0 | 2 | 1 | 1 | 0 | 2 | 1 |
| 0 | 2 | 1 | 1 | 0 | 2 | 1 |
| 3 | 2 | 5 | 5 | 2 | 5 | 5 |
| 3 | 2 | 5 | 5 | 2 | 5 | 5 |
| 3 | 2 | 5 | 5 | 2 | 5 | 5 |
| 2 | 2 | 3 | 5 | 2 | 2 | 3 |

| | | | | | | |
|---|---|---|---|---|---|---|
| 2 | 2 | 3 | 5 | 2 | 2 | 3 |
| 2 | 2 | 3 | 5 | 2 | 2 | 3 |
| 2 | 2 | 3 | 5 | 2 | 2 | 3 |
| 2 | 2 | 3 | 5 | 2 | 2 | 3 |
| 2 | 2 | 1 | 1 | 0 | 5 | 3 |
| 3 | 2 | 5 | 5 | 3 | 4 | 5 |
| 3 | 2 | 5 | 5 | 3 | 4 | 5 |
| 3 | 2 | 5 | 5 | 3 | 4 | 5 |
| 3 | 2 | 5 | 5 | 3 | 4 | 5 |
| 3 | 2 | 5 | 5 | 3 | 4 | 5 |
| 3 | 2 | 5 | 5 | 3 | 4 | 5 |

| Key Biodiversity | Other Important | Ecosystem Condition | Range Rarity | Indigenous Peoples (IPs): | Resource Scarcity: Food | Labor/Human Rights |
|---|---|---|---|---|---|---|
| 4 | 4 | 4 | 3 | 5 | 3 | 4 |
| 4 | 4 | 4 | 3 | 5 | 3 | 4 |
| 2 | 2 | 2 | 1 | 3 | 1 | 2 |
| 2 | 2 | 2 | 1 | 3 | 1 | 2 |
| 2 | 2 | 2 | 1 | 3 | 1 | 2 |
| 2 | 2 | 2 | 1 | 3 | 1 | 2 |
| 2 | 2 | 2 | 1 | 3 | 1 | 2 |
| 2 | 2 | 2 | 1 | 3 | 1 | 2 |
| 2 | 2 | 2 | 1 | 3 | 1 | 2 |
| 2 | 2 | 2 | 1 | 3 | 1 | 2 |
| 2 | 2 | 2 | 1 | 3 | 1 | 2 |
| 2 | 2 | 2 | 1 | 3 | 1 | 2 |
| 2 | 2 | 2 | 1 | 3 | 1 | 2 |
| 2 | 2 | 2 | 1 | 3 | 1 | 2 |
| 2 | 2 | 2 | 1 | 3 | 1 | 2 |
| 2 | 2 | 2 | 1 | 3 | 1 | 2 |
| 2 | 2 | 2 | 1 | 3 | 1 | 2 |
| 2 | 2 | 2 | 1 | 3 | 1 | 2 |
| 2 | 2 | 2 | 1 | 3 | 1 | 2 |
| 2 | 2 | 2 | 1 | 3 | 1 | 2 |
| 2 | 2 | 2 | 1 | 3 | 1 | 2 |
| 2 | 2 | 2 | 1 | 3 | 1 | 2 |
| 2 | 2 | 2 | 1 | 3 | 1 | 2 |
| 2 | 2 | 2 | 1 | 3 | 1 | 2 |
| 2 | 2 | 2 | 1 | 3 | 1 | 2 |
| 2 | 2 | 2 | 1 | 3 | 1 | 2 |
| 2 | 2 | 2 | 1 | 3 | 1 | 2 |
| 2 | 2 | 2 | 1 | 3 | 1 | 2 |
| 2 | 2 | 2 | 1 | 3 | 1 | 2 |
| 2 | 2 | 2 | 1 | 3 | 1 | 2 |
| 2 | 2 | 2 | 1 | 3 | 1 | 2 |
| 2 | 2 | 2 | 1 | 3 | 1 | 2 |
| 2 | 2 | 2 | 1 | 3 | 1 | 2 |
| 2 | 2 | 2 | 1 | 3 | 1 | 2 |
| 2 | 2 | 2 | 1 | 3 | 1 | 2 |
| 2 | 2 | 2 | 1 | 3 | 1 | 2 |
| 2 | 2 | 2 | 1 | 3 | 1 | 2 |
| 2 | 2 | 2 | 1 | 3 | 1 | 2 |
| 2 | 2 | 2 | 1 | 3 | 1 | 2 |
| 2 | 2 | 2 | 1 | 3 | 1 | 2 |
| 2 | 2 | 2 | 1 | 3 | 1 | 2 |
| 2 | 2 | 2 | 1 | 3 | 1 | 2 |
| 2 | 2 | 2 | 1 | 3 | 1 | 2 |
| 4 | 4 | 4 | 3 | 3 | 1 | 2 |
| 4 | 4 | 4 | 3 | 3 | 1 | 2 |
| 4 | 4 | 4 | 3 | 3 | 1 | 2 |
| 4 | 4 | 4 | 3 | 3 | 1 | 2 |

| | | | | | | |
|---|---|---|---|---|---|---|
| 4 | 4 | 4 | 3 | 3 | 1 | 2 |
| 4 | 4 | 4 | 3 | 3 | 1 | 2 |
| 4 | 4 | 4 | 3 | 3 | 1 | 2 |
| 3 | 2 | 2 | 3 | 3 | 0 | 2 |
| 3 | 2 | 2 | 3 | 3 | 0 | 2 |
| 3 | 2 | 2 | 3 | 3 | 0 | 2 |
| 3 | 2 | 2 | 3 | 3 | 0 | 2 |
| 3 | 2 | 2 | 3 | 3 | 0 | 2 |
| 3 | 2 | 2 | 3 | 3 | 0 | 2 |
| 3 | 2 | 2 | 3 | 3 | 0 | 2 |
| 3 | 4 | 4 | 3 | 5 | 1 | 2 |
| 2 | 2 | 2 | 1 | 3 | 1 | 2 |
| 2 | 2 | 2 | 1 | 3 | 1 | 2 |
| 2 | 2 | 2 | 1 | 3 | 1 | 2 |
| 2 | 2 | 2 | 1 | 3 | 1 | 2 |
| 2 | 2 | 2 | 1 | 3 | 1 | 2 |
| 2 | 2 | 2 | 1 | 3 | 1 | 2 |
| 2 | 2 | 2 | 1 | 3 | 1 | 2 |
| 2 | 2 | 2 | 1 | 3 | 1 | 2 |
| 2 | 2 | 2 | 1 | 3 | 1 | 2 |
| 2 | 2 | 2 | 1 | 3 | 1 | 2 |
| 2 | 2 | 2 | 1 | 3 | 1 | 2 |
| 2 | 2 | 2 | 1 | 3 | 1 | 2 |
| 2 | 2 | 2 | 1 | 3 | 1 | 2 |
| 2 | 2 | 2 | 1 | 3 | 1 | 2 |
| 2 | 2 | 2 | 1 | 3 | 1 | 2 |
| 2 | 2 | 2 | 1 | 3 | 1 | 2 |
| 2 | 2 | 2 | 1 | 3 | 1 | 2 |
| 2 | 2 | 2 | 1 | 3 | 1 | 2 |
| 2 | 2 | 2 | 1 | 3 | 1 | 2 |
| 2 | 2 | 2 | 1 | 3 | 1 | 2 |
| 2 | 2 | 2 | 1 | 3 | 2 | 2 |
| 2 | 2 | 2 | 1 | 3 | 2 | 2 |
| 2 | 2 | 2 | 1 | 3 | 2 | 2 |
| 2 | 2 | 2 | 1 | 3 | 2 | 2 |
| 2 | 2 | 2 | 1 | 3 | 2 | 2 |
| 2 | 2 | 2 | 1 | 3 | 2 | 2 |
| 2 | 2 | 2 | 1 | 3 | 2 | 2 |
| 2 | 2 | 2 | 1 | 3 | 2 | 2 |
| 2 | 2 | 2 | 1 | 3 | 2 | 2 |
| 2 | 2 | 2 | 1 | 3 | 2 | 2 |
| 2 | 2 | 2 | 1 | 3 | 2 | 2 |

| | | | | | | |
|---|---|---|---|---|---|---|
| 2 | 2 | 2 | 1 | 3 | 2 | 2 |
| 1 | 1 | 1 | 0 | 1 | 0 | 2 |
| 1 | 1 | 1 | 0 | 1 | 0 | 2 |
| 1 | 1 | 1 | 0 | 1 | 0 | 2 |
| 2 | 2 | 2 | 1 | 3 | 1 | 2 |
| 2 | 2 | 2 | 1 | 3 | 1 | 2 |
| 2 | 2 | 2 | 1 | 3 | 1 | 2 |
| 2 | 2 | 2 | 1 | 3 | 1 | 2 |
| 2 | 2 | 2 | 1 | 3 | 1 | 2 |
| 2 | 2 | 2 | 1 | 3 | 1 | 2 |
| 2 | 2 | 2 | 1 | 3 | 1 | 2 |
| 2 | 2 | 2 | 1 | 3 | 1 | 2 |
| 2 | 2 | 2 | 1 | 3 | 1 | 2 |
| 2 | 2 | 2 | 1 | 3 | 1 | 2 |
| 2 | 2 | 2 | 1 | 3 | 1 | 2 |
| 2 | 2 | 2 | 1 | 3 | 1 | 2 |
| 2 | 2 | 2 | 1 | 3 | 1 | 2 |
| 2 | 2 | 2 | 1 | 3 | 1 | 2 |
| 2 | 2 | 2 | 1 | 3 | 1 | 2 |
| 2 | 2 | 2 | 1 | 3 | 1 | 2 |
| 4 | 4 | 4 | 3 | 5 | 1 | 4 |
| 4 | 4 | 4 | 3 | 5 | 1 | 4 |
| 4 | 4 | 4 | 3 | 5 | 1 | 4 |
| 4 | 4 | 4 | 3 | 5 | 1 | 4 |
| 4 | 4 | 4 | 3 | 5 | 1 | 4 |
| 4 | 4 | 4 | 3 | 5 | 1 | 4 |
| 4 | 4 | 4 | 3 | 5 | 1 | 4 |
| 4 | 4 | 4 | 3 | 5 | 1 | 4 |
| 4 | 4 | 4 | 3 | 5 | 2 | 4 |
| 4 | 4 | 4 | 3 | 5 | 2 | 4 |
| 4 | 4 | 4 | 3 | 5 | 2 | 4 |
| 4 | 4 | 4 | 3 | 5 | 2 | 4 |
| 4 | 4 | 4 | 3 | 5 | 2 | 4 |
| 4 | 4 | 4 | 3 | 5 | 2 | 4 |
| 4 | 4 | 4 | 3 | 5 | 2 | 4 |
| 1 | 1 | 1 | 0 | 1 | 0 | 2 |
| 1 | 1 | 1 | 0 | 1 | 0 | 2 |
| 1 | 1 | 1 | 0 | 1 | 0 | 2 |
| 1 | 1 | 1 | 0 | 1 | 0 | 2 |
| 1 | 1 | 1 | 0 | 1 | 0 | 2 |

| | | | | | | |
|---|---|---|---|---|---|---|
| 1 | 1 | 1 | 0 | 1 | 0 | 2 |
| 1 | 1 | 1 | 0 | 1 | 0 | 2 |
| 1 | 1 | 1 | 0 | 1 | 0 | 2 |
| 1 | 1 | 1 | 0 | 1 | 0 | 2 |
| 1 | 1 | 1 | 0 | 1 | 0 | 2 |
| 1 | 1 | 1 | 0 | 1 | 0 | 2 |
| 1 | 1 | 1 | 0 | 1 | 0 | 2 |
| 1 | 1 | 1 | 0 | 1 | 0 | 2 |
| 1 | 1 | 1 | 0 | 1 | 0 | 2 |
| 1 | 1 | 1 | 0 | 1 | 0 | 2 |
| 1 | 1 | 1 | 0 | 1 | 0 | 2 |
| 1 | 1 | 1 | 0 | 1 | 0 | 2 |
| 1 | 1 | 1 | 0 | 1 | 0 | 2 |
| 1 | 1 | 1 | 0 | 1 | 0 | 2 |
| 1 | 1 | 1 | 0 | 1 | 0 | 2 |
| 1 | 1 | 1 | 0 | 1 | 0 | 2 |
| 1 | 1 | 1 | 0 | 1 | 0 | 2 |
| 1 | 1 | 1 | 0 | 1 | 0 | 2 |
| 1 | 1 | 1 | 0 | 1 | 0 | 2 |
| 1 | 1 | 1 | 0 | 1 | 0 | 2 |
| 1 | 1 | 1 | 0 | 1 | 0 | 2 |
| 1 | 1 | 1 | 0 | 1 | 0 | 2 |
| 1 | 1 | 1 | 0 | 1 | 0 | 2 |
| 1 | 1 | 1 | 0 | 1 | 0 | 2 |
| 1 | 1 | 1 | 0 | 1 | 0 | 2 |
| 1 | 1 | 1 | 0 | 1 | 0 | 2 |
| 1 | 1 | 1 | 0 | 1 | 0 | 2 |
| 1 | 1 | 1 | 0 | 1 | 0 | 2 |
| 1 | 1 | 1 | 0 | 1 | 0 | 2 |
| 1 | 1 | 1 | 0 | 1 | 0 | 2 |
| 1 | 1 | 1 | 0 | 1 | 0 | 2 |
| 1 | 1 | 1 | 0 | 1 | 0 | 2 |
| 1 | 1 | 1 | 0 | 1 | 0 | 2 |
| 1 | 1 | 1 | 0 | 1 | 0 | 2 |
| 1 | 1 | 1 | 0 | 1 | 0 | 2 |
| 1 | 1 | 1 | 0 | 1 | 0 | 2 |
| 4 | 4 | 4 | 3 | 5 | 2 | 4 |
| 4 | 4 | 4 | 3 | 5 | 2 | 4 |
| 4 | 4 | 4 | 3 | 5 | 2 | 4 |
| 2 | 2 | 2 | 2 | 3 | 0 | 2 |

| | | | | | | |
|---|---|---|---|---|---|---|
| 2 | 2 | 2 | 2 | 3 | 0 | 2 |
| 2 | 2 | 2 | 2 | 3 | 0 | 2 |
| 2 | 2 | 2 | 2 | 3 | 0 | 2 |
| 2 | 2 | 2 | 2 | 3 | 0 | 2 |
| 2 | 2 | 2 | 1 | 3 | 2 | 4 |
| 4 | 4 | 4 | 2 | 5 | 1 | 2 |
| 4 | 4 | 4 | 2 | 5 | 1 | 2 |
| 4 | 4 | 4 | 2 | 5 | 1 | 2 |
| 4 | 4 | 4 | 2 | 5 | 1 | 2 |
| 4 | 4 | 4 | 2 | 5 | 1 | 2 |
| 4 | 4 | 4 | 2 | 5 | 1 | 2 |

| Financial Inequality |
| ---: |
| 2 |
| 2 |
| 2 |
| 2 |
| 2 |
| 2 |
| 2 |
| 2 |
| 2 |
| 2 |
| 2 |
| 2 |
| 2 |
| 2 |
| 2 |
| 2 |
| 2 |
| 2 |
| 2 |
| 2 |
| 2 |
| 2 |
| 2 |
| 2 |
| 2 |
| 2 |
| 2 |
| 2 |
| 2 |
| 2 |
| 2 |
| 2 |
| 2 |
| 2 |
| 2 |
| 2 |
| 2 |
| 2 |
| 2 |
| 2 |
| 2 |
| 2 |
| 2 |
| 2 |
| 2 |
| 2 |
| 2 |

| |
|---|
| 2 |
| 2 |
| 2 |
| 2 |
| 2 |
| 2 |
| 2 |
| 2 |
| 2 |
| 2 |
| 2 |
| 2 |
| 2 |
| 2 |
| 2 |
| 2 |
| 2 |
| 2 |
| 2 |
| 2 |
| 2 |
| 2 |
| 2 |
| 2 |
| 2 |
| 2 |
| 2 |
| 2 |
| 2 |
| 2 |
| 2 |
| 2 |
| 2 |
| 2 |
| 2 |
| 2 |
| 2 |
| 2 |
| 2 |
| 2 |
| 2 |
| 2 |

| |
|---|
| 2 |
| 2 |
| 2 |
| 2 |
| 2 |
| 2 |
| 2 |
| 2 |
| 2 |
| 2 |
| 2 |
| 2 |
| 2 |
| 2 |
| 2 |
| 2 |
| 2 |
| 2 |
| 2 |
| 2 |
| 2 |
| 2 |
| 2 |
| 2 |
| 2 |
| 2 |
| 2 |
| 2 |
| 2 |
| 2 |
| 2 |
| 2 |
| 2 |
| 2 |
| 2 |
| 2 |
| 2 |
| 2 |
| 2 |
| 2 |

| |
|---|
| 2 |
| 2 |
| 2 |
| 2 |
| 2 |
| 2 |
| 2 |
| 2 |
| 2 |
| 2 |
| 2 |
| 2 |
| 2 |
| 2 |
| 2 |
| 2 |
| 2 |
| 2 |
| 2 |
| 2 |
| 2 |
| 2 |
| 2 |
| 2 |
| 2 |
| 2 |
| 2 |
| 2 |
| 2 |
| 2 |
| 2 |
| 2 |
| 2 |
| 2 |
| 2 |
| 2 |
| 2 |
| 2 |
| 2 |
| 2 |

| |
|---|
| 2 |
| 2 |
| 2 |
| 2 |
| 2 |
| 2 |
| 2 |
| 2 |
| 2 |
| 2 |
| 2 |

| Index | Company Name | Code |
|---|---|---|
| STAR 50 | 3PEAK INCORPORATED | 7E+05 |
| STAR 50 | ACM Research (Shanghai), Inc. | 7E+05 |
| STAR 50 | Advanced Micro-Fabrication Equipment Inc. China | 7E+05 |
| STAR 50 | Amlogic (Shanghai) Co., Ltd. | 7E+05 |
| STAR 50 | ASR Microelectronics Co., Ltd | 7E+05 |
| STAR 50 | AVIC (CHENGDU) UAS CO., LTD. | 7E+05 |
| STAR 50 | Beijing Kingsoft Office Software, Inc. | 7E+05 |
| STAR 50 | Beijing Roborock Technology Co ., Ltd. | 7E+05 |
| STAR 50 | Bloomage Biotechnology Corporation Limited | 7E+05 |
| STAR 50 | Cambricon Technologies Corporation Limited | 7E+05 |
| STAR 50 | Cathay Biotech Inc. | 7E+05 |
| STAR 50 | China Railway Signal & Communication Corporation Limit | 7E+05 |
| STAR 50 | China Resources Microelectronics Limited | 7E+05 |
| STAR 50 | Everdisplay Optronics (Shanghai) Co., Ltd. | 7E+05 |
| STAR 50 | GalaxyCore Inc. | 7E+05 |
| STAR 50 | GoodWe Technologies Co.,Ltd. | 7E+05 |
| STAR 50 | GUOBO ELECTRONICS CO., LTD. | 7E+05 |
| STAR 50 | Hoymiles Power Electronics Inc. | 7E+05 |
| STAR 50 | HUNAN CHANGYUAN LICO CO.,LTD. | 7E+05 |
| STAR 50 | Hwatsing Technology Co.,Ltd. | 7E+05 |
| STAR 50 | Hygon Information Technology Co., Ltd. | 7E+05 |
| STAR 50 | iRay Technology Company Limited | 7E+05 |
| STAR 50 | Jinko Solar Co., Ltd. | 7E+05 |
| STAR 50 | Loongson Technology Corporation Limited | 7E+05 |
| STAR 50 | MGI Tech Co., Ltd. | 7E+05 |
| STAR 50 | Montage Technology Co., Ltd. | 7E+05 |
| STAR 50 | National Silicon Industry Group Co., Ltd. | 7E+05 |
| STAR 50 | Ningbo Ronbay New Energy Technology Co., Ltd. | 7E+05 |
| STAR 50 | Piotech Inc. | 7E+05 |
| STAR 50 | Pylon Technologies Co., Ltd. | 7E+05 |
| STAR 50 | Qi An Xin Technology Group Inc. | 7E+05 |
| STAR 50 | Sany Heavy Energy Co., Ltd. | 7E+05 |
| STAR 50 | Semiconductor Manufacturing International Corporation | 7E+05 |
| STAR 50 | Shanghai Friendess Electronic Technology Corporation Lin | 7E+05 |
| STAR 50 | Shanghai Fudan Microelectronics Group CO.,LTD. | 7E+05 |
| STAR 50 | Shanghai Junshi Biosciences Co., Ltd. | 7E+05 |
| STAR 50 | Shanghai United Imaging Healthcare Co., Ltd. | 7E+05 |
| STAR 50 | SICC Co., Ltd. | 7E+05 |
| STAR 50 | Sichuan Biokin Pharmaceutical Co.,Ltd. | 7E+05 |
| STAR 50 | SUPCON Technology Co., Ltd. | 7E+05 |
| STAR 50 | Suzhou Novosense Microelectronics Co., Ltd. | 7E+05 |
| STAR 50 | Tianneng Battery Group Co., Ltd. | 7E+05 |
| STAR 50 | Transsion Holdings LTD.CO | 7E+05 |
| STAR 50 | Trina Solar Co., Ltd. | 7E+05 |
| STAR 50 | VeriSilicon Microelectronics (Shanghai) Co., Ltd. | 7E+05 |
| STAR 50 | Western Superconducting Technologies Co.,Ltd. | 7E+05 |
| STAR 50 | Xinjiang Daqo New Energy Co.,Ltd | 7E+05 |

| | | |
|---|---|---|
| STAR 50 | Yuneng Technology Co., Ltd. | 7E+05 |
| STAR 50 | Zhongfu Shenying Carbon Fiber Co., Ltd | 7E+05 |
| STAR 50 | Zhuzhou CRRC Times Electric Co., Ltd. | 7E+05 |

| Sector | Comments |
| --- | --- |
| Electronics & Semiconductor Manufacturing | |
| Electronics & Semiconductor Manufacturing | |
| Electronics & Semiconductor Manufacturing | |
| Electronics & Semiconductor Manufacturing | |
| Telecommunication services (including wireless) | |
| Automotive, Electrical Equipment & Machinery Production | |
| Offices & Professional Services | |
| Appliances & General Goods Manufacturing | |
| Health Care, Pharmaceuticals and Biotechnology | |
| Electronics & Semiconductor Manufacturing | |
| Health Care, Pharmaceuticals and Biotechnology | |
| Transportation Services | |
| Electronics & Semiconductor Manufacturing | |
| Electronics & Semiconductor Manufacturing | |
| Electronics & Semiconductor Manufacturing | |
| Electronics & Semiconductor Manufacturing | |
| Telecommunication services (including wireless) | |
| Electronics & Semiconductor Manufacturing | |
| Electronics & Semiconductor M | Battery |
| Electronics & Semiconductor Manufacturing | |
| Electronics & Semiconductor Manufacturing | |
| Health Care, Pharmaceuticals and Biotechnology | |
| Electronics & Semiconductor M | Solar |
| Electronics & Semiconductor Manufacturing | |
| Health Care, Pharmaceuticals and Biotechnology | |
| Electronics & Semiconductor Manufacturing | |
| Electronics & Semiconductor Manufacturing | |
| Electronics & Semiconductor M | Batteries |
| Electronics & Semiconductor Manufacturing | |
| Electronics & Semiconductor M | Batteries |
| Other (Average of all sectors) | |
| Electric Energy Production - Solar, Wind | |
| Electronics & Semiconductor Manufacturing | |
| Automotive, Electrical Equipment & Machinery Production | |
| Electronics & Semiconductor Manufacturing | |
| Health Care, Pharmaceuticals and Biotechnology | |
| Health Care, Pharmaceuticals and Biotechnology | |
| Electronics & Semiconductor Manufacturing | |
| Health Care, Pharmaceuticals and Biotechnology | |
| Electronics & Semiconductor Manufacturing | |
| Electronics & Semiconductor Manufacturing | |
| Electronics & Semiconductor M | Battery |
| Electronics & Semiconductor Manufacturing | |
| Electronics & Semiconductor Manufacturing | |
| Electronics & Semiconductor Manufacturing | |
| Electronics & Semiconductor Manufacturing | |
| Chemicals & Other Materials Production | |

Electronics & Semiconductor Manufacturing
Chemicals & Other Materials Production
Automotive, Electrical Equipment & Machinery Production

| Index | Company Name | Code |
|---|---|---|
| Shenzhen 129 | Aier Eye Hospital | 300015 |
| Shenzhen 118 | Arawana | 300999 |
| Shenzhen 134 | Asymchem | 2821 |
| Shenzhen 173 | Avary Holding | 2938 |
| Shenzhen 146 | AVIC XAC | 768 |
| Shenzhen 123 | Bank of Ningbo | 2142 |
| Shenzhen 194 | BNBMPLC | 786 |
| Shenzhen 159 | BOE | 725 |
| Shenzhen 120 | Botanee | 300957 |
| Shenzhen 102 | BYD | 2594 |
| Shenzhen 152 | CATL | 300750 |
| Shenzhen 132 | CCHN | 661 |
| Shenzhen 167 | CCTC | 300408 |
| Shenzhen 181 | Century Huatong | 2602 |
| Shenzhen 199 | CGN | 3816 |
| Shenzhen 103 | Changan Automobile | 625 |
| Shenzhen 186 | CITIC Steel | 708 |
| Shenzhen 196 | CMSK | 1979 |
| Shenzhen 189 | CNGR | 300919 |
| Shenzhen 127 | CNPCCCL | 617 |
| Shenzhen 172 | CVTE | 2841 |
| Shenzhen 170 | Dahua Inc | 2236 |
| Shenzhen 190 | Eastern Shenghong | 301 |
| Shenzhen 121 | EastMoney | 300059 |
| Shenzhen 187 | ENERGY TECHNOLOGY | 2812 |
| Shenzhen 156 | EVE | 300014 |
| Shenzhen 107 | Focus Media | 2027 |
| Shenzhen 188 | Ganfeng Lithium | 2460 |
| Shenzhen 124 | GF Securities | 776 |
| Shenzhen 178 | Glodon | 2410 |
| Shenzhen 160 | Goertek | 2241 |
| Shenzhen 151 | Goldwind | 2202 |
| Shenzhen 101 | Gree | 651 |
| Shenzhen 174 | Guide Infrared | 2414 |
| Shenzhen 119 | Gujing Distillery | 596 |
| Shenzhen 126 | Guosen Securities | 2736 |
| Shenzhen 161 | Guoxin Micro | 2049 |
| Shenzhen 117 | Haid Group | 2311 |
| Shenzhen 168 | Han's Laser | 2008 |
| Shenzhen 140 | HD Medicine | 963 |
| Shenzhen 158 | Hikvision | 2415 |
| Shenzhen 141 | Hualan Biological | 2007 |
| Shenzhen 109 | Huali Group | 300979 |
| Shenzhen 177 | Iflytek | 2230 |
| Shenzhen 136 | Imeik | 300896 |
| Shenzhen 153 | Inovance | 300124 |
| Shenzhen 150 | JA Solar | 2459 |

| | | |
|---|---|---|
| Shenzhen 166 | JONHON | 2179 |
| Shenzhen 138 | Kangtai Biological | 300601 |
| Shenzhen 193 | LBG | 2601 |
| Shenzhen 171 | LCXX | 977 |
| Shenzhen 154 | Lead Intelligent | 300450 |
| Shenzhen 169 | Lens | 300433 |
| Shenzhen 143 | Lepu Medical | 300003 |
| Shenzhen 157 | Luxshare-ICT | 2475 |
| Shenzhen 115 | Luzhou Lao Jiao | 568 |
| Shenzhen 183 | LY iTECH | 2600 |
| Shenzhen 108 | Mango | 300413 |
| Shenzhen 165 | Maxscend | 300782 |
| Shenzhen 100 | Midea Group | 333 |
| Shenzhen 128 | Mindray | 300760 |
| Shenzhen 110 | Muyuan | 2714 |
| Shenzhen 163 | NAURA | 2371 |
| Shenzhen 113 | New Hope | 876 |
| Shenzhen 137 | NHU | 2001 |
| Shenzhen 105 | OCT Holding | 69 |
| Shenzhen 106 | OFFCN EDU | 2607 |
| Shenzhen 185 | Oriental Yuhong Waterproof Technolo | 2271 |
| Shenzhen 122 | PAB | 1 |
| Shenzhen 139 | Pharmaron | 300759 |
| Shenzhen 191 | QHSLI | 792 |
| Shenzhen 176 | RoyalFlush Info | 300033 |
| Shenzhen 192 | RSPC | 2493 |
| Shenzhen 179 | Sangfor | 300454 |
| Shenzhen 144 | Sanhua | 2050 |
| Shenzhen 180 | Sanqi Huyu | 2555 |
| Shenzhen 155 | SF Holding | 2352 |
| Shenzhen 142 | Shanghai RAAS | 2252 |
| Shenzhen 175 | Shennan Circuits | 2916 |
| Shenzhen 125 | Shenwan Hongyuan | 166 |
| Shenzhen 112 | Shuanghui | 895 |
| Shenzhen 149 | Sungrow Power Supply | 300274 |
| Shenzhen 104 | Supor | 2032 |
| Shenzhen 164 | TCL Tech. | 100 |
| Shenzhen 133 | Tigermed | 300347 |
| Shenzhen 184 | Tinci | 2709 |
| Shenzhen 162 | TJSEMI | 2129 |
| Shenzhen 182 | UNIS | 938 |
| Shenzhen 195 | Vanke-A | 2 |
| Shenzhen 131 | Walvax | 300142 |
| Shenzhen 145 | Weichai Power | 338 |
| Shenzhen 111 | Wens | 300498 |
| Shenzhen 114 | Wuliangye | 858 |
| Shenzhen 148 | XCMG | 425 |
| Shenzhen 116 | Yanghe | 2304 |
| Shenzhen 198 | Yealink Network | 300628 |
| Shenzhen 135 | Yunnan Baiyao | 538 |
| Shenzhen 130 | Zhifei-Biol | 300122 |
| Shenzhen 147 | Zoomlion | 157 |
| Shenzhen 197 | ZTE | 63 |

| Sector | Comments |
|---|---|
| Health Care, Pharmaceuticals and Biotechnology | |
| Food & Beverage Production | |
| Health Care, Pharmaceuticals and Biotechnology | |
| Electronics & Semiconductor Manufacturing | |
| Automotive, Electrical Equipment & Machinery Production | |
| Offices & Professional Services | |
| Other (Average of all sectors) | Unsure |
| Electronics & Semiconductor Manufacturing | |
| Health Care, Pharmaceuticals and Biotechnology | |
| Automotive, Electrical Equipment & Machinery Production | |
| Electronics & Semiconductor Manufacturing | |
| Health Care, Pharmaceuticals and Biotechnology | |
| Electronics & Semiconductor Manufacturing | |
| Other (Average of all sectors) | auto parts, Internet games, and cloud data |
| Electric Energy Production - Combustion (Biomass, Coal, Gas, Nuclear, Oil), Geothermal Energy | |
| Automotive, Electrical Equipment & Machinery Production | |
| Construction Materials | |
| Land Development & Construction | |
| Metals & Mining | |
| Other (Average of all sectors) | |
| Electronics & Semiconductor Manufacturing | |
| Electronics & Semiconductor Manufacturing | |
| Textiles, Apparel & Luxury Good Production | |
| Offices & Professional Services | Bank |
| Electric Energy Production - Combustion (Biomass, Coal, Gas, Nuclear, Oil), Geothermal Energy | |
| Automotive, Electrical Equipment & Machinery P | Batteries |
| Offices & Professional Services | Advertizing |
| Metals & Mining | |
| Other (Average of all sectors) | |
| Other (Average of all sectors) | |
| Electronics & Semiconductor Manufacturing | |
| Electric Energy Production - Solar, Wind | |
| Appliances & General Goods Manufacturing | |
| Electronics & Semiconductor Manufacturing | |
| Food & Beverage Production | |
| Other (Average of all sectors) | |
| Electronics & Semiconductor Manufacturing | |
| Food & Beverage Production | |
| Electronics & Semiconductor Manufacturing | |
| Health Care, Pharmaceuticals and Biotechnology | |
| Electronics & Semiconductor Manufacturing | |
| Health Care, Pharmaceuticals and Biotechnology | |
| Textiles, Apparel & Luxury Good Production | |
| Other (Average of all sectors) | |
| Health Care, Pharmaceuticals and Biotechnology | |
| Electronics & Semiconductor Manufacturing | |
| Electric Energy Production - Solar, Wind | |

| | |
|---|---|
| Electronics & Semiconductor Manufacturing | |
| Health Care, Pharmaceuticals and Biotechnology | |
| Other (Average of all sectors) | Unsure |
| Electronics & Semiconductor Manufacturing | |
| Electronics & Semiconductor Manufacturing | |
| Electronics & Semiconductor Manufacturing | |
| Health Care, Pharmaceuticals and Biotechnology | |
| Electronics & Semiconductor Manufacturing | |
| Food & Beverage Production | |
| Other (Average of all sectors) | Unsure |
| Offices & Professional Services | Internet new media industry |
| Electronics & Semiconductor Manufacturing | |
| Appliances & General Goods Manufacturing | |
| Health Care, Pharmaceuticals and Biotechnology | |
| Agriculture (animal products) | |
| Electronics & Semiconductor Manufacturing | |
| Agriculture (plant products) | And chickens |
| Health Care, Pharmaceuticals and Biotechnology | |
| Land Development & Construction | Tourism and real estate sectors |
| Offices & Professional Services | Education |
| Construction Materials | |
| Offices & Professional Services | Bank |
| Health Care, Pharmaceuticals and Biotechnology | |
| Other (Average of all sectors) | Unsure |
| Offices & Professional Services | |
| Chemicals & Other Materials Production | |
| Other (Average of all sectors) | |
| Appliances & General Goods Manufacturing | |
| Other (Average of all sectors) | |
| Food Retailing | |
| Health Care, Pharmaceuticals and Biotechnology | |
| Electronics & Semiconductor Manufacturing | |
| Other (Average of all sectors) | |
| Agriculture (animal products) | |
| Electric Energy Production - Solar, Wind | |
| General or Speciality Retailing | |
| Electronics & Semiconductor Manufacturing | |
| Health Care, Pharmaceuticals and Biotechnology | |
| Chemicals & Other Materials Production | |
| Electronics & Semiconductor Manufacturing | |
| Other (Average of all sectors) | |
| Land Development & Construction | |
| Health Care, Pharmaceuticals and Biotechnology | |
| Automotive, Electrical Equipment & Machinery Production | |
| Agriculture (animal products) | |
| Food & Beverage Production | |
| Construction Materials | |
| Food & Beverage Production | |
| Telecommunication services (including wireless) | |
| Health Care, Pharmaceuticals and Biotechnology | |
| Health Care, Pharmaceuticals and Biotechnology | |
| Construction Materials | |
| Telecommunication services (including wireless) | |

| Index | Company Name | Code | Sector |
|---|---|---|---|
| ChiNex Index (CNXT) | Aier Eye Hospital Group Co Ltd | BBG00F138RQ2 | Health Care, Pharmaceutica |
| ChiNex Index (CNXT) | Anhui Anke Biotechnology Group Co Ltd | BBG00F138QW7 | Health Care, Pharmaceutica |
| ChiNex Index (CNXT) | Anker Innovations Technology Co Ltd | BBG011C97H08 | Electronics & Semiconducto |
| ChiNex Index (CNXT) | Beijing Compass Technology Developmer | BBG00YG5DBJ7 | Other (Average of all sectors |
| ChiNex Index (CNXT) | Beijing Easpring Material Technology Co | BBG00FNDHC25 | Electronics & Semiconducto |
| ChiNex Index (CNXT) | Beijing Enlight Media Co Ltd | BBG00F13CG90 | Offices & Professional Servic |
| ChiNex Index (CNXT) | Beijing Originwater Technology Co Ltd | BBG00F1391G9 | Water utilities / Water Servic |
| ChiNex Index (CNXT) | Beijing Sinnet Technology Co Ltd | BBG00F13DSG5 | Other (Average of all sectors |
| ChiNex Index (CNXT) | Betta Pharmaceuticals Co Ltd | BBG00H1LL7F9 | Health Care, Pharmaceutica |
| ChiNex Index (CNXT) | Bgi Genomics Co Ltd | BBG00LBJFCZ8 | Health Care, Pharmaceutica |
| ChiNex Index (CNXT) | By-Health Co Ltd | BBG00F13B078 | Health Care, Pharmaceutica |
| ChiNex Index (CNXT) | Canmax Technologies Co Ltd | BBG00YG5D7D2 | Electronics & Semiconducto |
| ChiNex Index (CNXT) | Centre Testing International Group Co L | BBG00F138RK8 | Appliances & General Goods |
| ChiNex Index (CNXT) | Chaozhou Three-Circle Group Co Ltd | BBG00F13DSS2 | Electronics & Semiconducto |
| ChiNex Index (CNXT) | China Resources Boya Bio-Pharmaceutica | BBG00F13D708 | Health Care, Pharmaceutica |
| ChiNex Index (CNXT) | Chongqing Zhifei Biological Products Co | BBG00F139TV1 | Health Care, Pharmaceutica |
| ChiNex Index (CNXT) | Cngr Advanced Material Co Ltd | BBG013Y510B9 | Electronics & Semiconducto |
| ChiNex Index (CNXT) | Contemporary Amperex Technology Co L | BBG00MYKWDB: | Electronics & Semiconducto |
| ChiNex Index (CNXT) | Dian Diagnostics Group Co Ltd | BBG00F13CFR2 | Health Care, Pharmaceutica |
| ChiNex Index (CNXT) | Dongguan Yiheda Automation Co Ltd | BBG017ZPHSQ1 | Automotive, Electrical Equipı |
| ChiNex Index (CNXT) | East Group Co Ltd | BBG00F13DS48 | Electronics & Semiconducto |
| ChiNex Index (CNXT) | East Money Information Co Ltd | BBG00F138WK7 | Offices & Professional Servic |
| ChiNex Index (CNXT) | Empyrean Technology Co Ltd | BBG01FRBBM91 | Electronics & Semiconducto |
| ChiNex Index (CNXT) | Eoptolink Technology Inc Ltd | BBG00FNDHXJ1 | Other (Average of all sectors |
| ChiNex Index (CNXT) | Eve Energy Co Ltd | BBG00F138RN5 | Automotive, Electrical Equipı |
| ChiNex Index (CNXT) | Gaona Aero Material Co Ltd | BBG00F138V38 | Automotive, Electrical Equipı |
| ChiNex Index (CNXT) | Ginlong Technologies Co Ltd | BBG00YG5D9P5 | Electronics & Semiconducto |
| ChiNex Index (CNXT) | Guangzhou Great Power Energy & Techn | BBG00FNDHRG7 | Electronics & Semiconducto |
| ChiNex Index (CNXT) | Hangzhou Chang Chuan Technology Co Lt | BBG00VC81FX9 | Electronics & Semiconducto |
| ChiNex Index (CNXT) | Hangzhou Tigermed Consulting Co Ltd | BBG00F13DR59 | Health Care, Pharmaceutica |
| ChiNex Index (CNXT) | Hithink Royalflush Information Network | BBG00F138V10 | Offices & Professional Servic |
| ChiNex Index (CNXT) | Hualan Biological Vaccine Inc | BBG01BWNBNP8 | Health Care, Pharmaceutica |
| ChiNex Index (CNXT) | Huali Industrial Group Co Ltd | BBG013Y513M1 | Textiles, Apparel & Luxury G |
| ChiNex Index (CNXT) | Huaxia Eye Hospital Group Co Ltd | BBG01FRBBLP5 | Health Care, Pharmaceutica |
| ChiNex Index (CNXT) | Hubei Dinglong Co Ltd | BBG00F138W72 | Electronics & Semiconducto |
| ChiNex Index (CNXT) | Hubei Feilihua Quartz Glass Co Ltd | BBG00VC817P6 | Chemicals & Other Materials |
| ChiNex Index (CNXT) | Hunan Yuneng New Energy Battery Mate | BBG01G92FS65 | Electronics & Semiconducto |
| ChiNex Index (CNXT) | Imeik Technology Development Co Ltd | BBG011C97SS4 | Health Care, Pharmaceutica |
| ChiNex Index (CNXT) | Ingenic Semiconductor Co Ltd | BBG00FNDHJJ2 | Electronics & Semiconducto |
| ChiNex Index (CNXT) | Isoftstone Information Technology Grour | BBG01BWNBPZ2 | Offices & Professional Servic |
| ChiNex Index (CNXT) | Jafron Biomedical Co Ltd | BBG00LBJC657 | Health Care, Pharmaceutica |
| ChiNex Index (CNXT) | Jl Mag Rare-Earth Co Ltd | BBG00PF5F1K5 | Chemicals & Other Materials |
| ChiNex Index (CNXT) | Konfoong Materials International Co Ltd | BBG00JN03482 | Chemicals & Other Materials |
| ChiNex Index (CNXT) | Kunlun Tech Co Ltd | BBG00F13DSY5 | Other (Average of all sectors |
| ChiNex Index (CNXT) | Lens Technology Co Ltd | BBG00F13DT73 | Electronics & Semiconducto |
| ChiNex Index (CNXT) | Lepu Medical Technology Beijing Co Ltd | BBG00F138PZ6 | Health Care, Pharmaceutica |
| ChiNex Index (CNXT) | Leyard Optoelectronic Co Ltd | BBG00F13D7Y1 | Automotive, Electrical Equipı |

| | | | |
|---|---|---|---|
| ChiNex Index (CNXT) | Longshine Technology Group Co Ltd | BBG00PF5DZX8 | Other (Average of all sectors |
| ChiNex Index (CNXT) | Mango Excellent Media Co Ltd | BBG00F13DSW7 | Offices & Professional Servic |
| ChiNex Index (CNXT) | Maxscend Microelectronics Co Ltd | BBG00VC80L30 | Electronics & Semiconductor |
| ChiNex Index (CNXT) | Ovctek China Inc | BBG00MYKW8V2 | Health Care, Pharmaceutica |
| ChiNex Index (CNXT) | Pharmaron Beijing Co Ltd | BBG00R08YHZ9 | Health Care, Pharmaceutica |
| ChiNex Index (CNXT) | Porton Pharma Solutions Ltd | BBG00F13DRR5 | Health Care, Pharmaceutica |
| ChiNex Index (CNXT) | Qingdao Tgood Electric Co Ltd | BBG00F138PD0 | Automotive, Electrical Equipı |
| ChiNex Index (CNXT) | Risen Energy Co Ltd | BBG00F139TS5 | Electric Energy Production - |
| ChiNex Index (CNXT) | Sangfor Technologies Inc | BBG00MYKW735 | Other (Average of all sectors |
| ChiNex Index (CNXT) | Semitronix Corp | BBG01FR9T987 | Electronics & Semiconductor |
| ChiNex Index (CNXT) | Sg Micro Corp | BBG00MYKWCC4 | Automotive, Electrical Equipı |
| ChiNex Index (CNXT) | Shandong Sinocera Functional Material C | BBG00FNDHL98 | Appliances & General Goods |
| ChiNex Index (CNXT) | Shandong Weifang Rainbow Chemical Co | BBG017ZPJGH5 | Chemicals & Other Materials |
| ChiNex Index (CNXT) | Shenzhen Capchem Technology Co Ltd | BBG00F138V83 | Chemicals & Other Materials |
| ChiNex Index (CNXT) | Shenzhen Dynanonic Co Ltd | BBG013Y4ZS51 | Electronics & Semiconductor |
| ChiNex Index (CNXT) | Shenzhen Inovance Technology Co Ltd | BBG00F139TZ7 | Electronics & Semiconductor |
| ChiNex Index (CNXT) | Shenzhen Kangtai Biological Products Co | BBG00LBJC7X4 | Health Care, Pharmaceutica |
| ChiNex Index (CNXT) | Shenzhen Longsys Electronics Co Ltd | BBG01FRBBRP2 | Electronics & Semiconductor |
| ChiNex Index (CNXT) | Shenzhen Mindray Bio-Medical Electroni | BBG00MYKWDD1 | Health Care, Pharmaceutica |
| ChiNex Index (CNXT) | Shenzhen New Industries Biomedical Eng | BBG011C97D07 | Health Care, Pharmaceutica |
| ChiNex Index (CNXT) | Shenzhen Sc New Energy Technology Cor | BBG00PF5F036 | Electric Energy Production - |
| ChiNex Index (CNXT) | Shenzhen Senior Technology Material Co | BBG00MYKW8B4 | Electronics & Semiconducto |
| ChiNex Index (CNXT) | Shenzhen Sunway Communication Co Ltc | BBG00F139VD6 | Electronics & Semiconductor |
| ChiNex Index (CNXT) | Sinofibers Technology Co Ltd | BBG00VC80KM1 | Telecommunication services |
| ChiNex Index (CNXT) | Songcheng Performance Development Co | BBG00F13B032 | Offices & Professional Servic |
| ChiNex Index (CNXT) | Sonoscape Medical Corp | BBG00MYKWC25 | Health Care, Pharmaceutica |
| ChiNex Index (CNXT) | Sungrow Power Supply Co Ltd | BBG00F13CY28 | Electric Energy Production - |
| ChiNex Index (CNXT) | Sunresin New Materials Co Ltd | BBG00MYKW7J8 | Chemicals & Other Materials |
| ChiNex Index (CNXT) | Sunwoda Electronic Co Ltd | BBG00F13BZ08 | Electronics & Semiconducto |
| ChiNex Index (CNXT) | Suzhou Maxwell Technologies Co Ltd | BBG00R2FQVG0 | Electronics & Semiconductor |
| ChiNex Index (CNXT) | Suzhou Tfc Optical Communication Co Lt | BBG00R2FQSB2 | Telecommunication services |
| ChiNex Index (CNXT) | Thunder Software Technology Co Ltd | BBG00F13F1V5 | Offices & Professional Servic |
| ChiNex Index (CNXT) | Tofflon Science & Technology Group Co L | BBG00F13BGY3 | Health Care, Pharmaceutica |
| ChiNex Index (CNXT) | Walvax Biotechnology Co Ltd | BBG00F13B014 | Health Care, Pharmaceutica |
| ChiNex Index (CNXT) | Weihai Guangwei Composites Co Ltd | BBG00LBJGFK6 | Chemicals & Other Materials |
| ChiNex Index (CNXT) | Wens Foodstuffs Group Co Ltd | BBG00F13F476 | Agriculture (animal products) |
| ChiNex Index (CNXT) | Winner Medical Co Ltd | BBG011C97MD3 | Health Care, Pharmaceutica |
| ChiNex Index (CNXT) | Winning Health Technology Group Co Ltd | BBG00F13CGF3 | Offices & Professional Servic |
| ChiNex Index (CNXT) | Wuhan Dr Laser Technology Corp Ltd | BBG00VC80K69 | Automotive, Electrical Equipı |
| ChiNex Index (CNXT) | Wuhan Jingce Electronic Group Co Ltd | BBG00LBJC7L7 | Electronics & Semiconductor |
| ChiNex Index (CNXT) | Wuxi Lead Intelligent Equipment Co Ltd | BBG00H1LL778 | Electronics & Semiconductor |
| ChiNex Index (CNXT) | Xi'An Triangle Defense Co Ltd | BBG00VC80JX2 | Automotive, Electrical Equipı |
| ChiNex Index (CNXT) | Yangling Metron New Material Inc | BBG011C97GP3 | Automotive, Electrical Equipı |
| ChiNex Index (CNXT) | Yangzhou Yangjie Electronic Technology | BBG00F13DS20 | Electronics & Semiconductor |
| ChiNex Index (CNXT) | Yealink Network Technology Corp Ltd | BBG00JN03348 | Telecommunication services |
| ChiNex Index (CNXT) | Yihai Kerry Arawana Holdings Co Ltd | BBG00YG5DCM1 | Food & Beverage Production |
| ChiNex Index (CNXT) | Yunnan Botanee Bio-Technology Group C | BBG013Y512B5 | Health Care, Pharmaceutica |
| ChiNex Index (CNXT) | Zhejiang Jingsheng Mechanical & Electri | BBG00F13DKX3 | Electronics & Semiconductor |

| | | | |
|---|---|---|---|
| ChiNex Index (CNXT) | Zhejiang Wolwo Bio-Pharmaceutical Co L | BBG00LBJBZ45 | Health Care, Pharmaceutica |
| ChiNex Index (CNXT) | Zhongji Innolight Co Ltd | BBG00JMZYLD8 | Telecommunication services |
| ChiNex Index (CNXT) | Zhuzhou Hongda Electronics Corp Ltd | BBG00LBJGMF7 | Electronics & Semiconducto |

**Comments**
Battery
ls and Biotechnology
r Manufacturing
s)
Battery
ces
e Providers
s)
ls and Biotechnology
ls and Biotechnology
ls and Biotechnology
Battery
s Manufacturing
r Manufacturing
ls and Biotechnology
ls and Biotechnology
Battery
r Manufacturing
ls and Biotechnology
ment & Machinery Production
Battery
ces
r Manufacturing
s)
ment & Machinery Production
ment & Machinery Production
r Manufacturing
Battery
r Manufacturing
ls and Biotechnology
ces
ls and Biotechnology
ood Production
ls and Biotechnology
r Manufacturing
s Production
Battery
ls and Biotechnology
r Manufacturing
ces
ls and Biotechnology
s Production
s Production
s)
r Manufacturing
ls and Biotechnology
ment & Machinery Production

;)
ces
r Manufacturing
ls and Biotechnology
ls and Biotechnology
ls and Biotechnology
nent & Machinery Production
Solar, Wind
;)
r Manufacturing
nent & Machinery Production
s Manufacturing
; Production
; Production
r Manufacturing
r Manufacturing
ls and Biotechnology
r Manufacturing
ls and Biotechnology
ls and Biotechnology
Solar, Wind
Battery
r Manufacturing
(including wireless)
ces
ls and Biotechnology
Solar, Wind
; Production
Battery
r Manufacturing
(including wireless)
ces
ls and Biotechnology
ls and Biotechnology
; Production
)
ls and Biotechnology
ces
nent & Machinery Production
r Manufacturing
r Manufacturing
nent & Machinery Production
nent & Machinery Production
r Manufacturing
(including wireless)
n
ls and Biotechnology
r Manufacturing

[illegible]ls and Biotechnology
[illegible] (including wireless)
[illegible] Manufacturing

| Sectors |
| --- |
| Agriculture (animal products) |
| Agriculture (plant products) |
| Appliances & General Goods Manufacturing |
| Production |
| Chemicals & Other Materials Production |
| Construction Materials |
| Coal, Gas, Nuclear, Oil), Geothermal Energy |
| Electric Energy Production – Hydropower |
| Electric Energy Production - Solar, Wind |
| Electronics & Semiconductor Manufacturing |
| Fishing and aquaculture |
| Food & Beverage Production |
| Food Retailing |
| General or Speciality Retailing |
| Health Care, Pharmaceuticals and Biotechnology |
| Hospitality Services |
| Land Development & Construction |
| Metals & Mining |
| Offices & Professional Services |
| Oil, Gas & Consumable Fuels |
| Paper & Forest Product Production |
| Telecommunication services (including wireless) |
| Textiles, Apparel & Luxury Good Production |
| Transportation Services |
| Water utilities / Water Service Providers |
| Other (Average of all sectors) |
