# Supplementary material for: Towards nature-related risk disclosures in China
Source: Bioscience. 2026 Jun 18;76(7):591–600. doi: 10.1093/biosci/biag071 (PMC13377638; doi:10.1093/biosci/biag071)
Supplement: biag071_Supplemental_Files [file biag071_supplemental_files.zip › Supp_Information.docx]

| TNFD Recommended ‘LEAP’ Framework | | |
| --- | --- | --- |
| Definition | Enabler | Barrier |
| Locate (Interface with nature) | | |
| The location of business operations and its interface with nature. Which locations interact with high value conservation areas of areas which also overlap with other sectors or businesses. | Published vegetation and biome map of China ^1^ Protected areas and parks Maps from national ecosystem assessments and Red List of Ecosystems assessments ^2–4^National ecosystem assessmentsChina’s ecological red line map and main functional zoning map ^5^Information about the sectors that consume the largest amounts of land, water, biodiversity, energy (e.g., agriculture, manufacturing) ^6^Energy sectors and raw material sectors usually interface with nature | Unclear specificity of business footprint% of State ownership of listed companies is relatively highIndirect effect alone the supply chain is invisible but important. |
| Evaluate (Dependencies & impacts) | | |
| Identify relevant environmental assets and ecosystem services, as well as the dependencies and impacts of the business at each priority location. | Many ecosystem services assessments conductedStandardised legal disclosure of corporate environmental information (ESG)Eco-product identification Information about the input (e.g., land and water) and output (products, pollutants, waste) of businessesResource efficiency, e.g., land use and water, of industrial production ^7^Spillover effect identification and measurementNew quality productivityEnvironmental footprints on the production sideClimate change biodiversity | No platform that collates high-quality ecosystem services assessmentsUniform assessment for nature-related risks is lacking, which impedes the comparison and cross-border utilizationMost of the corporates who can use the natural capital should obtain certain right from the Chinese government. The government can give or lease some types of rights for the corporates to deal with the natural assetStrict management of “core national data” as stipulated in the Data Security LawLack of traceability of supply chainsBoth direct and indirect dependencies concernedIndirect dependences due to both the interactions among upstream/downstream industries and trans-ecosystem influencesCompound impacts which mixed with socio-economic impactsImpact also depending on the physical environment in each priority location |
| Assess (Material risks & opportunities) | | |
| Assess the risks to the business, any mitigation required and what material should be disclosed. | China’s new product level LCA database ^8^Commitment to circular economy in China ^9,10^Commitment to carbon neutrality in China ^11^ | Knowledge and skill challenges with new type of assessmentLack of knowledge and expertise due to the talent pool being insufficient in the short term |
| Prepare (To respond & report) | | |
| Allocate resources, set targets and present risk disclosure | Precedence of reporting and disclosing using “Legal Disclosure Report of Environmental Information” reports for the MME^12^Precedence of disclosing environmental impacts on MME’s “Enterprise Environmental Information Legal Disclosure System”Number of individual shareholders is relatively high | Some companies are not obliged to report if there are commercial secretsNo requirement for evidence-based or best-practice methodsNo requirement for assessment to be completed or verified by an independent assessor |

1. Su, Y. *et al.* An updated Vegetation Map of China (1:1000000). *Sci Bull (Beijing)* 65, 1125–1136 (2020).

2. Tan, J. *et al.* Preliminary assessment of ecosystem risk based on IUCN criteria in a hierarchy of spatial domains: A case study in Southwestern China. *Biol Conserv* 215, 152–161 (2017).

3. Meng, X. *et al.* Threatened Status Assessment of Multiple Grassland Ecosystems and Conservation Strategies in the Xilin River Basin, NE China. *Sustainability* 12, 1084 (2020).

4. Chen, G., Wang, X. & Ma, K. Red list of China’s forest ecosystems: A conservation assessment and protected area gap analysis. *Biol Conserv* 248, 108636 (2020).

5. People’s Republic of China Ministry of Environmental Protection (MEP), . *Technical Guide for Ecological Red Line. No. 2015.5.* (2015).

6. FABLE, . *Pathways to Sustainable Land-Use and Food Systems (China Chapter)*. (2020).

7. Zhao, J. & Xia, Q. China’s environmental labeling program. *Environ Impact Assess Rev* 19, 477–497 (1999).

8. Sichuan University China, . & IKE Environmental Technology CO Ltd China, . *Chinese Life Cycle Database (CLCD) (2009-2011)*. (2011).

9. Central Committee of the Communist Party of China (CPC). *The 13th Five-Year Plan For Economic And Social Development Of The People’s Republic Of China (2016–2020)*. (2020).

10. Bleischwitz, R. *et al.* The circular economy in China: Achievements, challenges and potential implications for decarbonisation. *Resour Conserv Recycl* 183, 106350 (2022).

11. Liu, Z. *et al.* Challenges and opportunities for carbon neutrality in China. *Nat Rev Earth Environ* 3, 141–155 (2021).

12. Ministry of Ecology and Environment. Measures for the Administration of Legal Disclosure of Corporate Environmental Information. *https://www.mee.gov.cn/xxgk2018/xxgk/xxgk02/202112/t20211221_964837.html?mc_cid=45e6a7ad33* (2021).
